# ERNICA evidence based guideline on omphalocele

**DOI:** 10.1186/s13023-026-04293-7

**Published:** 2026-03-07

**Authors:** Willemijn Irvine, Linde Margriet van der Kamp, Olivia Spivack, René Wijnen, Alberto Sgrò, Julia Brendel, Katrin Zahn, Lucas Matthyssens, Elisabet Gustafson, Henrik Røkkum, Lucia Migliazza, Rony Sfeir, Annika Mutanen, Udo Rolle, Anne Dariel, Marc Miserez, Ausra Lukosiute-Urboniene, Alexandre Vivanti, Nina Peters, Peter Conner, Eglė Machtejevienė, Francesca Russo, Ana Sanchez Torres, Alena Kokešová, Hans Jorgen Stensvold, Florian Kipfmueller, Mohamed Riadh Boukhris, Costanza Tognon, Simon Eaton, Iris den Uijl, Alexandra Benachi, Carmen Mesas Burgos

**Affiliations:** 1 Department of Pediatric Surgery, Erasmus Medical Center Sophia Children’s Hospital, Dr. Molewaterplein 40, Rotterdam, 3015 GD The Netherlands; 2 Dutch Institute for Clinical Auditing, Leiden, The Netherlands; 3 Department of Pediatric Surgery, University of Padua, Padua, Italy; 4 Department of Pediatric Surgery, Hannover Medical School, Hannover, Germany; 5 Department of Pediatric Surgery, Mannheim University Hospital, Mannheim, Germany; 6 Department of Pediatric Surgery, University Hospital Gent, Gent, Belgium; 7 Department of Pediatric Surgery, Uppsala University Hospital, Uppsala, Sweden; 8 Department of Pediatric Surgery, Oslo University Hospital, Oslo, Norway; 9 Department of Pediatric Surgery, Papa Giovanni XXIII Hospital, Bergamo, Italy; 10 Department of Pediatric Surgery, Lille University Hospital, Lille, France; 11 Department of Pediatric Surgery, Helsinki Children’s Hospital, Helsinki, Finland; 12 Department of Pediatric Surgery, Frankfurt University Hospital, Frankfurt, Germany; 13 Department of Pediatric surgery, La Timone Children’s Hospital, Marseille, France; 14 Department of Surgery, University Hospitals Leuven, Leuven, Belgium; 15 Department of Pediatric Surgery, Lithuanian University of Health Sciences, Kaunas, Lithuania; 16 Department of Obstetrics and Gynecology, Antoine Béclère Hospital Paris Sarclay University, Clamart, France; 17 Department of Obstetrics and Gynaecology - Division of Obstetrics and Fetal Medicine, Erasmus MC University Medical Centre—Sophia Children’s Hospital, Rotterdam, The Netherlands; 18 Department of Maternal and Fetal Medicine, Karolinska University Hospital, Stockholm, Sweden; 19 Department of Obstetrics and Gynaecology, Lithuanian University of Health Sciences, Kaunas, Lithuania; 20 Department of Obstetrics and Gynecology, University Hospitals Leuven, Leuven, Belgium; 21 Department of Neonatology, University Hospital La Paz, Madrid, Spain; 22 Department of Pediatric Surgery, University Hospital Motol, Prague, Czech Republic; 23 Department of Neonatology, Oslo University Hospital, Oslo, Norway; 24 Department of Neonatology, Bonn University Hospital, Bonn, Germany; 25 Department of Neonatology, Lille University Hospital, Lille, France; 26 Department of Neonatology, University of Padua, Padua, Italy; 27 27. Developmental Biology and Cancer Research and Teaching Programme, UCL Great Ormond Street Institute of Child Health, London, UK; 28 Department of Pediatric Surgery, Karolinska University Hospital, Stockholm, Sweden

**Keywords:** Omphalocele, Guideline, ERNICA, Prognosis, Management

## Abstract

**Background:**

Omphalocele is a congenital defect of the abdominal wall with high morbidity and high practice variation. Evidence based guidance on its management is currently absent. The European Reference Network for Rare Inherited and Congenital Anomalies (ERNICA) developed this guideline to aid clinical decision-making.

**Methods:**

This guideline was developed in accordance with the Guidelines 2.0 checklist and GRADE methodology. After a bottleneck analysis and prioritization, a systematic review of the literature and critical appraisal of the evidence was performed. Additionally, registry data from the European Pediatric Surgery Audit (EPSA) was provided in clinical questions for which published evidence was scarce. The Evidence to Decision framework was used as a guide to structure the consensus meetings and draft the recommendations.

**Results:**

The panel developed 12 recommendations on the following topics: Genetic screening, mode of delivery, prognostic factors, enteral feeding and ventilation during staged reduction, type of closure and timing of surgery in giant and non-giant omphalocele. The panel weighed up the benefits and harms, informed by all relevant arguments and expert opinion, to decide on a recommendation. The supplementary data from the EPSA contributed to the panel’s decision on a recommendation in four topics.

**Conclusion:**

This guideline provides recommendations for the perinatal care of patients with omphalocele. These recommendations support clinicians in making care decisions and help inform families about treatment options and relevant considerations. This guideline will be revised every five years to ensure it remains up to date.

**Supplementary Information:**

The online version contains supplementary material available at 10.1186/s13023-026-04293-7.

## Background

Omphalocele is a rare congenital abdominal wall defect in which abdominal organs herniate into the base of the umbilical cord, enclosed in a protective sac. The most recent registered prevalence rate in the European rare disease registration platform (EUROCAT) is 3.42 per 10.000 pregnancies [1]. While there is significant variation in clinical practice regarding prenatal diagnosis, delivery planning, and postnatal management, no evidence-based guideline exists today.

The European Reference Network for Rare Inherited and Congenital anomalies (ERNICA) is a clinical network dedicated to improving the quality of care for patients with rare digestive and gastrointestinal diseases, including omphalocele. As part of this European program, we aimed to develop an evidence-based clinical guideline, covering several aspects of pre- and post-natal care and closure of the abdominal wall. We aimed to set a standard for treatment and care that physicians, patients and their families can benefit from. The scope of this guideline is limited to perinatal care up to abdominal wall closure.

## Methods

This guideline was developed according to the GIN-McMaster Guidelines 2.0 checklist and GRADE methodology [2]. A complete description of the methodology is available in the additional files (Additional File 1). A multidisciplinary guideline development panel (GDP) including maternal and fetal medicine specialists, pediatric surgeons and neonatologists from all ERNICA hospitals that are accredited as expert centers for omphalocele collaborated in the development of this guideline. As omphalocele is such a rare disease and no formal patient support organizations exist, we failed to identify patient representatives willing to be part of the guideline development panel. Instead, three patient representatives that were suggested by participating clinicians contributed to the development process by giving written input at various stages throughout the development process. During the initiation phase, analysis, prioritization and listing of clinical gaps was done through email discussions and google forms and later refined during online and in-person meetings with the GDP. Thereafter, a preliminary list of key questions was circulated in the network for prioritization. Anyone interested could respond, and the final questions were selected based on highest rank for priority, see Table 1. Detailing of the questions in PICO format is displayed in the summary of findings and evidence to decision tables (Additional Files 2 and 3).

**Table 1:** Key questions addressed in this guideline

Module 1. Prenatal Care	1.1	Should Whole Exome Sequencing (WES) after karyotyping or chromosomal microarray (CMA) be offered to families expecting a child with omphalocele?
	1.2	Should vaginal delivery or cesarean delivery be advised for mothers expecting a child with suspected non-giant omphalocele?
	1.3	Are (1) liver herniation, (2) omphalocele size expressed in ratios and (3) lung volume on MRI prognostic factors for neonatal outcome in babies with omphalocele?
Module 2. Postnatal Care	2.1	Should the introduction of enteral feeding start before or after closure of the abdominal wall in patients that undergo staged reduction and surgical repair?
	2.2	Is spontaneous breathing or intubation and mechanical ventilation preferred during staged reduction in patients with giant omphalocele?
Module 3. Closure of the abdominal wall	3.1	Is non-operative management superior to staged reduction and surgical repair for patients with giant omphalocele?
	3.2	What substance should be used if a patient is treated with non-operative management for giant omphalocele?
	3.3	Are certain interventions to be preferred over others for staged reduction and surgical repair in giant omphalocele?
	3.4	Is timing of surgery related to outcome in primary closure of non-giant omphalocele?
	3.5	Is timing of surgery related to outcome in delayed repair of giant omphalocele?

**Fig. 1:**
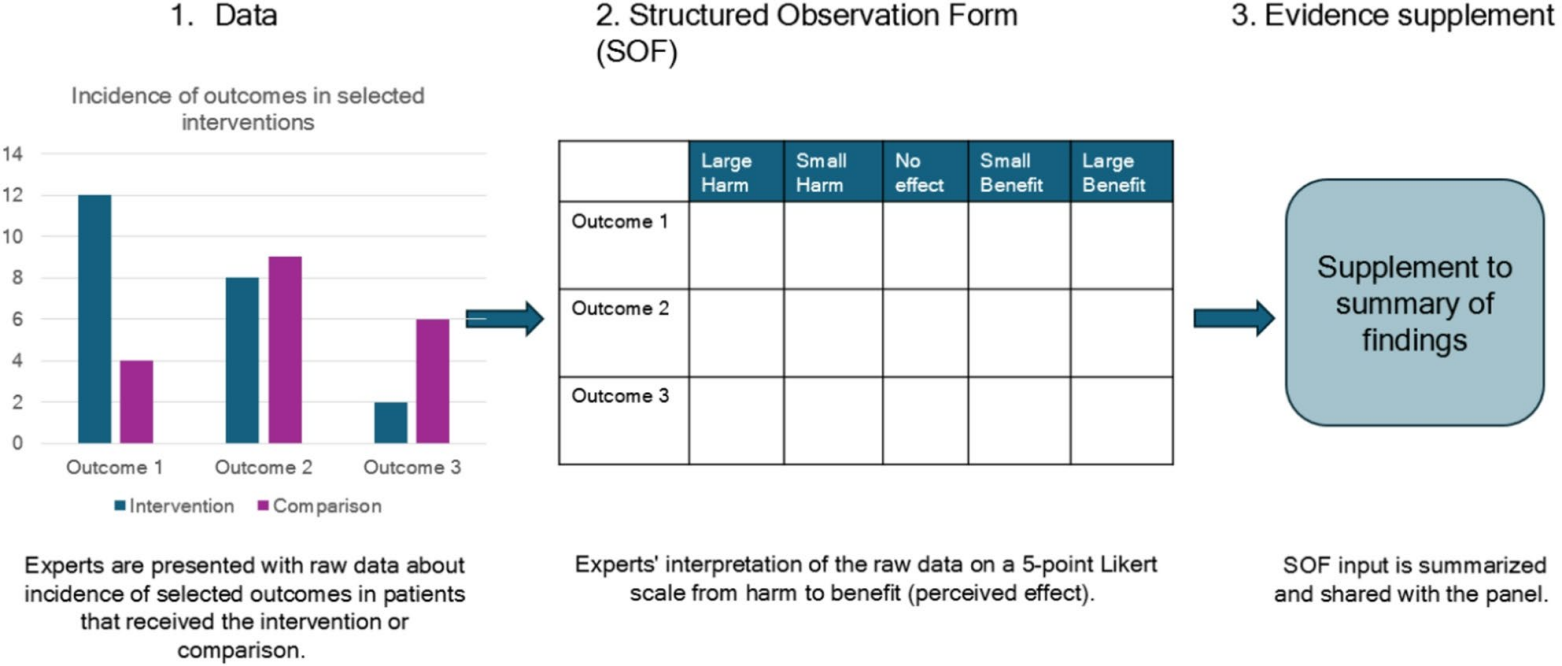
Structured observation of cases

A systematic search of the literature was done to identify relevant evidence for all clinical questions. The GDP pre-selected outcomes of interest to reflect the main concerns in omphalocele care and as we knew beforehand the evidence would be scarce, surrogate outcomes that could be allowed were discussed for all questions. Prioritized outcomes are included in the summary of findings and evidence to decision frameworks and (Additional Files 2 and 3). A detailed search strategy is also available in the additional files (Additional File 4). Original studies and systematic reviews published after 2000 and which included at least 5 patients with relevant information to one or more of the selected outcomes were eligible for inclusion. All included evidence was summarized, and the certainty of evidence was assessed according to GRADE [3]. As part of a pilot during the development of this guideline, panel members’ observations of registry data from the European Pediatric Surgery Audit (EPSA) were integrated as supplementary evidence, see Fig. 1 [4]. According to current legal arrangements, the data may be used for quality improvement, e.g. reviewed, analyzed and shared within the network, but can not be published. Therefore, panel members rated their perceived effectiveness of interventions based on EPSA data on a spectrum from harmful to beneficial by using structured observation forms (SOF), see Fig. 1. A summary of the SOFs was added as supplementary evidence to the summary of findings.

The Evidence to Decision framework [5] was used as a guide to structure a two-day face-to-face consensus meeting with most of the panel members (88%) present. During this meeting, the guideline development group selected a grade of recommendation between Strong or Conditional and a direction between supporting (for) or opposing (against) an intervention. In accordance with the GRADE methodology, low certainty of evidence does not exclude a strong recommendation, and weak recommendations are also possible with a high certainty of evidence. The strength of the recommendation is always determined by weighing up all relevant arguments. During a peer review phase various experts inside as well as outside our network were invited to review the guideline draft.

### Definitions

There is no consensus about the definitions used to describe giant and non-giant omphalocele subtypes, nor on the associated treatment options. For the purpose of this guideline, and without intending to standardize the use of these terms, the panel selected working definitions for the terms, see Table 2. A detailed explanation and implications of the chosen definitions for evidence selection are available in the additional files (Additional File 5).

**Table 2:** Definitions used for this guideline

Giant omphalocele	Defects that cannot be closed primarily and have a diameter of ≥5 cm where the liver is (partially) herniated
Non giant omphalocele	Defects that are <5 cm and typically do not contain liver
Primary Surgical Closure	Procedure in which the herniated contents are reduced back into the abdominal cavity, and the abdominal wall defect is closed directly
Staged Reduction and Surgical Repair	Involves a gradual reduction of the herniated contents using a silo or other method over several days to weeks, followed by definitive closure of the abdominal wall.
Non-Operative management and Delayed closure (NOM)	Abdominal tissue closure by epithelialization of the sac, with or without topical medication applied directly onto the omphalocele membrane and delayed closure of the abdominal wall.

## Results

All recommendations are displayed in Table 3. Full evidence to decision tables can be found in the additional files (S2). A revision of this guideline will be scheduled every 5 years following the ERNICA protocol for revising guidelines [6]. All recommendations are valid until revised.

**Table 3:** Summary of recommendations

Recommendation	Strength	Certainty of Evidence	EPSA data used
**Module 1. Prenatal Care**			
*The panel suggests offering Whole Exome Sequencing to all omphalocele patients (and when available*,* Whole Genome Sequencing).*	Conditional	Very Low	No
*The panel recommends vaginal delivery for non-giant omphalocele babies unless obstetrical considerations would warrant otherwise*	Strong	Very Low	Yes
*The panel suggests using extracorporeal (part/whole) liver herniation (assessed in the 2nd trimester) and the omphalocele circumference/abdominal circumference (OC/AC) ratio (measured from the 2nd trimester up until 32 weeks gestation) as prognostic factors but not as a single determinant.*	Conditional	Low	No
*The panel recommends a prenatal MRI for lung volume evaluation early in the 3rd trimester in case of suspected giant omphalocele.*	Strong	Moderate	No
*The panel suggests using Observed-to-Expected (O/E) total lung volume (TLV) calculated with fetal MRI according to the Meyers nomogram in prenatal counselling. An O/E < 50% seems to be associated with a higher risk of mortality and morbidity.*	Conditional	Low	No
***Good practice statement*** *Assessment of prognostic factors is preferably done in a research or registry setting*,* as prospective data collection is warranted to confirm the reliability of these measurements for predicting neonatal outcome*	-	-	-
**Module 2. Postnatal Care**			
*The panel recommends a pre-operative start of (at least trophic) enteral feeding in patients that undergo staged reduction*	Strong	-	Yes
*The panel suggest choosing either intubation or spontaneous breathing during staged reduction*,* depending on the condition of the patient (incl. pain levels) and the method used for staged closure*	Conditional	-	Yes
**Module 3. Closure of the abdominal wall**			
*The panel suggests either non-operative management with delayed closure or staged reduction and surgical closure for patients with giant omphalocele.*	Conditional	Very Low	Yes
*The panel suggests non-operative management with delayed closure for patients with respiratory distress*,* pulmonary hypertension or lung hypoplasia.*	Conditional	-	No
*The panel recommends refraining from using mercurochrome*,* silver solutions and povidone iodine for the non-operative management of giant omphalocele. The panel suggests the use of non-toxic substances (with less side effects) such as honey or saline instead.*	Strong	Very Low	Yes
*The panel suggests choosing interventions for staged closure based on the center’s experience*	Conditional	Very Low	No
*The panel recommends choosing the timing of surgery (in non-giant*,* non-ruptured omphalocele) based on the condition of the patient and the availability of a surgical team with relevant expertise; the procedure should not be considered an emergency.*	Strong	-	Yes
*The panel suggests planning delayed closure of the abdominal wall after initial non-operative treatment not before the patient reaches the age of 1 year old.*	Conditional	-	Yes

### Module 1: Prenatal care

#### Genetic testing

Recommendation: ***The panel suggests offering Whole Exome Sequencing (WES) to all omphalocele patients (and when available***,*** Whole Genome Sequencing [WGS]).***

Good practice statement: ***Parents should receive adequate pre-test counselling explaining the possible benefits/harms***,*** tailored to the local situation. Parents’ choice should be accounted for by obtaining informed consent.***

Evidence summary: Three studies were included to evaluate the diagnostic yield of WES for additional diagnoses [7–9]. The panel concluded that there are cautious indications that Whole Exome Sequencing (WES) has a slightly higher diagnostic yield in fetuses with isolated or non-isolated omphalocele compared to karyotyping and chromosomal microarray. Specifically for Beckwith-Wiedemann Syndrome, it is unclear what the diagnostic accuracy of WES is. The certainty of evidence was rated as ‘very low’, since all evidence came from observational studies, and there was high between-study heterogeneity detected in the included systematic review. There were also indications of publication bias. Furthermore, the systematic review by Mellis et al. [8] included all types of structural congenital anomalies, and was not able to draw any conclusions regarding WES specifically related to abdominal wall defects due to the low sample size.

Considerations and justification: Despite the low certainty of evidence, most panel members agreed that WES probably has an additional diagnostic yield which could be beneficial for both parents and caregivers. Identification of a syndrome with a big disease burden has potential benefits. It can help parents to make a decision about termination of pregnancy, but can also help parents and healthcare providers to prepare optimally for what is ahead. A specific diagnosis may also give information about the risk in future pregnancies. Thereby, qualitative research indicates that it is helpful for parents to have a specific diagnosis [10]. Considering the same sample used for karyotyping/CMA can be used for WES, the panel considered the possible undesirable effects, such as false negatives, or incidental findings, to be trivial, if parents are fully informed prior to testing and provide their consent. The panel issues a conditional recommendation since the benefits seem to outweigh the possible harms, yet the evidence base is limited. Although no direct evidence was available, the panel agreed that given the anticipated pace of innovation, Whole Genome Sequencing (WGS) could be offered in place of Whole Exome Sequencing (WES), if accessible.

#### Delivery

Recommendation: ***The panel recommends vaginal delivery for non-giant omphalocele babies unless obstetrical considerations would warrant otherwise***.

Evidence summary: Only one study could be included to evaluate the effects of mode of delivery on patients with a non-giant omphalocele [11]. Some small benefits in terms of length of stay were observed for vaginal delivery, but for outcomes mortality and time to full feeds, no significant differences were found. The certainty of this evidence was rated ‘Very Low’, because the evaluated evidence is derived from an observational study that does not correct for important confounders and raises concerns about imprecision due to the small sample size in the intervention (vaginal delivery) group. Supplementary evidence from structured observation based on EPSA data was used. From the EPSA data, outcomes mortality, ventilation time and time to full feeds were evaluated. Based on the presented data, most panel members observed some benefits of vaginal delivery regarding the outcome measures ventilation time and time to full feeds. Baseline demographics of both groups were comparable, but panel members had doubts regarding the correlation between mode of delivery and the evaluated outcomes.

Considerations and Justification: The certainty of evidence for the possible small benefits of vaginal delivery is very low; however, panel members agreed that both the published evidence, and the EPSA data, show no indication of significant harmful effects of vaginal delivery on neonatal outcome. The risk of rupturing the omphalocele with a vaginal delivery was discussed as a possible harm, but panel members agreed that this is not a realistic risk for patients with a non-giant omphalocele. On the contrary, avoiding caesarean delivery may carry significant beneficial effects for the mother. The panel members agree that the balance of effects probably favors vaginal delivery for non-giant omphalocele babies unless obstetrical considerations would warrant otherwise.

#### Prognostic factors

Recommendations: ***The panel suggests using extracorporeal (part/whole) liver herniation (assessed in the 2nd trimester) and the omphalocele circumference/abdominal circumference (OC/AC) ratio (measured from the 2nd trimester up until 32 weeks gestation) as prognostic factors but not as a single determinant.***

***The panel recommends a prenatal MRI for lung volume evaluation early in the 3rd trimester in case of suspected giant omphalocele.***

***The panel suggests using Observed-to-Expected (O/E) total lung volume (TLV) calculated with fetal MRI according to the Meyers nomogram in prenatal counselling. An O/E < 50% seems to be associated with a higher risk of mortality and morbidity. Assessment of such prognostic factors is preferably done in a research or registry setting***,*** as prospective data collection is warranted to confirm the reliability of these measurements for predicting neonatal outcome.***

Evidence summary: The panel evaluated prenatally detected liver herniation, omphalocele ratios and total lung volume (o/e TLV) on prenatal MRI as prognostic factors for neonatal outcome. For the factor prenatally detected liver herniation, five studies were included for analysis [12–16]. The literature was analyzed for outcomes neonatal mortality, adverse neonatal outcome and ability to perform primary repair. The panel concluded that there are indications that prenatally detected extra-abdominal liver is a predictor for a higher mortality rate and more adverse neonatal outcomes compared to intra-abdominal liver, and that there are indications that prenatally detected extracorporeal liver is an independent predictor for the inability to perform primary repair. Certainty of evidence for all outcomes was judged as ‘Low’, mainly due to the small series and possible selection bias. For the outcome adverse neonatal events, there were also some concerns for indirectness, as severe and less severe events were combined into a single outcome measure.

Omphalocele Circumference/Abdominal Circumference (OC/AC) and Omphalocele Diameter/Abdominal Circumference (OD/AC) were analyzed as prognostic factors for mortality, respiratory insufficiency and ability to perform primary repair based on seven studies [12, 15, 17–21]. The panel concluded that it is likely there is a significant negative association between the OC/AC ratio (measured between 17- and 38-weeks of gestation) and survival, and that the OC/AC ratio is significantly associated with the probability of requiring delayed or staged closure. Cut-off values between 0.57 and 0.75 have been suggested to be highly sensitive and specific and there is likely a trend towards more respiratory insufficiency in patients with higher OC/AC ratios [19, 21]. The optimal cut-off probably varies with gestational age at which the ratio is measured [22]. Certainty of evidence was rated as moderate for these outcomes and only rated down for not correcting for important confounders. Concerning the OD/AC ratio, the panel concluded that there are indications that OD/AC ratios > 0.26 are associated with a higher likelihood of death. They concluded that it is likely that an OD/AC ratio above 0.24–0.26 is predictive of the inability to perform primary repair of the omphalocele and that there are indications that an OD/AC ratio above 0.26 is predictive of intubation in the first 24 h of life (and the need for longer mechanical ventilation). One study looked into the correlation between the ratio of omphalocele diameter and transabdominal diameter (OD/TAD) and neonatal morbidity and indicated a correlation between ratios > 0.8. It found a significant increase in morbidity (hospitalization in the intensive care unit for more than 42 days, need for respiratory assistance (mechanical ventilation, intubation) for more than 21 days and/or need for parenteral feeding for more than 21 days) in patients with a OD/TAD ratio > 0.8 [23]. Unlike the other ratios, this study was based exclusively on measurements performed during the first trimester. Collectively, the certainty of evidence for these outcomes was judged as ‘Low’, since bias due to confounding as well as imprecision was suspected in almost all included studies.

The predictive value of total lung volume measured with prenatal MRI could be analyzed based on three studies [24–27]. The panel analyzed these studies for the outcome measures mortality, need for mechanical ventilation and duration of ventilation, need for tracheostomy and length of hospital stay. Lower O/E total lung volume on prenatal MRI (O/E TLV) is likely related to mortality, need for and length of mechanical ventilation and possibly the need for tracheostomy. As results between the two analyzed studies were conflicting, it is unclear if O/E TLV is predictive of length of hospital stay. The certainty of evidence for the outcomes mortality and intubation was rated as ‘moderate’. The evidence for these outcomes was only rated down for risk of bias due to retrospective design. For the remaining outcomes, the certainty of evidence was rated as ‘Low’ due to the retrospective nature of both studies, small sample sizes and event rates and for not correcting for confounders.

Considerations and Justification: The panel concludes that the balance of effects probably favors including liver herniation, the OC/AC ratio and the O/E lung volume as prognostic factors and that measurement of omphalocele ratios could provide prognostic information as early as the first trimester. As there are many confounding variables and the certainty of evidence is low to moderate, panel members emphasize that these factors alone are just one piece of the puzzle. The panel agrees that if these factors are used as prognostic indicators during counselling, they should not be considered independent predictors but should be considered in combination with other factors to get the most reliable prediction. Further prospective data collection showing the relationship between prenatal liver herniation, OC/AC ratio, O/E TLV and neonatal outcome is desirable and timing of measurement for ultrasound parameters should be standardized. Therefore, the panel suggests that these measurements are preferably collected in a research or registry setting. Strict instructions for timing of measurement, amount of repeat measurements and definitions for neonatal outcomes are imperative to establish a higher certainty of evidence on these measurements as prognostic factors. Including these variables in the revision of the EPSA dataset is a possibility, although there are barriers to the accurate registration of prenatal data as this is often included in the mother’s medical file only.

### Module 2: Postnatal care

#### Feeding

Recommendation: ***The panel recommends a pre-operative start of (at least trophic) enteral feeding in patients that undergo staged reduction.***

Evidence summary: No published evidence was found to answer this question. Supplementary evidence from structured observation based on EPSA cases was used. From the EPSA data, information on outcomes: time to full feeds, length of postoperative hospital stay, and postoperative complications was available. Based on the presented data, panel members did not observe substantial differences between the pre-operative and post-operative feeding groups.

Considerations and justification: Panel members agree that in general, earlier enteral feeding is preferable as it may be related to the prevention of parenteral nutrition/central line-related complications, oral aversion or atrophy. However, the condition of the baby will not always allow for the pre-operative start of enteral feeding, such as in cases of bowel obstruction. As this information is not recorded, interpretation of the EPSA data for these outcomes is difficult. Panel members do find it remarkable that in the pre-operative feeding group, the average time to surgery was much longer than in the post-operative feeding group and consider this could by biassed by the intention to treat. Time to start of enteral feeding could not be interpreted due to missing data. All things considered, panel members agree that there are no clear indications of negative effects of pre-operative feeding which suggests that pre-operative feeding is probably safe. Some evidence from other malformations such as gastroschisis, suggests that earlier feeding is probably related to some benefits [28–30]. It is likely that parents and caregivers will prefer a recommendation towards earlier feeding as they may associate feeding of the child with normalcy. Besides, being able to provide breastmilk as a feeding source is often highly valued by mothers. The panel agrees that, based on the balance of benefits and harms and the value parents likely place on early feeding, enteral feeding should be initiated prior to closure of the abdominal wall, even if only trophic feeds are tolerated by the patient.

#### Ventilation

Recommendation: ***The panel suggest choosing either intubation or spontaneous breathing during staged reduction***,*** depending on the condition of the patient (incl. pain levels) and the method used for staged closure.***

Evidence summary: No published evidence was found to answer this question. Supplementary evidence from structured observation based on EPSA cases was used. From the EPSA data, information on outcomes: time to full feeds, length of postoperative hospital stay, and days until surgery was available. Panel members discussed the value of these findings from the structured observations forms. Their collective opinion was that for this question, the EPSA data have limited added value. This is mainly because the observed effect for the various outcomes was inconsistent (indicated benefit for some and harm for others). Other arguments were the lack of a clear intention to treat and the small sample size that was analyzed (9 awake versus 12 ventilated patients with available data on outcomes).

Considerations and justification: As the intention to treat is unclear from the data, the panel members were asked about their considerations to decide on intubation. Based on their expert opinion, panel members express that the patient needs to be sedated during intubation and mechanical ventilation, and therefore the muscle tone of the patient is completely absent, which could ease the reduction of the content into the abdominal cavity. Some experts report giving muscle relaxants in awake babies, but we have no data to evaluate the effect of this practice. A second benefit is that intubation and mechanical ventilation allow for more options to control the patient’s pain and keep them comfortable. The panel also considered that in general, there are considerable undesirable effects of (prolonged) intubation and mechanical ventilation, and that general anaesthesia can have negative side effects for the child’s (neuro)development, especially under the age of one year old [31–34]. Thereby, giving the patients sedation or anaesthesia during intubation could negatively affect the chances for early enteral feeding. The panel therefore concludes that the downsides of ventilation are possibly large and that, if it is possible, awake reduction should at least be considered. In these cases, it is probably acceptable to keep patients on spontaneous breathing (extubated), but currently there are too little arguments to prove superiority of this over ventilation for all patients, as the feasibility of this approach strongly depends on the chosen intervention for staged reduction. For parents of the patient, awake reduction would only be acceptable if they can be assured that their child is not in pain. Patient representatives suggested informing parents about how pain and comfort during the treatment will be assessed and the possibility of having to resort to ventilation at some stage. If the patient is ventilated, parents also indicated that it is important they receive information about the possibilities to touch or hold their child while they are on the ventilator.

### Module 3: Closure of the abdominal wall

#### Staged reduction versus non-operative management and delayed closure

Recommendation: ***The panel suggests either non-operative management with delayed closure or staged reduction and surgical closure for patients with giant omphalocele***.

***The panel suggests non-operative management with delayed closure for patients with respiratory distress***,*** pulmonary hypertension or lung hypoplasia.***

Evidence summary: Three studies were included to evaluate the benefits and harms of non-operative management (NOM) versus staged reduction and surgical closure in giant omphalocele [35–37]. There are some indications that there is no significant difference in the risk of mortality after non-operative management versus staged reduction and surgical closure for giant omphalocele. There are some indications that there is no significant difference in length of stay after NOM versus staged reduction and surgical closure for giant omphalocele. There are some indications that NOM may result in shorter times to full enteral feeding compared to staged reduction and surgical closure. In addition to the published data, supplementary evidence from structured observation based on EPSA cases was used. From the EPSA data, information on outcomes: mortality, ventilation time, time to full feeds, length of post-operative hospital stay and post-operative complications was available. As in the published data, panel members observed some benefits of NOM on the outcome: time to full feeding. Even though the comparison was debatable, as surgeries take place at very different times in both groups, panel members also observed benefits in regards to the number of post-operative complications. The EPSA data suggest that there are possibly less post-operative complications in a delayed closure procedure compared to surgeries as part of a staged reduction strategy.

Considerations and justification: The certainty of evidence was rated as very low. Outcomes were not corrected for confounders in any of the studies. The study of Binet et al. [36] may also carry bias by study site, as all patients in the staged group were treated in a high resource hospital in France and all patients in the NOM group were treated in a lower resource hospital in the Ivory Coast, without a neonatal unit and lacking the technical means for resuscitation and anaesthesia. While the EPSA data points in the same direction for the outcome time to full feeds, the panel also sees a lot of confounding factors. Part of the panel indicated that the EPSA data increased their confidence in the conclusion for this outcome. Overall, the panel judged that, based on the analyzed literature and supplementary evidence from the EPSA, as well as their expert experience, there are no clear indications that one intervention should be preferred over the other. Based on expert consensus, panel members do agree that, for patients with respiratory distress, pulmonary hypertension or lung hypoplasia, the balance between benefits and harms favors non-operative management and delayed closure.

#### Substances for non-operative management

Recommendation: ***The panel recommends refraining from using mercurochrome***,*** silver solutions and povidone iodine for the non-operative management of giant omphalocele. The panel suggests the use of non-toxic substances (with less side effects) such as honey or saline instead.***

Evidence summary: One systematic review was included to answer this question [38]. In this review, effects of honey (53 patients), 2% aqueous eosin (271 patients), Gentian violet (47 patients), silver dressings/solution (136 patients), povidone iodine (98 patients), mercurochrome (91 patients), saline (18 patients), dry dressing only (75 patients) and mixed agents (42 patients) were explored. There is no evidence to indicate superiority of one agent over another for the outcomes: time to epithelialization and amount or severity of complications. There are some indications that less/non-toxic agents like honey and saline have similar performance to well performing toxic agents, while decreasing toxicity risk. In addition to the published data, supplementary evidence from structured observation based on EPSA cases was used. If NOM was performed, data was stratified into use of a dry cover, wound dressing or other. Looking at the outcome days until surgery the majority of the panel observed longer times to surgery in the group ‘dry cover’ compared to groups ‘wound dressings’ and ‘other’. The clinical impact of this is probably limited.

Considerations and justification: The presented evidence is based on a recent systematic review. Many studies included in the review are of poor quality. The supplementary evidence based on observations from EPSA data was also considered of poor quality due to small sample sizes of the compared groups and many missing values. It was difficult for the panel to make a judgement on beneficial effects as no substances were directly compared in the available literature, the evidence quality was poor and the EPSA data was very scattered with lots of missing values. Overall, panel members agree that mercurochrome, silver solutions and povidone iodine probably have more undesirable effects compared to others. As there are no indications these substances with possible toxic side effects perform better compared to non-toxic substances like honey and saline, the panel agrees that they should be avoided.

#### Staged closure

Recommendation: ***The panel suggests choosing interventions for staged closure based on the center’s experience.***

Evidence summary: Ten studies were included to evaluate the benefits and harms of different interventions for staged closure [36, 39–47]. The research evidence was too heterogeneous to pool or directly compare any of the outcomes. Panel members were therefore presented with a matrix of staged closure methods and outcomes. Included interventions were surgical silo [36, 44], non-surgical silo (DuoDerm) [39, 40], Barlow external silo [43], patch sutured over the omphalocele [45], Fasciotens^®^ [47], traction [46], taping [42] and suspension [41]. The matrix is included in supplement S2.

Considerations and Justification: The panel members could not identify any clear benefits or harms of one method over the other. To make better judgements, ‘staged reduction’ needs to be defined better, as for some included interventions panel members were not in agreement whether they would call it a ‘staged’ procedure. Panel members concluded that they have too little information to indicate the balance of effects in favor of one or more specific interventions. As the staged procedures are carried out infrequently, most panel members agree that that the choice of an intervention is not so much based on outcomes, but more on experience. While care equity may be decreased as practice variation is high, panel members emphasize that all the studied interventions are complex and require specific expertise. Specific expertise in one intervention and consistently practicing this intervention makes it more likely that a surgical team can provide good quality of care, regardless of the method. A recommendation for either of the interventions may therefore increase equity.

#### Timing primary closure

Recommendation: ***The panel recommends choosing the timing of surgery (in non-giant***,*** non-ruptured omphalocele) based on the condition of the patient and the availability of a surgical team with relevant expertise; the procedure should not be considered an emergency.***

Evidence summary: No studies on the optimal timing of primary closure could be included. Supplementary evidence from structured observation based on EPSA cases was used. In the presented EPSA data, cases were stratified according to timing of surgery in an early and a late primary closure group. All selected patients were entered into the registry as non-giant omphaloceles. The early group had surgery on days 0, 1 or 2 of life. The late group had surgery on day 3 of life or later, but was entered into the registry as having primary closure. The outcomes mortality, post-operative complications, length of post-operative hospital stay and time to full feeds could be analyzed from the EPSA data. The majority of the panel members observed no important differences between groups for either of the outcomes. Some panel members indicated they observed less complications in the early group. There was a higher rate of respiratory failure and cardiac malformations in the late group.

Considerations and justification: When discussing the outcomes of the structured observation forms, panel members started to question the intention to treat in both groups. Decisions on the timing of surgery could be based on patient characteristics, but also on organizational factors such as the availability of beds or the right team. Bearing in mind that none of the panel members observed differences in mortality between groups or in post-operative hospital stay and most panel members did not see differences in post-operative complications between groups, there seems to be little reason to perform early surgery or to consider the surgery as an emergency procedure. Panel members agree that besides patient characteristics, such as respiratory status or comorbidities that warrant attention, the local organization of care has a role in the timing of surgery, and that waiting for the best team to be available could increase the quality of care. Not considering primary closure of a non-giant omphalocele as an emergency procedure could also increase care equity, as planned surgery with the right team decreases the risks attached to emergency procedures.

#### Timing of surgery for delayed closure after non-operative management of giant omphalocele

Recommendation: ***The panel suggests planning delayed closure of the abdominal wall after initial non-operative treatment not before the patient reaches the age of 1-year old.***

Evidence summary: No studies on the optimal timing of delayed closure after initial non-operative treatment could be included. Supplementary evidence from structured observation based on EPSA cases was used. In the presented EPSA data, cases were stratified according to timing of surgery in an early and a late delayed closure group. All selected patients were entered into the registry as giant omphaloceles. The early group had surgery before one year of age. The late group had surgery after one year of age. The outcomes post-operative complications and post-operative hospital stay could be analyzed from the EPSA data. The majority of the panel members observed no important differences between groups for either of the outcomes. Some panel members indicated that they observed a shorter length of stay and fewer complications in the late group. There was a higher rate of cardiac malformations and prematurity in the late group.

Considerations and Justification: When discussing the outcomes of the structured observation forms, panel members started to question the intention to treat in both groups. Panel members discussed that comorbidity, as well as choice of timing of surgery may have affected the observed outcomes. Patients in the late group (> 1 year of age) had more cardiac anomalies and a higher rate of prematurity, but no important differences in length of stay or complications were seen and some panel members even observed less complications and a shorter length of stay in this group. The condition of the patients may have been a confounder for these outcomes. Panel members discussed that this may plea in favor of surgery after 1 year of age. Neonatologists on the panel emphasize that elective surgery with general anaesthesia in the first year of life may have negative effects on neurological development, an outcome which is not considered in the evaluated data. While we do not have evidence for this in the omphalocele population, evidence is present for pediatric patients with other surgical conditions such as congenital diaphragmatic hernia and esophageal atresia [48–52]. Based on the evaluated EPSA data and expert experiences, there seem to be little benefits of performing the delayed closure before one year of age and minimal to no harms of postponing to after one year of age. Meanwhile, surgery after one year may be associated with shorter length of post-operative hospital stay. Some parents may favor earlier surgery, as closure of the abdominal wall and reconstruction of the umbilicus is associated with the feeling of normalcy. The personal preference of parents should be considered in the decision about surgical timing and hence, the panel issued a conditional recommendation.

## Discussion

This guideline aims to standardize the management of omphalocele by providing evidence-based recommendations for clinicians, as well as for patients and their families. It addresses twelve clinical questions encompassing pre-and postnatal care, and abdominal wall closure, supporting informed decision-making throughout the course of perinatal management. Similar to other pediatric surgical conditions, the body of evidence regarding omphalocele is both limited and generally of low quality [53–56]. The vast majority of included studies exhibit significant methodological shortcomings. Some of these issues stem from the inherent challenges that come with researching this disease [38] while others are due to suboptimal study design [35, 37]. As a result, the certainty of evidence, as assessed using the GRADE approach, was predominantly rated as low or very low. To mitigate against the scarcity of available evidence, we employed an innovative methodology, designed to strengthen the guideline development process for rare diseases. This method allowed us to integrate insights based on real-world data from the European Pediatric Surgical Audit (EPSA) into our guideline development process [4] and marks ERNICA’s first effort to integrate registry data into guideline development, supporting a broader, cyclical approach to quality improvement ([6], L.M. van der Kamp et al. 2025, *Fostering continuous quality improvement in a European rare disease network: Where are we now?*, Submitted for publication to Orphanet Journal of Rare Diseases). The approach reduced reliance on expert opinion alone for several clinical questions, thereby supporting more of a data-led recommendation process.

In addition to its rigorous development process, this guideline has several notable strengths. A multidisciplinary cross-European panel contributed to the development of the recommendations. A draft version of this guideline was also circulated for peer review, involving experts both within and outside of the network. The panel included content experts from maternal–fetal medicine, neonatology, and pediatric surgery, as well as a methodologist and an implementation coordinator. While the methodologist supported the panel in applying evidence-based guideline methodologies, the implementation coordinator helped to ensure that practical aspects related to implementation were identified and considered throughout the development process. An upcoming qualitative study will further explore the factors foreseen to hinder and/or facilitate implementation of several of the omphalocele guideline recommendations in clinical practice. This will be done with a view to selecting appropriate implementation strategies.

At the start of this guideline development process, no formal, evidence-based guidelines or recommendations for the management of omphalocele were available. Since then, a consensus statement has been published by the European Pediatric Surgeon’s Association (EUPSA) [57]. This document presents informal expert consensus on both surgical and non-surgical treatment, as well as prognostic factors influencing treatment outcomes. With this publication, the EUPSA made an important contribution to the field, by listing the current evidence on surgical and non-surgical treatment and summarizing it in light of the opinions of experienced clinicians and content experts. While there is overlap in the areas of care addressed and conclusions made, our guideline provides actionable recommendations and offers transparency, explaining the panel’s decision-making process, which was grounded in evidence, registry data, and a rigorous assessment of the balance between benefits and harms in response to specific predefined questions. To ensure coherence and avoid duplication in future revisions, efforts will be made to align guideline development processes between ERNICA and EUPSA (L.M van der Kamp et al. 2025, *Fostering continuous quality improvement in a European rare disease network: Where are we now?*, Submitted for publication to Orphanet Journal of Rare Diseases).

Through summary and extensive discussion of the available evidence, this guideline development process also shed light on current research gaps and priorities particularly in areas such as nutritional strategies and respiratory support in the management of omphalocele. Questions remain as to whether early enteral feeding improves growth outcomes and whether mechanical ventilation facilitates faster closure of the abdominal wall during staged repair. Yet current evidence is limited and subject to significant selection bias, as clinical decisions such as initiating ventilation or delaying feeding, are often driven by underlying patient characteristics and disease severity. To generate more reliable evidence in the future, prospective studies with robust design are essential. Where randomization is not feasible, advanced analytical methods such as propensity score matching or adjustment can be used to control for confounding in observational data [58]. Registry-based studies, particularly using platforms like EPSA, offer an efficient and prospective way to collect real-world data across institutions and countries, especially when linked to clearly defined clinical questions [59]. In rare diseases, this approach may be the most feasible alternative to randomized controlled trials.

We intend to employ the European Pediatric Surgical Audit (EPSA) as a mechanism to monitor guideline implementation. Data points aligning with the recommendations are therefore to be included in the EPSA omphalocele data set. This will allow us to monitor levels of guideline adherence, alongside other markers of care quality. This guideline will be assessed for possible revision in 5 years time (2030).

## Conclusions

This guideline provides evidence-based recommendations on pre- and postnatal management and closure of the abdominal wall for patients with omphalocele. These recommendations are intended to support clinicians in making informed decisions and may also help inform families about available treatment options and important factors to consider. In addition to guiding current practice, the guideline development process has highlighted important research priorities to inform future studies. This guideline will be reviewed and updated every five years to ensure it remains current and clinically relevant.

## Supplementary Information

Below is the link to the electronic supplementary material.

Supplementary Material 1

Supplementary Material 2

Supplementary Material 3

Supplementary Material 4

Supplementary Material 5

## Data Availability

According to current legal arrangements, EPSA data may be used for quality improvement, e.g. reviewed, analyzed and shared within the network, but can not be published. Experts’ interpretation of this data as captured in the SOFs is available on request from the corresponding author. All other data analyzed during this study are included in this published article and its supplementary information files.

## Abbreviations

ERNICA: European Reference Network Inherited and Congenital Anomalies
EPSA: European Pediatric Surgery Audit
GRADE: Grading of Recommendations Assessment, Development and Evaluation
GDP/Panel: Guideline Development Panel
SOF: Structured Observation Forms
PICO: Patient Intervention Comparison Outcome
WES: Whole Exome Sequencing
CMA: Chromosomal Microarray
WGS: Whole Genome Sequencing
OC/AC: Omphalocele Circumference/Abdominal Circumference
OD/AD: Omphalocele Diameter/Abdominal Diameter
OD/TAD: Omphalocele Diameter/Transabdominal Diameter
O/E TLV: Observed-to-Expected Total Lung Volume
