# Supplementary material for: ERNICA evidence based guideline on omphalocele
Source: Orphanet J Rare Dis. 2026 Mar 7;21:193. doi: 10.1186/s13023-026-04293-7 (PMC13162432; doi:10.1186/s13023-026-04293-7)
Supplement: Supplementary file 1 — Supplementary Material 1 [file 13023_2026_4293_MOESM1_ESM.pdf]

Supplement 1. Methods

1. **Aim and target audience guideline**

The aim of this guideline is to provide recommendations on various multidisciplinary aspects of care for omphalocele patients and their families. The guideline is targeted to all European health care providers that are involved in the care for omphalocele patients both pre- and postnatally. This guideline is not specifically targeted to, but can be informative for, families and caregivers of patients.

2. **Validity of the guideline**

This guideline is valid from the moment of publication, until the publication of its update. In general, a guideline will undergo evaluation every 5 years. Consequently, the board of the ERNICA will install a new workingroup in 2030 to evaluate which modules are need of an update and if there is a need to develop new modules based on the latest insights. ERNICA is primarily responsible for the validity of the guideline and timely revision. The cooperating expert centres for omphalocele share the responsibility to inform ERNICA if new developments in research or clinical practise raise the need for evaluation before the 5 year mark.

3. **Patient population**

This guideline covers the care for omphalocele patients from prenatal diagnosis until postnatal intervention to close the abdominal wall. Long term consequences and cosmetic interventions are not included as topics of interest in this guideline.

According to the Orphanet, Snomed, ICD10 and ICD11 coding systems, this considers:

ORPHA: 660, 695032, 695038

SNOMED: 64176007

ICD 10: Q79.2

ICD-11: LB01

4. **Guideline development group**

A multidisciplinary guideline development panel (GDP) including 5 maternal and fetal medicine specialists, 6 neonatologists, 12 pediatric surgeons, a methodologist, an EPSA project manager and an implementation coordinator was selected from all ERNICA hospitals that are accredited as expert centres for omphalocele. As omphalocele is such a rare disease and no formal patient support organizations exists, we failed to identify patient representatives willing to be part of the guideline development panel. Instead, two patient representatives that were suggested by participating clinicians contributed to the development process by giving written input at various stages throughout the development process. The guideline development group consisted in total of 26 experts from 12 European countries.

Table A. The guideline development group

Professional		Affiliation and country	GDG position
Carmen Mesas Burgos, MD, PhD, FEBPS	Consultant Paediatric Surgeon, Associate Professor	Karolinska University Hospital, Stockholm – Sweden	Chair
Prof. Alexandra Benachi, MD, PhD	Maternal and Fetal Medicine specialist	Assistance Publique-Hopitaux de Paris. Hopital Antoine Béclère. Université	Chair

		Paris Saclay. CRMR Hernie de Coupole Diaphragmatique. Clamart. France	
Willemijn Irvine, MSc	Guideline Methodologist	Qualicura, Breda<br><br>Erasmus MC Sophia Childrens Hospital, Rotterdam – the Netherlands	Methodologist
Simon Eaton	Professor of pediatric surgery, nutrition and metabolism	UCL, London – United Kingdom	Panel expert
Marc Miserez	Abdominal Surgeon	Leuven UZ – Belgium	Panel expert
Julia Brendel	Pediatric Surgeon	Hannover Medical School – Germany	Panel expert
Katrin Zahn	Pediatric Surgeon	Universitätsmedizin Mannheim – Germany	Panel expert
Lucas Matthyssens	Pediatric Surgeon	UZ Gent – Belgium	Penel expert
Alberto Sgrò	Pediatric Surgeon	University of Padua – Italy	Panel expert
Rony Sfeir	Pediatric surgeon	CHRU, Lille – France	Panel expert

Elisabet Gustafsson	Pediatric Surgeon	Akademiska Sjukhuset Uppsala - Sweden	Panel expert
Henrik Røkkum	Pediatric Surgeon	Oslo University Hospital – Norway	Panel expert
Lucia Migliazza	Pediatric Surgeon	ASST Papa Giovanni XXIII di Bergamo – Italy	Panel expert
René Wijnen	Pediatric Surgeon	Erasmus MC Sophia Childrens Hospital, Rotterdam – the Netherlands	Panel expert
Annika Mutanen	Pediatric Surgeon	Helsinki Children's Hospital – Finland	Panel expert
Udo Rolle	Pediatric Surgeon	Universitätsklinikum Frankfurt – Germany	Panel expert
Anne Dariel	Pediatric Surgeon	CHU de Marseille - Hopital de la Timone – France	Panel expert
Ausra Lukosiute-Urboniene	Pediatric Surgeon	Hospital of Lithuanian University of Health Sciences Kauno Klinikos	Panel expert
Alexandre Vivanti	Maternal and Fetal Medicine specialist	GHU Paris-Sud - Hôpital Antoine Béclère	Panel expert

Nina Peters	Maternal and Fetal Medicine specialist	Erasmus Medical Center, Rotterdam – The Netherlands	Panel expert
Peter Conner	Maternal and Fetal Medicine specialist	Karolinska University Hospital	Panel expert
Eglė Machtejevienė	Maternal and Fetal Medicine specialist	Lithuanian University of Health Sciences– Lithiuania	Panel expert
Francesca Russo	Maternal and Fetal Medicine specialist	UZ Leuven – Belgium	Panel expert
Ana Sanchez Torres	Neonatologist	Hospital Universitario La Paz, Madrid – Spain	Panel expert
Alena Kokesova	Neonatologist	University Hospital Motol, Prague, Czech republic	Panel expert
Hans Jorgen Stensvold	Neonatologist	Oslo University Hospital	Panel expert
Florian Kipfmueller	Neonatologist	Universitatsklinikum Bonn	Panel expert
Mohamed Riadh Boukhris	Neonatologist	CHRU Lille – France	Panel expert
Constanza Tognon	Neonatologist	University of Padua	Panel expert

Iris den Uijl	ERNICA Project manager	Erasmus MC Sophia Childrens Hospital, Rotterdam – the Netherlands	Project support and management
Olivia Spivack	Implementation coordinator	Erasmus MC Sophia Childrens Hospital, Rotterdam – the Netherlands	Panel expert on implementation
Linde Margriet van der Kamp	EPSA project manager	Erasmus MC Sophia Childrens Hospital, Rotterdam – the Netherlands and Dutch Institute for Clinical Auditing	EPSA data specialist

Table 1: Guideline development group members and their affiliations.

All members of the steering group declared their interests. None of the GDG members had conflicting interests for this guideline. Completed declaration of interest forms of all GDG members are available on request at the ERNICA office.

5. **Implementation**

The implementation of the guideline and the practical feasibility of the recommendations were taken into account during the different phases of guideline development. In doing so, explicit consideration was given to factors that could promote or hinder the implementation of the guideline in practice. Specific barriers and facilitators to applicability of the recommendations are included in the evidence to decision frameworks (EtD) if relevant.

6. **Analysis of clinical care gaps**

During the initiation phase, analysis, prioritization and listing of clinical gaps was done through email discussions and google forms and later refined during online and in-person meetings. The highest ranking questions were selected to be part of the guideline.

7. **Questions and outcomes**

The analysis of clinical care gaps formed the basis for the questions for the guideline. The GDP chose to address prenatal as well as postnatal care topics. The GDG members pre-selected outcomes of interest to reflect the main concerns in omphalocele care and as we knew beforehand the evidence would be scarce, surrogate outcomes that could be allowed were discussed for all questions. All questions were refined and structured according to the PICO framework.

8. **Literature search and selection of literature**

A systematic literature search was conducted by a professional biomedical information specialist in the Erasmus Medical Center. The search was conducted in Medline (all), EMBASE and Cochrane. The full search strategy is available in supplement S4. The methodologist and chairs double blinded screened all results (n =808) based on title and abstract and excluded the following:

- cross-sectional studies
- animal studies
- in vitro studies
- Original studies with <5 included patients
- Articles published before 2000 (if there were no publications found published after 2000, this criterion was dropped)
- Case reports

- Expert opinion
- Letters to the editor
- Editorials
- Reviews

Eligibility was assessed based on a match with one or more PICO questions and their outcomes of interest.

9. **Pilot intervention**

In this guideline we piloted the use of supplementary evidence from registry data as assessed by clinical experts with structured observation forms (SOFs). Available EPSA data variables were matched to the clinical questions to be able to present data relevant to the clinical questions. At the time of data extraction, the EPSA database contained data on 251 omphalocele patients included from 2013 till 2023. However, comparisons were made between much smaller groups due to the specificity of the clinical questions. As a result, we presented panel members with an overview of raw, uncorrected data. For each clinical question, an overview of patient characteristics and outcomes was provided for each intervention group. Based on the method suggested by Pai et al. [1] experts rated their perceived effectiveness based on the presented EPSA data on a spectrum from harmful to beneficial on a Likert type scale, see figure 1.

Figure 1. Structured observation of cases

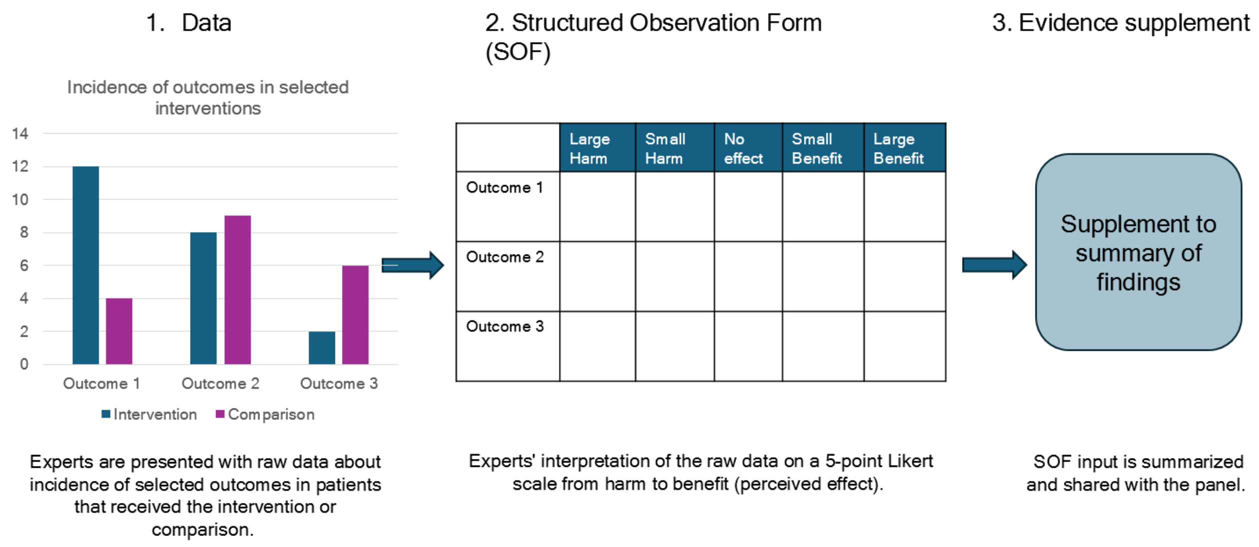

A summary of the experts' observations was provided next to a summary of literature (if available) during the 'evidence to decision'-phase. An example of a completed SOF is available at the end of this supplement (S1b).

10. **Summary of findings**

Data extraction and bias assessment was performed by the and reviewed by a panel expert. Any disagreement between reviewers was resolved by either discussion or by consulting a third reviewer if necessary. Based on the 'Grading Recommendations Assessment, Development and Evaluation' (GRADE) methodology [2] , all of the evidence on one outcome of interest, was collectively assessed as a 'body of knowledge' to determine the quality of evidence for that outcome. The quality of the body of knowledge was assessed following the GRADE methodology. GRADE results in the categorization of evidence into four levels: high, moderate, low and very low. These levels can be interpreted according to Table B.

Table B. Grading of Recommendations, Assessment, Development, and Evaluations

GRADE	Definition
High	We are very confident that the true effect lies close to that of the estimate of the effect.
Moderate	We are moderately confident in the effect estimate: The true effect is likely to be close to the estimate of the effect, but there is a possibility that it is substantially different
Low	We are limitedly confident in the effect of estimate: The true effect may be substantially different from the estimate of the effect.
Very Low	We have very little confidence in the effect estimate: The true effect is likely to be substantially different from the estimate of effect

All summary of findings tables are in the supplementary material (S2)

11. **Considerations and recommendations**

To determine the strength and direction of a recommendation, the Evidence to Decision(EtD) framework including ‘panel voice’ was used . The purpose of EtD frameworks is to help people use evidence in a structured and transparent way to inform decisions in the context of clinical recommendations and other health system or public health decisions [3]. The EtD framework was used as a guide to structure a two days consensus meeting in Stockholm, Sweden (November 2024) with most panel members present. To prepare for this meeting, a summary of findings of all modules was distributed among panel members, accompanied by an electronic survey covering all the aspects of the evidence to decision framework (panel voice). This survey prepared panel

members for the way of thinking that is necessary to discuss recommendations according to the evidence to decision framework and highlighted important discussion point upfront. Panel members that could not participate in the meeting, also received this survey so that their insights could be included in the discussion. During the consensus meeting, considerations, recommendations and research needs following the literature analysis and panel voice survey were discussed. A summary of the completed SOFs was presented as an evidence supplement and discussed alongside the summary of findings based on published literature. During this two day consensus meeting the evidence to decision framework led the GDP to a collective agreement on the type of recommendation for all PICO questions. The GDP decided between five recommendation options and chose uniform wording of recommendations for each of them as displayed in Table C. The exact wording of the recommendations was later agreed upon through email and electronic voting.

Table C. Types of recommendations and their wording

Type of recommendation	Wording
**Strong recommendations against the intervention**	The panel recommends against / to refrain from
**Conditional/weak recommendation against the intervention**	The panel suggests against / to refrain from
**Conditional/weak recommendation for either the intervention or the comparison**	The panel suggests either … or …

**Conditional/weak recommendation for the intervention**	The panel suggests ....
**Strong recommendation for the intervention**	The panel recommends ....

In accordance with the GRADE method, a low probative value of conclusions in the systematic literature analysis does not exclude a strong recommendation in advance, and weak recommendations are also possible with a high probative value. The strength of the recommendation is always determined by weighing all relevant arguments.

In addition to evidence-based recommendations, this guideline includes Good Practice Statements (GPS). These are recommendations that are considered essential for clinical practice but are not directly supported by a systematic review of the evidence. GPS are typically based on expert consensus, ethical considerations, or widely accepted standards of care.

All main outcomes and considerations for each question are summarized in the modules, full EtD tables are available in the supplementary materials (S3).

12. **Evaluation and authorization**

A draft of the guideline was submitted to all expert centres and patient representatives involved in the care for patients with omphalocele and affiliated with ERNICA. Comments from this peer review were collectively discussed within the GDP, resulting in adaptations and refinements of the guideline. Experts from non-omphalocele centres were also invited for the commentary round, as well as international specialists not associated with ERNICA. All reviewer comments were summarized and reviewers received a rebuttal that included either confirmation their comments were included in the revised guideline or a motivation for not including their suggestion. During the

authorisation phase the guideline was approved by all ERNICA registered omphalocele expert centres.

13. **Implementation &monitoring**

A central implementation support team (the ERNICA CIST) has been initiated within ERNICA. This team is dedicated to supporting the successful implementation of ERNICA's guideline recommendations in clinical practice. An upcoming qualitative study will further explore the factors foreseen to hinder and/or facilitate implementation of several of theomphalocele guideline recommendations. This will be done with a view to selecting appropriate implementation strategies. We intend to employ the European Pediatric Surgical Audit (EPSA) as a mechanism to monitor guideline implementation. Data points aligning with the recommendations are therefore to be included in the EPSA omphalocele data set. This will allow us to monitor levels of guideline adherence, alongside other markers of care quality. This guideline will be assessed for possible revision in 5 years time (2030). If possible, we intend to use EPSA data to inform the need for/scope of revision

[1] Pai M, Yeung CHT, Akl EA, Darzi A, Hillis C, Legault K, et al. Strategies for eliciting and synthesizing evidence for guidelines in rare diseases. BMC Med Res Methodol 2019;19(1):67.
[2] Guyatt GH, Oxman AD, Vist GE, Kunz R, Falck-Ytter Y, Alonso-Coello P, Schünemann HJ. GRADE: an emerging consensus on rating quality of evidence and strength of recommendations. Bmj 2008;336(7650):924-6.
[3] Moberg J, Oxman AD, Rosenbaum S, Schünemann HJ, Guyatt G, Flottorp S, et al. The GRADE Evidence to Decision (EtD) framework for health system and public health decisions. Health research policy and systems 2018;16:1-15.

Supplement 1b

Structured observation form

PICO 2: Should babies with a small omphalocele be delivered vaginally or via elective cesarean section?

P= Omphalocele

I = Vaginal delivery

C= Elective cesarean section

O= Mortality, Morbidity

EPSA input

1. Based on the presented data from the EPSA, what is your perceiving of the effect* of ***vaginal delivery*** on:

	Large benefit	Small benefit	No effect	Small harm	Large harm	Missing information / Don't know
Mortality		x				
Duration of mechanical ventilation		x				
Time to first feeds			x			
Time to full feeds		x				

2. To your perceiving, are the expected benefits/harms substantially different for different subgroups/for patients with specific characteristics?

> The data does not suggest that vaginal delivery is generally considered to have a significant advantage. Since patients with the liver in the omphalocele are more often delivered by elective cesarean section, this implies that clinicians have likely used this as the basis for decisions regarding the mode of delivery. Therefore, there is a certain difference in terms of patient characteristics. However, the data shows that vaginal delivery works excellently for a large group of patients and can therefore often be recommended.

3. Any other remarks or important considerations?

> No
