# Supplementary material for: ERNICA evidence based guideline on omphalocele
Source: Orphanet J Rare Dis. 2026 Mar 7;21:193. doi: 10.1186/s13023-026-04293-7 (PMC13162432; doi:10.1186/s13023-026-04293-7)
Supplement: Supplementary file 2 — Supplementary Material 2 [file 13023_2026_4293_MOESM2_ESM.pdf]

Supplement 2. Summary of findings

Summaries and grading of published literature on omphalocele

PICO 1.1: What are the additional benefits of whole exome sequencing (WES) after Chromosomal Microarray (CMA) and karyotyping as genetic screening tool in fetuses with Omphalocele?

Whole exome sequencing (WES), Chromosomal Microarray (CMA) and karyotyping for genetic screening in fetuses with Omphalocele

Bibliography: Shi et al. 2021, Que et al. 2023, Mellis et al. 2022

Certainty assessment							Summary of findings		
Participants (studies) Follow-up	Risk of bias	Inconsistency	Indirectness	Imprecision	Publication bias	Overall certainty of evidence	Study event rates (%)		Impact
							With Chromosomal Microarray (CMA)	With Whole exome sequencing (WES)	
**Diagnostic yield**									

Whole exome sequencing (WES), Chromosomal Microarray (CMA) and karyotyping for genetic screening in fetuses with Omphalocele

Bibliography: Shi et al. 2021, Que et al. 2023, Mellis et al. 2022

Certainty assessment							Summary of findings
4359 (3 non-randomised studies)	serious[a]	serious[b]	serious[c]	not serious	publication bias strongly suspected[d]	⊕◯◯◯ Very low	Three studies could be included for this outcome. Two were specific to omphalocele, 1 (Mellis et al. 2022) is a systematic review of diagnostic yield for WES in case of prenatally detected structural anomalies. **Shi. et al.** studied in 81 fetusses with omphalocele. All 81 first had CMA and Karyotyping. After CMA 25 were found abnormal (aneuploidy n=22), 56 were found normal. Only 3 patients accepted additional WES analysis, this resulted in 1 additional diagnosis of diastrophic dysplasia (DTD). WES was also performed to identify possible causal variants in three non-isolated omphalocele cases, and one pathogenic variant was successfullyidentified. In the study by **Que et al.** Among the 120 fetuses, 27 were diagnosed with isolated omphalocele and 93 with nonisolated omphalocele using prenatal ultrasonography. Cardiac anomalies were the most observed cause in 17 fetuses. Routine karyotyping and CMA were performed on 35 patients, and chromosomal abnormalities were observed in five patients; all were non-isolated omphalocele cases. Six non-isolated cases had normal CMA results and conventional karyotype tests, but further WES examination revealed one pathogenic variant and two suspected pathogenic variants. The systematic review by **Mellis et al.** reviewed 4350 fetusses with structural anomalies in which WES analysis was performed. Overall, WES had a pooled incremental yield of 31% (95% confidence interval (CI) 26%–36%,$p < 0.0001$). A subgroup analysis for abdominal wall defects was performed, but sample sizes of the were too small to reach statistical significance in estimates of pooled diagnostic yield. Mellis et al. conclude that there is a large variety of diagnostic yield between different structural anomalies and that the expected diagnostic yield depends on the body system(s) affected and can be optimised by pre-selection of cases following multi-disciplinary review to determine that a monogenic cause is likely.

**Possible undesirable effects to consider**

Whole exome sequencing (WES), Chromosomal Microarray (CMA) and karyotyping for genetic screening in fetuses with Omphalocele

Bibliography: Shi et al. 2021, Que et al. 2023, Mellis et al. 2022

Certainty assessment							Summary of findings
Expert consensus<br><br>Brioudé, 2018	-	-	-	-	-	**-**	Although chorionic villus (CVS) cells, amniotic fluid (AF) cells or foetal blood cells (native and cultured) might be used for molecular testing, it is possible that cell culture might influence the methylation patterns. In CVS cells, the methylation pattern at 11p15. might be different from that of embryonic tissues and/or CVS cells might not reflect the (epi)genetic constitution of the foetus, and therefore false positive results might occur. False-negative prenatal tests might occur with all types of testing due to mosaicism; therefore, a normal prenatal test result cannot absolutely exclude a diagnosis of BWS.

CI: confidence interval

Explanations

a. None of the included studies on abdominal wall defects had a fully paired or randomized design. Selection for WES analysis was based on willingness to participate only.

b. High between study heterogeneity was reported for the biggest study, with a I2 of 94%.

c. Stongest evidence comes from a systematic review that inlcudes all types of structural anomalies, but couldn’t draw any conclusions specific to abdominal wall defects

d. Testing for publication bias showed significant funnel plot asymmetry (z = 5.6, p < 0.0001 by Egger's test); indicating possible publication bias.

Conclusions

PICO	Outcome	Conclusion	GRADE

1	Diagnostic yield	There are cautious indications whole exome sequencing (WES) has a slightly higher diagnostic yield in fetuses with isolated or non-isolated omphalocele compared to karyotyping and chromosomal microarray. It is unclear what the diagnostic accuracy of WES is specific for Beckwith Widenmann Syndrome (BWS).	Very Low

Considerations

Poaty, H., Pelluard, F., Diallo, M.S. et al. Omphalocele: a review of common genetic etiologies. *Egypt J Med Hum Genet* 20, 37 (2019). https://doi.org/10.1186/s43042-019-0040-3	Review on the condition, insisting on the risk factors of omphaloceles mainly of those of genetic origins		Most commonly, chromosomal abnormalities are reported in omphaloceles (38-67%), mainly aneuploidies. Ranked first and second are trisomy 18 and 13. Other chromosomal aberrations that can be identified are for instance: triploidy; monosomy X (Turner syndrome); 47, XXY (Klinefelter syndrome); trisomy 16 and 21 (very low contributors); partial trisomies. Familial syndromal forms of omphalocele over two generations (requiring genetic counseling) are reported. The mode of inheritance can be autosomal dominant, autosomal recessive or X-linked trait. The most common syndrome is the Beckwith-Wiedemann syndrome (BWS), seen in 3 to 22% of omphaloceles.
Abbasi, Nimrah, et al. "Prenatally diagnosed omphaloceles: report of 92 cases and association with Beckwith-Wiedemann syndrome." Prenatal Diagnosis 41.7 (2021): 798-816.	Describe the prevalence, perinatal and long-term outcomes of Beckwith-Wiedemann syndrome (BWS) among prenatally detected omphaloceles.	Case series (n=92)	62 had additional anomalies. Abnormal karyotypes were identified in 23/62 (37%) non-isolated and 2/30 (7%) isolated cases. One BWS case (5%) was identified among non-isolated omphaloceles and six BWS cases (37.5%) were identified among isolated omphaloceles after exclusion of aneuploidy. MS-MLPA, was used to test for BWS. 5% of non-isolated with normal karyotype was tested positive for BWS 37.5% of isolated cases with normal karyotype was tested positive for BWS

			BWS should be considered in all prenatally diagnosed omphaloceles, particularly when in isolation and after exclusion of chromosomal abnormalities
Brioude, F., Kalish, J., Mussa, A. et al. Clinical and molecular diagnosis, screening and management of Beckwith–Wiedemann syndrome: an international consensus statement. *Nat Rev Endocrinol* 14, 229–249 (2018). https://doi.org/10.1038/nrendo.2017.166	Clinical and molecular diagnosis, screening and management of Beckwith–Wiedemann syndrome: an international consensus statement	Expert consensus statement following modified Delphi consensus protocol	Abnormal prenatal biochemical screening results — for example, elevated levels of free beta-human chorionic gonadotropin (hCG) in the first trimester and/or increased α-fetoprotein (αFP) levels in the second trimester (associated with exomphalos)— can be associated with BWS in the foetus. Also structural anomalies like omphalocele but also macrosomia, hemihypertrophy, organomegaly and polyhydramnios can raise suspicion for BWS. Prenatal molecular diagnostic investigations should be considered if prenatal ultrasonography reveals potential features of BWS and lead to a specific diagnosis (or exclude other potential conditions); or if positive family history with a known molecular defect is present, which would influence the management of the relevant pregnancy. Prior to offering prenatal diagnosis for BWS, a detailed discussion of the technological limitations and ethical issues should be undertaken with the parents; in particular, they should be made aware that a normal result does not necessarily exclude the diagnosis.<br><br>No recommendations on specific genetic testing were made as in prenatal testing the ideal screening method depends on the individual situation of the patient (for example known molecular defect and clinical features).

PICO 1.2: Should babies with omphalocele be delivered vaginally or via cesarean section?

Cesarean section compared to vaginal birth for delivery of babies with small omphalocele

Bibliography: How et al. 2000

Certainty assessment							Summary of findings		
Participants (studies) Follow-up	Risk of bias	Inconsistency	Indirectness	Imprecision	Publication bias	Overall certainty of evidence	Study event rates (%)		Impact
							With vaginal birth	With cesarean section	
**Neonatal mortality**									
32 (1 non-randomised study)	serious[a]	not serious	not serious	serious[b]	none	⊕◯◯◯ Very low	How et al. reported on a group of 32 prenatally diagnosed omphalocele patients. All fetuses had small omphaloceles with no liver herniation. 28 had elective cesarean section, 4 were delivered vaginally. There was no significant difference between the groups, neonatal mortality rate was 7% in the cesarean section group and 25% in the vaginal delivery group (p=0.2).		
**Lenght of hospital stay**									
32 (1 non-randomised study)	serious[a]	not serious	not serious	serious[b]	none	⊕◯◯◯ Very low	How et al. reported that the median length of stay was 39 days (IQR 25-56) for the cesarean delivery and 24 days (IQR 8-71) for the vaginal delivery (p=0.004, with Mann-Whitney test).		
**Time to full feeding**									
32 (1 non-randomised study)	serious[a]	not serious	not serious	serious[b]	none	⊕◯◯◯ Very low	How et al. reported that the median time to enteral feeding was 12 days (IQR 6, 15) for the cesarean group and 16 days (IQR 3, 29) for the vaginal delivery group (p=0.03 with Mann-Whitney test)		

CI: confidence interval

Explanations

a. Analyses are not corrected for possible confounders, data is collected over a spread period of time (10 years) in which interventions and management may have changed.

b. Very small sample in the vaginal delivery group

Conclusions

PICO	Outcome	Conclusion	GRADE
2	Neonatal mortality	There are cautious indications patients with omphalocele have comparable risks for neonatal mortality after vaginal and cesarean delivery	Very Low
2	Length of stay	There are cautious indications patients with omphalocele have a shorter length of stay after vaginal delivery.	Very Low
2	Time to full feeding	There are cautious indications patients with omphalocele have comparable time to feeding after vaginal and cesarean delivery	Very Low

PICO 1.3a : What is the prognostic value of prenatally diagnosed liver herniation for postnatal outcome

Question: Postnatal course of disease in omphalocele patients with prenatally diagnosed liver herniation

Bibliography: Connor, 2018; Chock, 2019; Montero, 2011; Nicholas, 2009; Hidaka, 2009

№ of studies	Certainty assessment						Effect			Certainty	Importance
	Study design	Risk of bias	Inconsistency	Indirectness	Imprecision	Other considerations	№ of events	№ of individuals	Rate (95% CI)		
Mortality											
2	non-randomised studies	serious[a]	not serious	not serious	serious[b]	none	5	24	event rate 0,20 per 1 (0.07 to 0.49)	⊕⊕◯◯ Low	CRITICAL
Adverse neonatal outcome (assessed with: 1 or more of the following outcomes present: death, prolonged hospital stay, feeding difficulties or sepsis)											
2	non-randomised studies	not serious	serious[c]	Serious[e]	serious[d]	none	24	28	event rate 0,86 per 1 (0.55 to 1.28)	⊕⊕◯◯ Low	IMPORTANT
Ability to perform primary repair											
1	non-randomised studies	Serious[f]	not serious	not serious	serious[d]	none	Montero et al. reported that 100% of 13 patients who had staged or delayed repair had extracorporeal liver.<br><br>75% of 12 patients who had primary repair had extracorporeal liver, the			⊕⊕◯◯ Low	IMPORTANT

№ of studies	Certainty assessment						Effect			Certainty	Importance
	Study design	Risk of bias	Inconsistency	Indirectness	Imprecision	Other considerations	№ of events	№ of individuals	Rate (95% CI)		
							difference was not significant. (p=0.10)<br><br>In the performance test for predicting inability to primary close the defect extracorporeal liver (n =22) had a sensitivity of 100.0 specificity of 25.0 PPV of 59.1 and NPV of 100.0 (AUC 0.63) for predicting inability to perform primary omphalocele repair.<br><br>Peters et al. reported that the omphalocele was closed primarily in all 32 infants without liver herniation, and 31 infants survived. Not having liver herniation, independent of the OC/AC ratio, was a perfect predictor for primary closure. 100% of 26 patients who had delayed repair had extracorporeal liver. Only 5% of 37 patients with primary repair had extracorporeal liver (p<0.001).				

Explanations

a. In all studies, only percentages of mortality incidence could be compared, while no correction for possible confounders was applied.

b. Both small series and zero indicents of death in the control group of Hidaka (2009)

c. Conflicting results. In one study extracorporeal liver was significantly associated with adverse outcome, this remained in multivariate analysis(Nicholas). In the other study (Chock) extracorporeal liver was not associated with adverse outcome(death or prolonged lenght of stay.

d. Small series

e. Combined outcome with severe and less severe events, different definition between studies

f. Possible selection bias due to referrals of more complicated cases to the center this study was conducted. Also, 16.4% of eligible patients was exlcuded for incomplete patient data. In the study by Peters et al.,Nine patients were lost to follow-up because they were born in another center, it is unknown if the characteristics of these patients would have influenced the prognostic factor.

Conclusions

PICO	Outcome	Conclusion	GRADE
3a	Mortality	There are indications prenatally detected extracorporeal liver is a predictor for a higher mortality rate compared to intracorporeal liver	Low
3a	Adverse neonatal outcome	There are indications prenatally detected extracorporeal liver is predictive for more neonatal adverse outcomes compared to intracorporeal liver	Low
3a	Ability to perform primary repair	There are indications prenatally detected extracorporeal liver is an independent predictor for the inability to perform primary repair.	Low

PICO 1.3b : What is the prognostic value of prenatally measured omphalocele ratio's for postnatal outcome **Question**:

Postnatal course of disease in omphalocele patients with prenatally measured omphalocele ratio's

Bibliography: Peters et al. 2019, Kleinrouweler et al. 2011, Chock et al. 2019, Nitsche et al.2021, Fawley et al.2016, Montero et al. 2011, Kiyohara et al. 2014, Tassin et al. 2013

№ of studies	Certainty assessment						Effect			Certainty	Importance
	Study design	Risk of bias	Inconsistency	Indirectness	Imprecision	Other considerations	№ of events	№ of individuals	Rate (95% CI)		
**Prognostic value of OC/AC for Mortality**											
1	non-randomised studies	serious[a]	not serious	not serious	not serious[b]	none	In a study by Peters et al. OC/AC ratio was measured at 3 point during gestation: US1: 11-16 weeks US2: 17-26 weeks US3: 30-38 weeks The OC/AC ratio could be calculated for 22 fetuses at US1, for 50 at US2, and for 58 at US3. Separate ROC analyses for each of the three- measurement time periods revealed a statistically significant negative association between the OC/AC ratio and survival at US2 and US3. At US2 an AUC of 0.81 (with a 95% CI of 0.61–1.00; p = .01), and at US3 an AUC of 0.89 (with a 95% CI of 0.79–0.98; p = .001).			⊕⊕⊕◯ Moderate	CRITICAL
**Prognostic value of OC/AC for inability of primary repair**											
1	non-randomised studies	serious[c]	not serious	not serious	not serious[b]	none	Kleinrouweler et al. (2011) reported over 13 cases that all cases with a primary reconstruction had OC/AC ratios below 0.57, all cases with staged closure had higher OC/AC ratios above			⊕⊕⊕◯ Moderate	IMPORTANT

№ of studies	Certainty assessment						Effect			Certainty	Importance
	Study design	Risk of bias	Inconsistency	Indirectness	Imprecision	Other considerations	№ of events	№ of individuals	Rate (95% CI)		
							0.75. Thus, any cutoff between 0.57 and 0.75 has 100% sensitivity and specificity.<br><br>Peters et al. reported that the OC/AC ratio was significantly positively associated with the probability of requiring a delayed closure at all three measured time periods. Based on ROC curve analysis, the type of closure was predicted correctly by the OC/AC ratio with optimal cut-off values of 0.69 at US1 (sensitivity 0.93 and specificity 0.90; AUC 0.96, 0.88–1.00; p < .001), 0.66 at US2 (sensitivity 0.88 and specificity 0.93; AUC 0.98, 0.95–1.00; p < .001), and 0.63 at US3 (sensitivity 0.95 and specificity 0.94; AUC 0.98, 0.95–1.00; p < .001)				
Prognostic value of OC/AC for respiratory insuficciency (assessed with: need of ventilation for >24 hours)											
1	non-randomised studies	serious[c]	not serious	not serious	not serious[b]	none	Kleinrouweler et al. (2011) reported over 15 cases, there was a trend towards more respiratory insufficiency for cases with higher OC/AC ratio: for a 0.10-point increase in the OC/AC ratio, the OR for respiratory insufficiency was 1.59 (95% CI = 0.95–2.67).<br><br>The optimal cut-off for predicting respiratory insufficiency was found to			⨁⨁⨁◯ Moderate	IMPORTANT

№ of studies	Certainty assessment						Effect			Certainty	Importance
	Study design	Risk of bias	Inconsistency	Indirectness	Imprecision	Other considerations	№ of events	№ of individuals	Rate (95% CI)		
							be 0.66 with a sensitivity of 67%, a specificity of 78% and an AUC of 0.76.				
**Prognostic value of OD/AC for mortality**											
2	non-randomised studies	serious[c]	serious[d]	not serious	not serious[b]	none	Fawley et al. looked at OD/AC ratios in 30 patients. The optimal cut-off was determined 0.26. There was no difference in mortality between patients with a cutoff below (1/20) or above (1/10) the optimal cut-off (p=1). However, a study by Kiyohara et al. looked at OD/AC ratio's in 47 patients with the same cut-off of 0.26. In this study, a OD/AC ratio above 0.26 in the first scan (was associated with a higher likelyhood of neonatal death (Likelyhood ratio 4, 95% CI 1.9-7.5,)			⊕⊕◯◯ Low	CRITICAL
**Prognostic value of OD/AC for adverse outcomes (assessed with: death before discharge or prolonged length of stay (LOS))**											
1	non-randomised studies	not serious	not serious	serious[e]	serious[bg]	none	Chock et al. assessed the AWD/AC ratio of patients in this cohort was compared against a threshold of 0.24 (defined by Montero et al. 2011). AWD/AC >0.24 was associated with adverse outcome (p=0.02). However, after correction for confounders associated with death or lenght of stay, the association was not significant anymore (OR 2.8 95%CI 0.01–520, p=0.70).			⊕⊕◯◯ Low	IMPORTANT

№ of studies	Certainty assessment						Effect			Certainty	Importance
	Study design	Risk of bias	Inconsistency	Indirectness	Imprecision	Other considerations	№ of events	№ of individuals	Rate (95% CI)		
Prognostic value of OD/AC for inability of primary repair											
4	non-randomised studies	serious[c]	not serious	not serious	not serious	none	In the study by Montero et al. (2011) (n=24) the OD/AC ratio was predictive for the inability to primary closure with a cut-off of >/=0.24 (OR 7, 95%CI 1.1-41.3). The Sensitivity was 83.3, Specificity was 58.3, PPV 66.7 and NPV 77.8 with an AUC of 0.71. Nitsche et. al. found in their series of 17 patients that OD/AC ratio measured at the **maximum** omphalocele diameter with a optimal cut-off of >/=0.24 has 100% specificity to predict inability of primary repair. Kiyohara et al. (n=35) found that OD/AC ratio with a cut-off of >/=0.26, is predictive for the need of a two step surgery or the use of mesh (p<0.001). Fawley et al. used an optimal cut-off of 0.26. Sixty percent (12/20) in the low-ratio group achieved primary closure versus zero (0/10) in the high-ratio group (p = 0.001). All four studies conclude that OD/AC ratio's above 0.24-0.26 are predictive for inability of primary repair.			⨁⨁⨁◯ Moderate	IMPORTANT
Prognostic value of OD/AC for need of intubation/mechanical ventilation											
2	non-randomised studies	serious[c]	not serious	not serious	serious[f]	none	Fawley et al. (n=30) report that patients with a OD/AC ratio above 0.26 had significantly longer ventilation times (15.8 days (low-ratio) versus 79			⨁⨁◯◯ Low	IMPORTANT

№ of studies	Certainty assessment						Effect			Certainty	Importance
	Study design	Risk of bias	Inconsistency	Indirectness	Imprecision	Other considerations	№ of events	№ of individuals	Rate (95% CI)		
							days (high-ratio) (p = 0.05)). In the study of Kiyohara et al. more patients with a ratio above 0.26 needed intubation in the first 24h of life (4/21 (19%) vs. 8/14 (57.1%) , p=0.03). A ratio above 0.26 seemed also predictive for prolonged ventilation (>21 days) but due to low incidence within the sample, this could not be proven significant.				
Prognostic value of MOD/TAD for morbidity											
1	non-randomised studies	Not serious	not serious	not serious	Serious[b]	none	Tassin et al. looked at the mean **omphalocele** diameter/transvers abdominal diameter ratio (OD/TAD) in 54 fetuses with omphalocele during the first trimester <14 weeks gestation. They found a significant increase in morbidity (hospitalization in the intensive care unit for more than 42 days, need for respiratory assistance (mechanical ventilation, intubation) for more than 21 days and/or need for parenteral feeding for more than 21 days) in patients with a MOD/TAD ratio >0.8.			⨁⨁⨁◯ Moderate[b]	IMPORTANT
Prognostic value of MOD/TAD for mortality											

Nº of studies	Certainty assessment						Effect			Certainty	Importance
	Study design	Risk of bias	Inconsistency	Indirectness	Imprecision	Other considerations	Nº of events	Nº of individuals	Rate (95% CI)		
1	non-randomised studies	Not serious	not serious	not serious	Serious[b]	none	Tassin et al. Assessed the correlation between MOD/TAD during the first trimester and mortality. There were 4 deaths between 8 days and 8 months postpartum, mainly due to surgical infection or pulmonary hypoplasia. The study had insignificant statistical power to prove a significant correlation between the ratio and mortality, but all neonatal death cases had MOD/TAD >0.8			⨁⨁⨁◯ Moderate[b]	IMPORTANT

Explanations

a. Analysis was not corrected for important confounders such as congenital lung disease or other concomittent factors. For Peters et al: Nine patients were lost to follow-up because they were born in another center, it is unknown if the characteristics of these patients would have influenced the prognostic factor.

b. Small sample size.

c. Possible attrition bias: unclear if patients with images available/included patients had different characteristics than patients eligible without available images. Possible bias due to confounding as only univariate analysis was performed

d. Studies have conflicting outcomes as to predictive value for mortality. This may be inconsistency but may also be due to a statistical power issue in the study of Fawley.

e. Combined outcome measurement

f. Small sample size and low incidence of sought effect, possibly lack of statistical power in the analysis of Kiyohara

g. Very wide confidence interval on the multivariate analysis

Definitions

OC/AC: Omphalocele circumference / abdominal circumference

OD/AC: Omphalocele diameter / abdominal circumference

OD/TAD → Omphalocele diameter / transverse abdominal diameter

Conclusions

PICO	Outcome	Conclusion	GRADE
3b	Mortality	It is likely there is a significant negative association between the **OC/AC** ratio (measured between 17 and 38 weeks gestation) and survival	Moderate
3b	Ability to perform primary repair	It is likely that an **OC/AC** ratio is significantly associated with the probability of requiring delayed or staged closure. The optimal cut-off probably varies with gestational age.	Moderate
3b	Respiratory insufficiency	It is likely that there is a trend towards more respiratory insufficiency in patients with higher **OC/AC** ratios.	Moderate
3b	Mortality	There are indications that **OD/AC** ratios >0.26 are associated with a higher likelyhood of death.	Low
3b.	Adverse outcomes	It is likely OD/AC ratio is not associated with the probability of one or more adverse outcomes (death before discharge or prolonged LoS)	Low

3b	Ability to perform primary repair	It is likely that an **OD/AC** ratio above 0.24-0.26 is predictive for inability of primary repair of the omphalocele.	Moderate
3b	Respiratory problems	There are indications that an **OD/AC** ratio above 0.26 is predictive for intubation in the first 24 hours of life and longer need for mechanical ventilation.	Low
3b	Morbidity	It is likely that MOD/TAD >0.8 measured in the first trimester (<14 weeks gestation) is predictive for neonatal morbidity	Moderate

PICO 3.1: Staged surgical reduction compared to conservative management (paint and/or wait) for patients with giant omphalocele

Bibliography: Bauman, 2016; Binet, 2020; Menchaca, 2023

Certainty assessment							Summary of findings		
							Study event rates (%)		
Participants (studies) Follow-up	Risk of bias	Inconsistency	Indirectness	Imprecision	Publication bias	Overall certainty of evidence	With conservative management (paint and/or wait)	With staged surgical reduction	Impact
**Mortality**									
(3 non-randomised studies)	serious[a]	not serious	not serious	serious[b]	none	⊕◯◯◯ Very low[a,b]	Three studies were included for this outcome. Bauman et al. 2016 systematically reviewed studies reporting on either staged surgical or delayed conservative management. 15/64 patients with staged reduction (23.4%) died versus 62/286 (21.8%) in the delayed group. The relative risk for mortality was 1.08 (95%CI 0.65-1.77) and was not siginificant (p=0.08). After this systematic review, two observational studies were published comparing staged and delayed reduction. Binet et al. 2020 reported a lower mortality rate for staged reduction, but the difference was not significant (RR 0.48, 95%CI 0.21-1.09, p=0.08). Menchaca et al. 2023 reported a lower mortality rate in the delayed group (0%) but also here, there was no significant difference with the staged group. (RR 6.0, 95%CI 0.26-136.53, p=0.26).		

Lenght of Stay (assessed with: Number of days spend in the hospital at primary admission)

PICO 3.1: Staged surgical reduction compared to conservative management (paint and/or wait) for patients with giant omphalocele

Bibliography: Bauman, 2016; Binet, 2020; Menchaca, 2023

Certainty assessment							Summary of findings
(3 non-randomised studies)	serious[c]	not serious	not serious	not serious	none	⊕◯◯◯ Very low[c]	Three studies were included for this outcome. Bauman et al. 2016 and Menchaca et al. 2023 both reported that there was no significant difference in LoS between the staged and delayed patients (p=0.07 and p=0.221). However, Binet et al. reported a large difference in lenght of stay between staged and delayed closure (mean 95 days[range 49-174] vs. mean 11 days [range 1-60], p<0.01).

Time to full feeds (assessed with: Number of days untill full enteral feeding)

(2 non-randomised studies)	serious[d]	not serious	not serious	not serious	none	⊕◯◯◯ Very low[d]	Two studies were included for this outcome. Bauman et al. 2016 reported a significant shorter time to full feeding for patients with giant omphalocele in the dealyed group (mean 23.5 days vs. 14.6 days, p= 0.0018). Menchaca et al. 2023 reported shorter times to full feeding in the delayed group as well, but their difference wasn't significant (median 39 days vs. 28 days, p=0.6)

CI: confidence interval

Explanations

a. In none of the studies, outcomes were corrected for confounders. The study of Binet et al., additionally may carry bias by study site, as all patients in the staged group were treated in a high resource hospital in France and all patients in the delayed group were treated in a lower resource hospital in Ivory coast, without a neonatal unit and a lack of technical means for resuscitation and anesthesia.

b. Very wide confidence interval and low number of incidents in the study by Menchaca et al. 2023

c. In none of the studies, outcomes were corrected for confounders. The study of Binet et al., additionally may carry bias by study site, as all patients in the staged group were treated in a high resource hospital in France and all patients in the delayed group were treated in a lower resource hospital in Ivory coast, without a neonatal unit and a lack of technical means for resuscitation and anesthesia. This may have biassed the results for lenghts of stay as the more 'complicated' cases are alive and longer in hospital in France, while they already died in Ivory coast and therefore don't have longer hospital stays. The study of Bauman et al. reports possible selection bias influencing lenght of stay outcomes as there were more patients with severe comorbidities in the delayed group.

d. In none of the studies, outcomes were corrected for confounders.

Conclusions

PICO	Outcome	Conclusion	GRADE
7	Mortality	There are some indications the risk for mortality is nog significantly different after staged or delayed treatment for giant omphalocele.	Very Low
7	Length of Stay	There are some indications there is no significant difference in length of stay after staged or delayed treatment for giant omphalocele.	Very Low
7	Time to full feeding	There are some indications that delayed closure may results in shorter times to full enteral feeding compared to staged surgical closure.	Very Low

PICO 3.2: Choice of substances for conservative management of giant omphalocele

Bibliography: Goneidy and Saxena (2023)

Certainty assessment							Summary of findings		
							Study event rates (%)		
Participants (studies) Follow-up	Risk of bias	Inconsistency	Indirectness	Imprecision	Publication bias	Overall certainty of evidence	With other substances	With a specific substance	Impact
**Time to full epithelisation**									
(1 non-randomised study)	very serious[a]	serious[b]	not serious	not serious	none	⊕◯◯◯ Very low[a,b]	For PICO 8 only 1 systematic review could be included. Goneidy and Saxena systematically reviewed results for different kinds of substances. Substances reported on were Honey (53 patients), 2% aquous eosin (271 patients), Gentian violet (47 patients), silver dressings/solution (136 patients), povidone iodine (98 patients), mercurochrome (91 patients), Saline (18 patients), dry dressing only (75 patients) and mixed agents (42 patients). The median time to full epithelisation was 64 days (30-120). The shortest median time to full epithelisation (30/31 days) was seen in a study from Eltayeb (2015) where they uesed Nilotica Powder + Gentian violet in half of the patients and Povidone Iodine in the other half. However, in another study (Whitehouse 2010, n=6) diluted povidone iodine was used which resulted in a median time to epithelisation of 120 days (90-180). Interestingly, less agressesive agents like honey and saline, seem to be on the shorter end of epithelisation days as well (Honey mean 34.4 and 2.5% Saline 45.7).		
**Toxicity**									

PICO 3.2: Choice of substances for conservative management of giant omphalocele

Bibliography: Goneidy and Saxena (2023)

Certainty assessment							Summary of findings
(1 non-randomised study)	very serious[a]	serious[b]	not serious	not serious	none	⊕◯◯◯ Very low[a,b]	For some agents, toxicity with longer epithelisation times is known. In this systematic review, toxicity was reported for alcohol, silver, mercury and iodine. Thyroid disfunction with iodine painting, as well as alcohol, silver and mercury toxicity have been reported. Goneidy and Saxena report that they found notions of toxicity mainly in older studies and declare that this could have to do with the higher concentrations of the applications back in the days. More recent studies report using diluted applications and show the agents' blood level to be at the upper end of normal, without any associated complications. Three studies of those more recent studies (Saxena 2018, Dörterler 2019, Bode 2018) describe the use of less toxic agents like honey and saline to avoid toxicity risk and with similar outcomes to the well performing toxic substances.

Complications

(1 non-randomised study)	very serious[a]	serious[b]	not serious	not serious	none	⊕◯◯◯ Very low[a,b]	In 20% of reviewd cases complications were reported (122/617 patients). Mortality was reported to be 24.5% (170/694) but mainly due to associated anomalies. Besides toxicities and hypothyroidism, sac rupure, infection, intestinal stricture and intestinal obstruction were reported. These happend in toxic as well as less/non-toxic agents.

CI: confidence interval

Explanations

a. Many studies included in the review are of poor quality. There are multiple confounding factors that are not accounted for, outcomes were poorly reported, and application and use of the agents was heterogenic between studies.

b. There are many discrepancies between studies, one agent can be on the better side of performance in one study and on the worse end in another.

Conclusions

PICO	Outcome	Conclusion	GRADE
8	Time to epithelization	There is no evidence to indicate superiority of one agent over another.	Very Low
8	Toxicity	There are some indications that less/non-toxic agents like honey and saline have similar performance to well performing toxic agents while decreasing toxicity risk.	Very Low
8	Complications	There is no evidence to indicate superiority of one agent over another.	Very Low

PICO 3.3 : *What are the (un)desirable effects of different methods for staged reduction and closure of the abdominal wall in patients with giant omphalocele?*

	Outcomes							Patient characteristics	Quality of evidence
Method	Time on ventilator	Circulation	Feeding	Time to full closure (days)	Length of stay (days)	Infection<br>Sepsis	Nursing		
Surgical silo<br>(2 studies, 61 patients)<br>Pacilli et al. 2005<br>Binet et al. 2020	Median 8 (2-20) days (n=9) in the study by Pacilli and<br>A mean of 20 (2-63) days (n=49) in the study by Binet.	NR	Median (range) 12 (4-53) (n=9) in the study by Pacilli and a mean of 175 (9—455) days (n=49) in the study by Binet.	Median 26 (16-62) days (n=9) in the study by Pacilli and a mean of<br>104 (2-174) days (n=49) in the study by Binet.	Median<br>42(23-72) days (n=9) in the study by Pacilli and a mean of 104 days 95 [49—174] days (n=49)	Mild inflammation of silo edges in 2 patients (n=9)<br>Clavien Dindo II complications in 19 patients (39%)<br>(n=49)	NR	In the series by Pacilli, no syndromes were reported but 9/12 original patients had associated anomalies. 3 patients died from respiratory or cardiac complications prior to measuring of the end points. In the cohort by Binet, 84% of patients were non-syndromic.	Level 4 studies with no correction for confounders

Non-surgical silo (Duoderm) (2 studies, 70 patients)<br><br>Abello et al. 2021<br>Barrios-Sanjuanelo et al. 2021	NR	NR	NR	Median 12 (10–16) days (n=40) in the study by Abello et al. and a median of 12 (10–17) days (n=30) reported by Barrios-Sanjuanelo	NR	Sepsis occurred in 17 patients in the cohort by Barrios-Sanjuanelo (56.7%) and in 17 patients of the cohort by Abello et al (42.5%)	NR	In the study by Abello at al. 37% had associated anomalies, of which 20% cardiac, 20% pulmonary hypertension and 5% pentology of catrell. Barrios-Sanjuanelo reported malformations in 43.3 %, Congenital heart defects in 26.7% Pulmonary hypertension 13.3% and Pentalogy of Cantrell in 6.7%	Level 4 studies with no correction for confounders
Barlow external silo (1 study, 14 patients)<br><br>Mitanchez et al. 2010	Median 26 days (range, 2-78).<br><br>longer for those infants who required<br><br>ventilatory support in the delivery room	NR	On average, enteral feeds were started 3 days after<br><br>abdominal wall closure (range, 2-8); and full	Median age at closure was 6 days (0-20). A central Gore-Tex patch was inserted in 10 cases.	Median time until discharge from the **intensive care unit** was 24.5 days (11-85)	4 cases of cathether related sepsis and 4 would infections	NR	10/14 patients had at least 1 associated anomaly.<br><br>1 patient with CDH, 4 with congenital heart disease, 4 with intestinal malrotation and 1 with	Level 4 study with no correction for confounders

	(median, 31.5 days; range, 13-63) compared to those intubated in the NICU (median, 10 days; range, 2-78)		enteral diet was achieved after an average of 33 days (8-82)					hirschsprung's disease.	
Patch sutured over the omphalocele (1 study, 39 patients) Saxena et al. 2002	Average of 3.2 days	NR	Average of 6.4 days on parenteral nutrition	At the age of 2 years a second operation was performed in which the scar tissue left behind by the dura patches was removed, accompanied by an abdominal wall plasty.	Average of 47.5 days	2 cases of catheter related sepsis	NR	50% of the sample had associated anomalies, the majority of this were cardiac anomalies	Level 4 study with no correction for confounders
Fasciotens (1 study, 10 patients)	Median of 12 (0–46) days	NR	NR	7 days (range 4–22) days	Median of 31 (17–137) days	No cases of sepsis or surgical site infection	NR	Patients with abdominal wall aplasia, coagulation disorders, and	Level 3 study with no correction for confounders

Ziegler et al. 2024						but sutures tore out in two cases.		cardiorespiratory instability including pulmonary hypertension, pulmonary hypoplasia, and severe cardiac defects were excluded	
Traction (1 study, 12 patients) Uecker et al. 2020	Mean 11 ± 19 days	NR	Mean 29 ± 26 days	Mean 7 ± 10 days	Mean 63 ± 53 days	NR	NR	4/10 patients had major comorbidities (CDH, pulmonary hypoplasia, pentalogy of Cantrell, tetralogy of Fallot)	Level 4 study with no correction for confounders
Taping (1 study, 10 patients) Kogut et al. 2018	NR	NR	NR	Mean 13.7 days	Mean 71.8 days	NR	NR	Four of the babies had documented chromosomal anomalies. Seven of the ten babies had other significant anomalies, and three had either no other findings	Level 4 study with no correction for confounders

								or only minor findings	
Suspension<br><br>(1 study, 10 patients patients)<br><br>Huang et al. 2020	Median of 22.2 days (range, 1–151)	NR	Median age at full enteral feeding 33.1 days (range,<br><br>13–66).	Median 8 days (4-18)	Median 44.2 days (range, 2–152)	5 patients had pneumonia , 3 were ventilator associated.	NR		Level 4 study with no correction for confounders

*inclusion criteria : ≥5 patients, publication year >2000, separate results on the intervention of interest
