# Supplementary material for: ERNICA evidence based guideline on omphalocele
Source: Orphanet J Rare Dis. 2026 Mar 7;21:193. doi: 10.1186/s13023-026-04293-7 (PMC13162432; doi:10.1186/s13023-026-04293-7)
Supplement: Supplementary file 3 — Supplementary Material 3 [file 13023_2026_4293_MOESM3_ESM.pdf]

S3. Evidence to Decision tables

QUESTION 1.1

**Should additional Whole Exome Sequencing (WES) vs. Chromosomal Microarray (CMA) / Karyotyping only be used for genetic screening in fetuses with omphalocele?**	
**POPULATION:**	Genetic screening in fetuses with omphalocele
**INTERVENTION:**	Additional Whole Exome Sequencing (WES)
**COMPARISON:**	Chromosomal Microarray (CMA) / Karyotyping only
**MAIN OUTCOMES:**	Diagnostic yield

ASSESSMENT

Desirable Effects<br>How substantial are the desirable anticipated effects?		
JUDGEMENT	RESEARCH EVIDENCE	ADDITIONAL CONSIDERATIONS
○ Trivial<br>● Small<br>○ Moderate<br>○ Large<br>○ Varies<br>○ Don't know	Three studies could be included for this outcome. Two were specific to omphalocele, one (Mellis et al. 2022) is a systematic review of diagnostic yield for WES in case of prenatally detected structural anomalies. **Shi. et al.** studied in 81 fetuses with omphalocele. All 81 first had CMA and Karyotyping. After CMA, 25 were found abnormal (aneuploidy n=22), 56 were found normal. Only 3 patients accepted additional WES analysis; this resulted in 1 additional diagnosis of diastrophic dysplasia (DTD). WES was also performed to identify possible causal variants in three non-isolated omphalocele cases, and one pathogenic variant was successfully identified. In the study by **Que et al.,** among the 120 fetuses, 27 were diagnosed with isolated omphalocele and 93 with non-isolated omphalocele using prenatal ultrasonography. Cardiac anomalies were the most	The panel concluded that additional prenatal WES testing has probably small benefits. Their rationale for offering it as a prenatal test is that the turnover time of WES is a few weeks, which could be a long time if the patient is already born, and management of the patient turns out to be challenging. Despite the low certainty of evidence, most panel members agree that WES has probably an additional diagnostic yield which could be beneficial for both parents and caregivers. Identification of a syndrome with a big disease burden has potential benefits. It can help parents to make a decision about termination of the pregnancy but can also help parents and healthcare providers to optimally prepare for what is ahead. A specific diagnosis may also give information about the risk in future pregnancies. Thereby, qualitative

	observed cause in 17 fetuses. Routine karyotyping and CMA were performed on 35 patients, and chromosomal abnormalities were observed in five patients; all were non-isolated omphalocele cases. Six non-isolated cases had normal CMA results and conventional karyotype tests, but further WES examination revealed one pathogenic variant and two suspected pathogenic variants. The systematic review by **Mellis et al.** reviewed 4350 fetuses with structural anomalies in which WES analysis was performed. Overall, WES had a pooled incremental yield of 31% (95% confidence interval (CI) 26%–36%,p < 0.0001). A subgroup analysis for abdominal wall defects was performed, but sample sizes were too small to reach statistical significance in estimates of pooled diagnostic yield. Mellis et al. conclude that there is a large variety of diagnostic yield between different structural anomalies and that the expected diagnostic yield depends on the body system(s) affected and can be optimized by pre-selection of cases following multi-disciplinary review to determine that a monogenic cause is likely.<br><br>The panel concludes that an additional WES analysis probably has small benefits.	research indicates that it is helpful for parents to have a specific diagnosis (Bauskis et al. 2022).
**Undesirable Effects**<br>How substantial are the undesirable anticipated effects?		
JUDGEMENT	RESEARCH EVIDENCE	ADDITIONAL CONSIDERATIONS
● Trivial<br>○ Small<br>○ Moderate<br>○ Large<br>○ Varies<br>○ Don't know	Although chorionic villus (CVS) cells, amniotic fluid (AF) cells or fetal blood cells (native and cultured) might be used for molecular testing, it is possible that cell culture might influence the methylation patterns. In CVS cells, the methylation pattern at 11p15 might be different from that of embryonic tissues and/or CVS cells might not reflect the (epi)genetic constitution of the fetus, and therefore false positive results might occur. False-negative prenatal tests might occur with all types of testing due to mosaicism; therefore, a normal prenatal test	Trivial harms are induced by the test itself - no additional material has to be retrieved and the test can be completed with the same sample as was used for karyotyping/CMA. With WES, there is a risk of incidental findings, such as carrier status for serious diseases in parents. Aside from the risk of false negatives, as described by Brioude (1) false negatives can also be induced by national policies. The type of results a provider is allowed to give to patients differs per country. In some countries, like France, it is only allowed to give class 4 or 5 results (confirmed pathogenic) to patients/parents. The panel

	result cannot absolutely exclude a diagnosis of Beckwith-Wiedemann Syndrome (BWS) (Brioude, 2018).	considered that there are trivial harmful effects if parents are well informed pre-test and provide their consent.

Certainty of evidence

What is the overall certainty of the evidence of effects?

JUDGEMENT	RESEARCH EVIDENCE	ADDITIONAL CONSIDERATIONS
● Very low<br>○ Low<br>○ Moderate<br>○ High<br>○ No included studies	The certainty of evidence was rated as 'very low' since all evidence came from observational studies, since there was high between-study heterogeneity in the included systematic review as well as indications for publication bias. Furthermore, the systematic review by Mellis et al. (2022) included all types of structural congenital anomalies but couldn't draw any conclusions specific to abdominal wall defects due to the low sample size.	

Values

Is there important uncertainty about or variability in how much people value the main outcomes?

JUDGEMENT	RESEARCH EVIDENCE	ADDITIONAL CONSIDERATIONS
○ Important uncertainty or variability<br>● Possibly important uncertainty or variability<br>○ Probably no		Panel members agreed that the value parents and clinicians attach to additional diagnoses probably depends on country, religion and inter-person differences, but is strongly linked to pre-test counselling. A qualitative analysis by Bauskis et al. (2022) underlines that genetic information is necessary for possible subsequent pregnancies and for the siblings' future children.

important uncertainty or variability<br>O No important uncertainty or variability		

Balance of effects

Does the balance between desirable and undesirable effects favor the intervention or the comparison?

JUDGEMENT	RESEARCH EVIDENCE	ADDITIONAL CONSIDERATIONS
O Favors the comparison<br>O Probably favors the comparison<br>O Does not favor either the intervention or the comparison<br>● Probably favors the intervention<br>O Favors the intervention<br>O Varies<br>O Don't know		Additional information on the condition of the patient will provide parents with more facts to base their decision on if they are considering pregnancy termination. If parents are not considering pregnancy termination, additional information may help parents as well as clinicians to optimally prepare for caring for this child. Thereby, qualitative evidence suggests that having a diagnosis is experienced as beneficial by parents. Considering the same sample as was used for karyotyping/CMA can be used for WES, the panel considered the possible undesirable effect such as false negatives or incidental findings trivial, if parents are fully informed prior to testing and informed consent is obtained. Therefore, the panel decided that the balance of effects probably favors the intervention (WES in addition to karyotyping/CMA).

Resources required

JUDGEMENT	RESEARCH EVIDENCE	ADDITIONAL CONSIDERATIONS
○ Large costs<br>● Moderate costs<br>○ Negligible costs and savings<br>○ Moderate savings<br>○ Large savings<br>○ Varies<br>○ Don't know		There are considerable additional costs that come with a WES analysis on a patient-specific level. However, overall omphalocele is still a very rare condition so the increase of costs per patient is only in a very limited number of patients. Thereby, the samples that are necessary to perform WES analysis are already available as the test can be done with the same sample that was used for karyotyping/CMA. Rapid developments in genetic care must be considered as this means that the costs of these tests are rapidly decreasing and will probably still decrease over the next few years. Costs aside, WES testing is not available in every hospital yet and additional training and resources are necessary for teams before starting to do this.

Certainty of evidence of required resources

What is the certainty of the evidence of resource requirements (costs)?

JUDGEMENT	RESEARCH EVIDENCE	ADDITIONAL CONSIDERATIONS
○ Very low<br>○ Low<br>○ Moderate<br>○ High<br>● No included studies		

Cost effectiveness

Does the cost-effectiveness of the intervention favor the intervention or the comparison?

JUDGEMENT	RESEARCH EVIDENCE	ADDITIONAL CONSIDERATIONS
O Favors the comparison<br>O Probably favors the comparison<br>O Does not favor either the intervention or the comparison<br>O Probably favors the intervention<br>O Favors the intervention<br>O Varies<br>● No included studies		

Equity

What would be the impact on health equity?

JUDGEMENT	RESEARCH EVIDENCE	ADDITIONAL CONSIDERATIONS
O Reduced<br>O Probably reduced<br>O Probably no impact<br>O Probably increased<br>O Increased<br>O Varies<br>● Don't know		The panel discussed two important considerations for health equity. On the one hand, if WES may not be widely available yet, centers may have contracts with other centers in other countries and be able to send off their samples for analysis. However, if not covered by insurance, a recommendation in favor of the intervention could possibly decrease equity. However, if WES is recommended as a standard of care, patients may be more likely to have the possibility to obtain a more accurate diagnosis which could help increase equal chances for the best possible care in the best possible place.

Acceptability
Is the intervention acceptable to key stakeholders?

JUDGEMENT	RESEARCH EVIDENCE	ADDITIONAL CONSIDERATIONS
O No<br>O Probably no<br>● Probably yes<br>O Yes<br>O Varies<br>O Don't know		A clinician/parent's religious views may influence how acceptable the intervention is perceived to be. Legal and financial barriers may also play a role.<br><br>Parents may not wish to know if they are disease carriers.<br><br>The panel members agreed that a recommendation in favor of WES testing is probably acceptable. However, pre-test genetic counselling should be provided, and patient choice should be considered and accounted for.

Feasibility
Is the intervention feasible to implement?

JUDGEMENT	RESEARCH EVIDENCE	ADDITIONAL CONSIDERATIONS
O No<br>O Probably no<br>● Probably yes<br>O Yes<br>O Varies<br>O Don't know		The panel members agreed that a recommendation in favor of WES is probably feasible (and sustainable) as the additional costs will reduce over time. Technically WES is not yet available everywhere, which could introduce the need for additional training and facilities for some teams. Until then, smaller centers/countries can outsource their tests to other hospitals within ERNICA. ERNICA can support the setup of such outsourcing agreements.

SUMMARY OF JUDGEMENTS

	JUDGEMENT						
**PROBLEM**	No	Probably no	Probably yes	Yes		Varies	Don't know
**DESIRABLE EFFECTS**	Trivial	**Small**	Moderate	Large		Varies	Don't know

	JUDGEMENT						
UNDESIRABLE EFFECTS	**Trivial**	Small	Moderate	Large		Varies	Don't know
CERTAINTY OF EVIDENCE	**Very low**	Low	Moderate	High			No included studies
VALUES	Important uncertainty or variability	**Possibly important uncertainty or variability**	Probably no important uncertainty or variability	No important uncertainty or variability			
BALANCE OF EFFECTS	Favors the comparison	Probably favors the comparison	Does not favor either the intervention or the comparison	**Probably favors the intervention**	Favors the intervention	Varies	Don't know
RESOURCES REQUIRED	Large costs	**Moderate costs**	Negligible costs and savings	Moderate savings	Large savings	Varies	Don't know
CERTAINTY OF EVIDENCE OF REQUIRED RESOURCES	Very low	Low	Moderate	High			**No included studies**
COST EFFECTIVENESS	Favors the comparison	Probably favors the comparison	Does not favor either the intervention or the comparison	Probably favors the intervention	Favors the intervention	Varies	**No included studies**
EQUITY	Reduced	Probably reduced	Probably no impact	Probably increased	Increased	Varies	**Don't know**
ACCEPTABILITY	No	Probably no	**Probably yes**	Yes		Varies	Don't know
FEASIBILITY	No	Probably no	**Probably yes**	Yes		Varies	Don't know

TYPE OF RECOMMENDATION

Strong recommendation against the intervention	Conditional recommendation against the intervention	Conditional recommendation for either the intervention or the comparison	**Conditional recommendation for the intervention**	Strong recommendation for the intervention
○	○	○	●	○

CONCLUSIONS

Recommendation

The panel suggests offering Whole Exome Sequencing (WES) to all omphalocele patients (and when available, Whole Genome Sequencing [WGS]).

Good practice statement:
Parents should receive adequate pre-test counselling explaining the possible benefits/harms, tailored to the local situation. Parents' choice should be accounted for by obtaining informed consent.

Justification

Balance of effects

Additional information on the patient's condition will provide parents with more facts to base their decision on if they are considering pregnancy termination. If parents are not considering pregnancy termination, additional information may help parents as well as clinicians to optimally prepare for caring for this child. Considering that the same sample used for karyotyping/CMA can be used for WES, the panel considered the possible undesirable effect such as false negatives or incidental findings to be trivial, if parents are fully informed prior to testing and consent is obtained. Therefore, the panel decided that the balance of effects probably favors the intervention (WES in addition to Karyotyping/CMA).

Subgroup considerations

The panel found no evidence that the recommendation should be different for different subgroups such as small or giant omphalocele.

Implementation considerations

To gain insight into current practices pertaining to the management of omphalocele in Europe, a baseline survey has been conducted amongst centers involved in the European Reference Network for rare Inherited Congenital Anomalies (ERNICA) and/or connected to the European Pediatric Surgical Audit (EPSA). Center representatives are asked whether their center offers prenatal genetic screening, as well as whether they offer WES (in addition to Karyotyping/Chromosomal Microarray). They have the opportunity to provide additional explanation.

A qualitative study will also take place to further explore the factors foreseen to hinder/facilitate successful implementation of this recommendation in clinical practice. This will be done with a view to collaboratively selecting implementation strategies.

The panel noted that to provide WES, teams will require appropriate training. ERNICA may play a role in facilitating and supporting such training initiatives. Outsourcing samples for analysis in different countries was also noted by the panel as a possible way to arrange WES testing in centers without the necessary resources. As a European expertise network, ERNICA can also play a role in connecting centers for this purpose.

Monitoring and evaluation

We intend to employ the European Pediatric Surgical Audit (EPSA) as a continuous feedback mechanism to monitor and evaluate (recommendation-specific) implementation success. The EPSA is an international prospective clinical audit registry. (The need for) supplementary measures for validation purposes will also be explored.

It is also of note that genetic testing is a fast-moving landscape. Whole genome sequencing may be a possibility (in some countries) in the near future. This should be considered during the implementation process, as well as in the updated version of the guideline (in approximately 5 years).

Research priorities

To increase certainty of evidence, prospective data on the diagnostic yield of WES specifically for omphalocele patients is desirable.

REFERENCES SUMMARY

Que et al. 2023 (reference 10)

Mellis et al. 2022 (reference 11)

Shi et al. 2021 (reference 12)

Bauskis et al. 2022 (reference 13)

1. Brioude, F., Kalish, J., Mussa, A. et al. Clinical and molecular diagnosis, screening and management of Beckwith–Wiedemann syndrome: an international consensus statement. Nat Rev Endocrinol 14, 229–249 (2018). https://doi.org/10.1038/nrendo.2017.166

QUESTION 1.2

Should cesarean delivery vs. vaginal birth be used for delivery of babies with small omphalocele?	
**POPULATION:**	Delivery of babies with small omphalocele
**INTERVENTION:**	Cesarean delivery
**COMPARISON:**	Vaginal birth
**MAIN OUTCOMES:**	Neonatal mortality, Length of hospital stay and Time to full feeding

ASSESSMENT

Problem
Is the problem a priority?

JUDGEMENT	RESEARCH EVIDENCE	ADDITIONAL CONSIDERATIONS
O No<br>O Probably no<br>O Probably yes<br>O Yes<br>O Varies<br>O Don't know		

Desirable Effects
How substantial are the desirable anticipated effects?

JUDGEMENT	RESEARCH EVIDENCE	ADDITIONAL CONSIDERATIONS
O Trivial<br>O Small<br>O Moderate	How et al. (2000) reported on a group of 32 prenatally diagnosed omphalocele patients. All fetuses had small omphaloceles with no liver herniation. Twenty-eight had elective cesarean delivery, four were delivered vaginally. There	EPSA summary<br><br>Mortality: Most panel members indicated small harm but with notice that if we don't know the cause of death we do not know if this is related to the mode of birth. Panel members indicated that they

○ Large<br>● Varies<br>○ Don't know	was no significant difference between the groups, the neonatal mortality rate was 7% in the cesarean delivery group and 25% in the vaginal delivery group (p=0.2). The median length of stay was 39 days (IQR 25-56) for the cesarean delivery and 24 days (IQR 8-71) for the vaginal delivery (p=0.004, with Mann-Whitney test). The median time to enteral feeding was 12 days (IQR 6, 15) for the cesarean group and 16 days (IQR 3, 29) for the vaginal delivery group (p=0.03 with Mann-Whitney test).	believe associated anomalies such as cardiac defect have more influence on mortality rate than mode of delivery.<br><br>Duration of ventilation: Majority of panel members indicated small benefit.<br><br>Time to first feed: Majority of panel members indicated no effect.<br><br>Time to full feeds: Three panel members indicated small benefit, others interpretated data as no effect.<br><br>Panel discussion<br><br>The panel agreed that based on the analyzed literature and EPSA data the benefits of vaginal delivery are probably small. The literature analyzed indicated some benefits of vaginal delivery on the outcome length of stay. Panel members are, however, unsure to what extent the mode of delivery is related to the length of stay in this small sample. The observations based on EPSA data suggest small benefits of vaginal delivery considering the outcome 'duration of ventilation' and 'time to full feeds'. Panel members discussed that duration of ventilation may also be related to gestational age, associated cardiac abnormalities and lung hypoplasia, but then again observed no important differences between groups on these factors. This supports their confidence in the observed benefits of vaginal delivery, but many panel members still had doubts about the correlation between the mode of delivery and the evaluated outcomes. Besides effects for the baby, the panel also considered the benefits and harms of the different modes of delivery for the mother. The panel agrees that there are considerable benefits of vaginal delivery for the mother. These may be judged as moderate to large.

Undesirable Effects

How substantial are the undesirable anticipated effects?

JUDGEMENT	RESEARCH EVIDENCE	ADDITIONAL CONSIDERATIONS
● Trivial<br>O Small<br>O Moderate<br>O Large<br>O Varies<br>O Don't know	No undesirable effects could be found in the literature analysis.	EPSA summary<br>About half of the panel observed a small harm of vaginal delivery as the mortality seemed slightly higher in the vaginal delivery group. As the cause of death was unknown, the other half of the panel observed no effect or indicated 'don't know'. The panel chose not to consider this in the balance of effects as the relation between mode of delivery and mortality could not be clarified from the presented EPSA data.<br><br>Panel discussion<br>The panel agrees that no considerable harms of vaginal delivery were found in the analysis and EPSA data. The risk of rupture of the omphalocele during a vaginal delivery was briefly discussed but the panel members agreed that this is not a realistic risk for patients with a non-giant omphalocele. Should a rupture of the sac occur in small omphaloceles, the sac can be sutured closed again.

Certainty of evidence

What is the overall certainty of the evidence of effects?

JUDGEMENT	RESEARCH EVIDENCE	ADDITIONAL CONSIDERATIONS
● Very low<br>O Low<br>O Moderate<br>O High<br>O No included studies	Analyses are not corrected for possible confounders; data is collected over a spread period of time (10 years) in which interventions and management may have changed. There was also a very small sample (n=4) in the vaginal delivery group -this likely induces imprecision of the effect estimate.	

Values

Is there important uncertainty about or variability in how much people value the main outcomes?

JUDGEMENT	RESEARCH EVIDENCE	ADDITIONAL CONSIDERATIONS
○ Important uncertainty or variability<br>● Possibly important uncertainty or variability<br>○ Probably no important uncertainty or variability<br>○ No important uncertainty or variability		Besides mortality, the evaluated outcomes are proxies for the condition of the baby. The panel thinks it is unlikely parents and clinicians value this differently. In terms of intervention and the outcomes for mother, avoiding a cesarean delivery may be of higher value to some expecting mothers than others.

Balance of effects

Does the balance between desirable and undesirable effects favor the intervention or the comparison?

JUDGEMENT	RESEARCH EVIDENCE	ADDITIONAL CONSIDERATIONS
○ Favors the comparison<br>○ Probably favors the comparison<br>○ Does not favor either the intervention or the comparison<br>● Probably favors the intervention<br>○ Favors the intervention	Associated anomalies are not a reason to advise against vaginal delivery.	The panel concluded that the balance of effects probably favors the intervention as there are minimal beneficial effects for the baby, moderate to large beneficial effects for the mother and no considerable harm observed to vaginal delivery. No evidence was found that in patients with a non-giant omphalocele, associated anomalies such as cardiac defects should be considered as a contra-indication for vaginal delivery. Based on additional considerations and expert opinion, the panel members see no reason to deviate from the standard obstetrical protocol.

O Varies<br>O Don't know		

Resources required

JUDGEMENT	RESEARCH EVIDENCE	ADDITIONAL CONSIDERATIONS
O Large costs<br>O Moderate costs<br>● Negligible costs and savings<br>O Moderate savings<br>O Large savings<br>O Varies<br>O Don't know		The panel discussed that there are probably negligible costs or savings associated with the mode of delivery. As for the intervention itself, no further training or need for specialist clinicians is needed to deliver babies with small omphaloceles vaginally, meaning a trivial impact on hospital resources.<br><br>The costs of vaginal delivery per patient are lower than cesarean delivery, however overall, the difference would be marginal since the number of patients with omphalocele is so few. Some panel members argue that we should also see the difference in costs considering the costs of neonatal care that could increase or decrease with outcome. However, the panel agrees that we have too little data at the moment to see if one or both interventions would lead to reduced costs.

Certainty of evidence of required resources

What is the certainty of the evidence of resource requirements (costs)?

JUDGEMENT	RESEARCH EVIDENCE	ADDITIONAL CONSIDERATIONS
O Very low<br>O Low<br>O Moderate<br>O High<br>● No included studies		

Cost effectiveness

Does the cost-effectiveness of the intervention favor the intervention or the comparison?

JUDGEMENT	RESEARCH EVIDENCE	ADDITIONAL CONSIDERATIONS
O Favors the comparison<br>O Probably favors the comparison<br>O Does not favor either the intervention or the comparison<br>O Probably favors the intervention<br>O Favors the intervention<br>O Varies<br>● No included		

studies		

Equity

What would be the impact on health equity?

JUDGEMENT	RESEARCH EVIDENCE	ADDITIONAL CONSIDERATIONS
O Reduced<br>O Probably reduced<br>● Probably no impact<br>O Probably increased<br>O Increased<br>O Varies<br>O Don't know		Panel members agreed that both options are widely available, which makes the impact of a favorable recommendation for one or the other on care equity marginal.

Acceptability

Is the intervention acceptable to key stakeholders?

JUDGEMENT	RESEARCH EVIDENCE	ADDITIONAL CONSIDERATIONS
O No<br>O Probably no<br>● Probably yes<br>O Yes<br>O Varies		The panel concluded that a recommendation in favor of vaginal delivery would probably be acceptable. There could however be some barriers pertaining to the organization of care. In some centers, all neonates with omphalocele, also the non-giant omphaloceles, go to the NICU and some teams may be short staffed- there may be a

O Don't know		preference for a planned delivery so that the appropriate organizational provisions can be made.<br><br>Patients may have a strong preference for the delivery method due to a number of personal reasons.

Feasibility
Is the intervention feasible to implement?

JUDGEMENT	RESEARCH EVIDENCE	ADDITIONAL CONSIDERATIONS
O No<br>O Probably no<br>O Probably yes<br>● Yes<br>O Varies<br>O Don't know		The panel agreed that despite the barriers, the recommendation would be feasible to implement when teams are well informed about the rationale behind this recommendation. Vaginal and cesarean deliveries are both widely available within all hospitals treating omphalocele patients.

SUMMARY OF JUDGEMENTS

	JUDGEMENT						
**PROBLEM**	No	Probably no	Probably yes	Yes		Varies	Don't know
**DESIRABLE EFFECTS**	Trivial	Small	Moderate	Large		**Varies**	Don't know
**UNDESIRABLE EFFECTS**	**Trivial**	Small	Moderate	Large		Varies	Don't know
**CERTAINTY OF EVIDENCE**	**Very low**	Low	Moderate	High			No included studies
**VALUES**	Important uncertainty or variability	**Possibly important uncertainty or variability**	Probably no important uncertainty or variability	No important uncertainty or variability			

	JUDGEMENT						
BALANCE OF EFFECTS	Favors the comparison	Probably favors the comparison	Does not favor either the intervention or the comparison	**Probably favors the intervention**	Favors the intervention	Varies	Don't know
RESOURCES REQUIRED	Large costs	Moderate costs	**Negligible costs and savings**	Moderate savings	Large savings	Varies	Don't know
CERTAINTY OF EVIDENCE OF REQUIRED RESOURCES	Very low	Low	Moderate	High			**No included studies**
COST EFFECTIVENESS	Favors the comparison	Probably favors the comparison	Does not favor either the intervention or the comparison	Probably favors the intervention	Favors the intervention	Varies	**No included studies**
EQUITY	Reduced	Probably reduced	**Probably no impact**	Probably increased	Increased	Varies	Don't know
ACCEPTABILITY	No	Probably no	**Probably yes**	Yes		Varies	Don't know
FEASIBILITY	No	Probably no	Probably yes	**Yes**		Varies	Don't know

TYPE OF RECOMMENDATION

Strong recommendation against the intervention	Conditional recommendation against the intervention	Conditional recommendation for either the intervention or the comparison	Conditional recommendation for the intervention	**Strong recommendation for the intervention**
○	○	○	○	●

CONCLUSIONS

Recommendation

The panel recommends vaginal delivery for non-giant omphalocele babies unless obstetrical considerations would warrant otherwise.

Justification

Balance of effects

The panel concludes that the balance of effects probably favors the intervention as there are minimal beneficial effects for the baby, moderate to large beneficial effects for the mother and no considerable harm observed for vaginal delivery. No evidence was found that in patients with a non-giant omphalocele, associated anomalies such as cardiac defects should be considered as a contra-indication for vaginal delivery. Based on additional considerations and expert opinion, the panel members see no reason to deviate from the standard obstetrical protocol.

Subgroup considerations

This recommendation is specific to patients with a non-giant omphalocele.

Implementation considerations

To gain insight into current practices pertaining to the management of omphalocele in Europe, a baseline survey has been conducted amongst centers involved in the European Reference Network for rare Inherited Congenital Anomalies (ERNICA) and/or connected to the European Pediatric Surgical Audit (EPSA). Center representatives are asked what their center's preferred mode of delivery is for babies with non-giant (isolated) omphalocele, and they have the opportunity to provide additional explanation.

A qualitative study will also take place to further explore the factors foreseen to hinder/facilitate successful implementation of this recommendation in clinical practice. This will be done with a view to collaboratively selecting implementation strategies.

Monitoring and evaluation

We intend to employ the European Pediatric Surgical Audit (EPSA) as a continuous feedback mechanism to monitor and evaluate (recommendation-specific) implementation success. The EPSA is an international prospective clinical audit registry. (The need for) supplementary measures for validation purposes will also be explored.

Research priorities

The mode of delivery for patients with a giant omphalocele was not selected as a priority topic for this guideline. However, during discussions, the group discovered significant practice variability between group members as to the mode of delivery for giant omphalocele patients. This will be subject to evaluation during the periodic update of this guideline.

REFERENCES SUMMARY

How et al. (2000) (reference 14)

QUESTION 1.3A

Should liver herniation vs. no liver herniation be used for predicting neonatal outcomes?	
**POPULATION:**	Predicting neonatal outcomes
**INTERVENTION:**	Liver herniation
**COMPARISON:**	No liver herniation
**MAIN OUTCOMES:**	Neonatal mortality, Adverse outcome and Ability to perform primary repair

ASSESSMENT

Problem<br>Is the problem a priority?		
JUDGEMENT	RESEARCH EVIDENCE	ADDITIONAL CONSIDERATIONS
O No<br>O Probably no<br>O Probably yes<br>O Yes<br>O Varies<br>O Don't know		
**Desirable Effects**<br>How substantial are the desirable anticipated effects?		
JUDGEMENT	RESEARCH EVIDENCE	ADDITIONAL CONSIDERATIONS
O Trivial<br>O Small<br>O Moderate	For the factor prenatally detected liver herniation, five studies were included for analysis (Connor, 2018; Chock, 2019; Montero, 2011; Nicholas, 2009; Hidaka, 2009). The literature was analyzed for outcomes: neonatal mortality,	The panel concluded that measuring liver herniation prenatally probably has large benefits. These benefits are related to counselling parents (including counselling about the choice between continuation or termination of the pregnancy if this is parents' wish to discuss) and

● Large<br>O Varies<br>O Don't know	adverse neonatal outcome and ability to perform primary repair. The panel concluded that there are indications prenatally detected extracorporeal liver is a predictor for a higher mortality rate and more adverse neonatal outcomes compared to intracorporeal liver and that there are indications prenatally detected extracorporeal liver is an independent predictor for the inability to perform primary repair.	to optimally prepare for birth. Panel members agree that prediction of the ability to perform primary closure could make a huge difference in preparing parents for the neonatal period. It is important to note that this measurement should be interpreted with caution during the first trimester as the image may change when the baby grows around the defect. Maternal and fetal medicine specialists indicate the second trimester as the optimal period for the evaluation of liver herniation.

Undesirable Effects

How substantial are the undesirable anticipated effects?

JUDGEMENT	RESEARCH EVIDENCE	ADDITIONAL CONSIDERATIONS
● Trivial<br>O Small<br>O Moderate<br>O Large<br>O Varies<br>O Don't know		The panel concluded that the undesirable effects of measuring liver herniation and including this in counselling are probably trivial. Although there is not a specific definition of liver herniation, maternal and fetal medicine specialists agree that it is not a difficult measurement and it has low inter-operator variability as you judge the liver to be either in or out, and the amount of liver herniated is not measured or classified. The one possible undesirable effect could be that the certainty of evidence is low, and counselling based on herniated liver could therefore lead to a more negative scenario.

Certainty of evidence<br>What is the overall certainty of the evidence of effects?		
JUDGEMENT	RESEARCH EVIDENCE	ADDITIONAL CONSIDERATIONS
○ Very low<br>● Low<br>○ Moderate<br>○ High<br>○ No included studies	In all studies, only percentages of mortality incidence could be compared, while no correction for possible confounders was applied. Both small series and zero incidents of death in the control group of Hidaka (2009) likely introduced imprecision. For the outcome 'adverse neonatal outcomes', results between the two included studies were conflicting. In one study, extracorporeal liver was significantly associated with adverse outcome- this remained in multivariate analysis (Nicholas). In the other study (Chock), extracorporeal liver was not associated with adverse outcome (death or prolonged length of stay). Both studies had very small series which likely introduce imprecision.<br><br>Thereby, 'adverse neonatal outcomes' is a combined outcome with severe and less severe events for which the definition differs between studies.<br><br>For the outcome ‘ability to perform primary repair’ there is possible selection bias due to referrals of more complicated cases to the center where this study was conducted. Also, 16.4% of eligible patients were excluded for incomplete patient data. In the study by Peters et al., nine patients were lost to follow-up because they were born in another center; it is unknown if the characteristics of these patients would have influenced the prognostic factor.	

Values<br>Is there important uncertainty about or variability in how much people value the main outcomes?		
JUDGEMENT	RESEARCH EVIDENCE	ADDITIONAL CONSIDERATIONS
○ Important uncertainty or variability		The panel concluded that there are likely no important differences in how stakeholders value the outcomes. Parents would probably want to know how likely it is that their child will fall into a certain category,

O Possibly important uncertainty or variability<br>● Probably no important uncertainty or variability<br>O No important uncertainty or variability		for example how long they can expect their child to be in the hospital. The chance of primary closure, for example, relates to the length of stay. Clinicians in turn will benefit from this information, informing their preparation for the perinatal care of the patient.

Balance of effects

Does the balance between desirable and undesirable effects favor the intervention or the comparison?

JUDGEMENT	RESEARCH EVIDENCE	ADDITIONAL CONSIDERATIONS
O Favors the comparison<br>O Probably favors the comparison<br>O Does not favor either the intervention or the comparison<br>● Probably favors the intervention<br>O Favors the intervention<br>O Varies<br>O Don't know		The panel concluded that the balance of effects probably favors including liver herniation as a prognostic factor. As there are many confounding variables and low evidence quality for all included studies, panel members emphasize this factor as just one piece of the puzzle. The panel agrees that if used, this prognostic factor should be considered in combination with other factors to get the most reliable prediction.

Resources required

JUDGEMENT	RESEARCH EVIDENCE	ADDITIONAL CONSIDERATIONS
O Large costs<br>O Moderate costs<br>● Negligible costs and savings<br>O Moderate savings<br>O Large savings<br>O Varies<br>O Don't know		The panel concluded there are probably negligible costs and savings as (repeated) prenatal ultrasound is done in (almost) all patients and the detection rate of liver herniation is very high. No additional resources are required to look at liver herniation.

Certainty of evidence of required resources

What is the certainty of the evidence of resource requirements (costs)?

JUDGEMENT	RESEARCH EVIDENCE	ADDITIONAL CONSIDERATIONS
O Very low<br>O Low<br>O Moderate<br>O High<br>● No included studies		

Cost effectiveness<br>Does the cost-effectiveness of the intervention favor the intervention or the comparison?		
JUDGEMENT	RESEARCH EVIDENCE	ADDITIONAL CONSIDERATIONS
O Favors the comparison<br>O Probably favors the comparison<br>O Does not favor either the intervention or the comparison<br>O Probably favors the intervention<br>O Favors the intervention<br>O Varies<br>● No included studies		

Equity<br>What would be the impact on health equity?		
JUDGEMENT	RESEARCH EVIDENCE	ADDITIONAL CONSIDERATIONS
O Reduced<br>O Probably reduced<br>O Probably no impact<br>● Probably increased<br>O Increased<br>O Varies<br>O Don't know	Panel voice:<br>Probably no impact = 43%<br>Probably increased = 38%	The panel concluded that measuring this prognostic factor probably results in increased care equity as it could allow for early referral of patients with extracorporeal liver to expert care centers as they are potentially more complex patients.

Acceptability
Is the intervention acceptable to key stakeholders?

JUDGEMENT	RESEARCH EVIDENCE	ADDITIONAL CONSIDERATIONS
O No<br>O Probably no<br>O Probably yes<br>● Yes<br>O Varies<br>O Don't know		The panel members expect that the use of liver herniation as a prognostic factor in the prenatal counselling program is acceptable to parents and clinicians.<br><br>However, it is of note that clinicians may question how they should measure liver herniation and if it is important to evaluate the amount of liver out. Currently, there is no distinction between a little and a lot of liver herniation and this variable is measured as a yes or no factor.

Feasibility
Is the intervention feasible to implement?

JUDGEMENT	RESEARCH EVIDENCE	ADDITIONAL CONSIDERATIONS
O No<br>O Probably no<br>O Probably yes<br>● Yes<br>O Varies<br>O Don't know		The panel members expect that the use of this prognostic factor in the prenatal counselling program is feasible for all key stakeholders as no additional materials, appointments or money is required compared to not measuring liver herniation on the prenatal ultrasound.

SUMMARY OF JUDGEMENTS

	JUDGEMENT						
**PROBLEM**	No	Probably no	Probably yes	Yes		Varies	Don't know
**DESIRABLE EFFECTS**	Trivial	Small	Moderate	**Large**		Varies	Don't know
**UNDESIRABLE EFFECTS**	**Trivial**	Small	Moderate	Large		Varies	Don't know

	JUDGEMENT						
CERTAINTY OF EVIDENCE	Very low	**Low**	Moderate	High			No included studies
VALUES	Important uncertainty or variability	Possibly important uncertainty or variability	**Probably no important uncertainty or variability**	No important uncertainty or variability			
BALANCE OF EFFECTS	Favors the comparison	Probably favors the comparison	Does not favor either the intervention or the comparison	**Probably favors the intervention**	Favors the intervention	Varies	Don't know
RESOURCES REQUIRED	Large costs	Moderate costs	**Negligible costs and savings**	Moderate savings	Large savings	Varies	Don't know
CERTAINTY OF EVIDENCE OF REQUIRED RESOURCES	Very low	Low	Moderate	High			**No included studies**
COST EFFECTIVENESS	Favors the comparison	Probably favors the comparison	Does not favor either the intervention or the comparison	Probably favors the intervention	Favors the intervention	Varies	**No included studies**
EQUITY	Reduced	Probably reduced	Probably no impact	**Probably increased**	Increased	Varies	Don't know
ACCEPTABILITY	No	Probably no	Probably yes	**Yes**		Varies	Don't know
FEASIBILITY	No	Probably no	Probably yes	**Yes**		Varies	Don't know

TYPE OF RECOMMENDATION

Strong recommendation against the intervention	Conditional recommendation against the intervention	Conditional recommendation for either the intervention or the comparison	**Conditional recommendation for the intervention**	Strong recommendation for the intervention

○	○	○	●	○

CONCLUSIONS

Recommendation

The panel suggests using extracorporeal (part/whole) liver herniation (assessed in the 2nd trimester) as a prognostic factor in combination with other prenatal measurements (e.g. the OC/AC ratio). This is preferably done in a research or registry setting as prospective data collection is warranted to further confirm the reliability of this measurement for predicting neonatal outcome.

Justification

Balance of effects

The panel concludes that the balance of effects probably favors including liver herniation as a prognostic factor. As there are many confounding variables and low evidence quality for all included studies, panel members emphasize this factor as just one piece of the puzzle. The panel agrees that if used, this prognostic factor should be considered in combination with other factors to get a more reliable prediction.

Subgroup considerations

The panel did not formulate any specific subgroup considerations.

Implementation considerations

To gain insight into current practices pertaining to the management of omphalocele in Europe, a baseline survey has been conducted amongst centers involved in the European Reference Network for rare Inherited Congenital Anomalies (ERNICA) and/or connected to the European Pediatric Surgical Audit (EPSA). Center representatives are asked whether their center assesses liver herniation prenatally, as well as whether the presence of liver herniation is used as a prognostic factor (amongst other factors) for postnatal outcome, either for the purpose of counselling or care planning. Opportunities are provided for further explanation.

ERNICA can play a role in promoting and facilitating opportunities for prospective data collection, through multicenter research initiatives and via the EPSA. ERNICA may also help facilitate educational activities on prenatal assessment.

Monitoring and evaluation

We intend to employ the European Pediatric Surgical Audit (EPSA) as a continuous feedback mechanism to monitor and evaluate (recommendation-specific) implementation success. The EPSA is an international prospective clinical audit registry. (The need for) supplementary measures for validation purposes will also be explored.

Since prenatal data is often stored in the mother's file (which is often not accessible to pediatric surgeons), efforts to promote coordinated patient registration on a local level are required. The ERNICA/EPSA coordination teams will strive to facilitate initiatives to support local teams with such efforts.

Research priorities

Prospective data collection showing the relationship between prenatal liver herniation and neonatal outcome is desirable. Strict instructions for timing of measurement, amount of repeat measurements and definitions for neonatal outcomes are imperative to establish higher certainty of evidence. Including this in the revision of the EPSA data set is a possibility, although there are barriers to the accurate registration of prenatal data as this is often included in the mother's medical file only.

REFERENCES SUMMARY

Chock, 2019 (reference 15)

Connor, 2018 (reference 16)

Hidaka, 2009 (reference 17)

Montero, 2011 (reference 18)

Nicholas, 2009 (reference 19)

Peters et al. 2019 (reference 24)

QUESTION 1.3B

Should omphalocele ratios vs. be used for predicting neonatal outcomes?	
**POPULATION:**	Predicting neonatal outcomes
**INTERVENTION:**	Measurement of omphalocele ratios
**COMPARISON:**	
**MAIN OUTCOMES:**	Mortality, Adverse neonatal outcome, Ability to perform primary repair and Respiratory insufficiency

ASSESSMENT

Problem<br>Is the problem a priority?		
JUDGEMENT	RESEARCH EVIDENCE	ADDITIONAL CONSIDERATIONS
O No<br>O Probably no<br>O Probably yes<br>O Yes<br>O Varies<br>O Don't know		
**Desirable Effects**<br>How substantial are the desirable anticipated effects?		
JUDGEMENT	RESEARCH EVIDENCE	ADDITIONAL CONSIDERATIONS
O Trivial<br>O Small<br>O Moderate<br>● Large	It is likely there is a significant negative association between the omphalocele circumference/abdominal circumference (**OC/AC)** ratio (measured between 17 and 38 weeks gestation) and survival. In a study by Peters et al. OC/AC ratio was measured at three points	The panel concluded that measuring omphalocele ratios possibly has large benefits. The ability to predict these outcomes could be highly valuable in counselling and peri- and post-natal planning. Particularly the OC/AC ratio seems a reliable and reproductible

○ Varies<br>○ Don't know	during gestation: US1: 11-16 weeks US2: 17-26 weeks US3: 30-38 weeks. The OC/AC ratio could be calculated for 22 fetuses at US1, for 50 at US2, and for 58 at US3. Separate ROC analyses for each of the three- measurement time periods revealed a statistically significant negative association between the OC/AC ratio and survival at US2 and US3. At US2 an AUC of 0.81 (with a 95% CI of 0.61–1.00; p = .01), and at US3 an AUC of 0.89 (with a 95% CI of 0.79–0.98; p = .001).<br><br>It is likely that an **OC/AC** ratio is significantly associated with the probability of requiring delayed or staged closure. The optimal cut-off probably varies with gestational age. Kleinrouweler et al. (2011) reported over 13 cases that all cases with a primary reconstruction had OC/AC ratios below 0.57, all cases with staged closure had higher OC/AC ratios above 0.75. Thus, any cutoff between 0.57 and 0.75 has 100% sensitivity and specificity.<br><br>Peters et al. (2019) reported that the OC/AC ratio was significantly positively associated with the probability of requiring a delayed closure at all three measured time periods. Based on ROC curve analysis, the type of closure was predicted correctly by the OC/AC ratio with optimal cut-off values of 0.69 at US1 (sensitivity 0.93 and specificity 0.90; AUC 0.96, 0.88–1.00; p < .001), 0.66 at US2 (sensitivity 0.88 and specificity 0.93; AUC 0.98, 0.95–1.00; p < .001), and 0.63 at US3 (sensitivity 0.95 and specificity 0.94; AUC 0.98, 0.95–1.00; p < .001)<br><br>It is likely that there is a trend towards more respiratory insufficiency in patients with higher **OC/AC** ratios. Kleinrouweler et al. (2011) reported over 15 cases, there was a trend towards more respiratory insufficiency for cases with higher OC/AC ratio: for a 0.10-point increase in the OC/AC ratio, the OR for respiratory insufficiency was 1.59 (95% CI = 0.95–2.67).<br><br>The optimal cut-off for predicting respiratory insufficiency was found to be 0.66 with a sensitivity of 67%, a specificity of 78% and an AUC of 0.76.	prognostic factor that may improve the quality of prenatal counselling. The study by Tassin et al. (2013) indicates that measurement of the viscero-abdominal disproportion may have prognostic value as early as the first trimester.

	There are indications that **OD/AC** ratios >0.26 are associated with a higher likelihood of death. Fawley et al. (2016) looked at OD/AC ratios in 30 patients. The optimal cut-off was determined 0.26. There was no difference in mortality between patients with a cutoff below (1/20) or above (1/10) the optimal cut-off (p=1). However, a study by Kiyohara et al. (2014) looked at OD/AC ratios in 47 patients with the same cut-off of 0.26. In this study, a OD/AC ratio above 0.26 in the first scan was associated with a higher likelihood of neonatal death (Likelihood ratio 4, 95% CI 1.9-7.5).<br><br>It is likely **OD/AC** ratio is not associated with the probability of one or more adverse outcomes (death before discharge or prolonged LoS) . Chock et al. assessed the AWD/AC ratio of patients in this cohort was compared against a threshold of 0.24 (defined by Montero et al. 2011). AWD/AC >0.24 was associated with adverse outcome (p=0.02). However, after correction for confounders associated with death or length of stay, the association was not significant anymore (OR 2.8 95%CI 0.01–520, p=0.70).<br><br>It is likely that an **OD/AC** ratio above 0.24-0.26 is predictive for inability of primary repair of the omphalocele. In the study by Montero et al. (2011) (n=24) the OD/AC ratio was predictive for the inability to primary closure with a cut-off of >/=0.24 (OR 7, 95%CI 1.1-41.3). The Sensitivity was 83.3, Specificity was 58.3, PPV 66.7 and NPV 77.8 with an AUC of 0.71. Nitsche et. al. found in their series of 17 patients that OD/AC ratio measured at the **maximum** omphalocele diameter with a optimal cut-off of >/=0.24 has 100% specificity to predict inability of primary repair. Kiyohara et al. (n=35) found that OD/AC ratio with a cut-off of >/=0.26, is predictive for the need of a two-step surgery or the use of mesh (p<0.001). Fawley et al. used an optimal cut-off of 0.26. Sixty percent (12/20) in the low-ratio group achieved primary closure versus zero (0/10) in the high-ratio group (p = 0.001). All four studies conclude that OD/AC ratios above 0.24-0.26 are predictive for inability of primary repair.<br><br>There are indications that an **OD/AC** ratio above 0.26 is predictive for intubation in the first 24 hours of life and longer need for	

	mechanical ventilation. Fawley et al. (n=30) report that patients with a OD/AC ratio above 0.26 had significantly longer ventilation times (15.8 days (low-ratio) versus 79 days (high-ratio) (p = 0.05)). In the study of Kiyohara et al. more patients with a ratio above 0.26 needed intubation in the first 24h of life (4/21 (19%) vs. 8/14 (57.1%) , p=0.03). A ratio above 0.26 seemed also predictive for prolonged ventilation (>21 days) but due to low incidence within the sample, this could not be proven significant.<br><br>Last, there are indications that measurement of the viscero-abdominal disproportion can be valuable as early as the first trimester. Tassin et al. (2013) included 54 fetusses with omphalocele and measured the omphalocele diameter/transabdominal diameter before 14 weeks gestation. They found a significant increase in morbidity (hospitalization in the intensive care unit for more than 42 days, need for respiratory assistance (mechanical ventilation, intubation) for more than 21 days and/or need for parenteral feeding for more than 21 days) in patients with a MOD/TAD ratio >0.8.	

Undesirable Effects

How substantial are the undesirable anticipated effects?

JUDGEMENT	RESEARCH EVIDENCE	ADDITIONAL CONSIDERATIONS
○ Trivial<br>● Small<br>○ Moderate<br>○ Large<br>○ Varies<br>○ Don't know		The panel agreed that the undesirable effects of measurement and prognosis based on omphalocele ratios is probably small. However, it should be recognized that unlike liver herniation, these measurements require specific skills and training. If measurements are done incorrectly, we are even more unsure of their predictive value which could, in the worst case, result in pregnancy termination based on incorrect values.

Certainty of evidence

What is the overall certainty of the evidence of effects?

JUDGEMENT	RESEARCH EVIDENCE	ADDITIONAL CONSIDERATIONS
○ Very low<br>● Low<br>○ Moderate<br>○ High<br>○ No included studies	Certainty of evidence for **OC/AC** ratios is moderate (outcomes: mortality, ability to perform primary repair, adverse outcomes and respiratory insufficiency). The certainty was rated down because the analysis was not corrected for important confounders such as congenital lung disease or other concomitant factors. Also, nine patients were lost to follow-up because they were born in another center, it is unknown if the characteristics of these patients would have influenced the prognostic factor.<br><br>The certainty of evidence for other outcomes (Mortality, Adverse outcomes, Ability to perform primary repair and Respiratory problems linked to **OD/AC** ratio) was low to moderate. Certainty of evidence was rated down for possible attrition bias: it's unclear if patients with images available/included patients had different characteristics than patients eligible without available images. As only univariate analyses were performed, there are concerns for bias due to confounding. Concerning the predictive value for mortality, studies had conflicting results. This may be inconsistency but may also be due to a lack of statistical power in the study of Fawley. Concerning the predictive value for need of intubation and mechanical ventilation there are concerns for imprecision because of the small sample size and low incidence of sought effect and possibly lack of statistical power in the analysis of Kiyohara. The evidence for adverse outcomes had a very wide confidence interval (OR 2.8 95%CI 0.01–520) which raises concerns for imprecision.	The panel emphasizes that larger studies are still needed to validate this prognostic factor fully and define more specific cut offs, terms of measurement for each outcome and subgroups with different grades of severity.

Values

Is there important uncertainty about or variability in how much people value the main outcomes?

JUDGEMENT	RESEARCH EVIDENCE	ADDITIONAL CONSIDERATIONS
○ Important uncertainty or		The panel concluded that there are likely no important differences in how stakeholders value the outcomes. Parents would probably

variability<br>○ Possibly important uncertainty or variability<br>● Probably no important uncertainty or variability<br>○ No important uncertainty or variability		want to know how likely it is that their child will fall into a certain category and clinicians will benefit from this information in their preparation for the perinatal care of the patient.
**Balance of effects**<br>Does the balance between desirable and undesirable effects favor the intervention or the comparison?		
JUDGEMENT	RESEARCH EVIDENCE	ADDITIONAL CONSIDERATIONS
○ Favors the comparison<br>○ Probably favors the comparison<br>○ Does not favor either the intervention or the comparison<br>○ Probably favors the intervention<br>● Favors the intervention<br>○ Varies<br>○ Don't know		The panel members agreed that the balance of effects is likely in favor of measuring one or more omphalocele ratio. As omphalocele is a birth defect with possibly severe consequences, collecting as much information as possible before counselling is likely beneficial to all stakeholders. The difference between the omphalocele diameter/abdominal circumference (OD/AC) and OC/AC is discussed and as some panel members indicated that OD/AC probably has a smaller risk for measuring errors, the available evidence for OC/AC is stronger. In a previous study by Peters et al. (2014) both ratios were compared and OC/AC seemed more reliable as a prognostic factor.<br><br>While there is a definition for OC/AC ratio proposed by Peters et al. (2019), some panel members argue that the definition is still unclear. There are also other uncertainties concerning the level and at what gestational age(s) these measurements should be done. From second trimester up to 32 weeks is suggested by maternal and fetal medicine specialists on the panel as an appropriate timeframe for repeated measures.

Resources required		
JUDGEMENT	RESEARCH EVIDENCE	ADDITIONAL CONSIDERATIONS
O Large costs<br>O Moderate costs<br>● Negligible costs and savings<br>O Moderate savings<br>O Large savings<br>O Varies<br>O Don't know		The panel concluded that there are probably negligible costs and savings as (repeated) prenatal ultrasound is done in (almost) all patients. However, some resources are needed in the form of training team members to perform the measurement.

Certainty of evidence of required resources<br>What is the certainty of the evidence of resource requirements (costs)?		
JUDGEMENT	RESEARCH EVIDENCE	ADDITIONAL CONSIDERATIONS
O Very low<br>O Low<br>O Moderate<br>O High<br>● No included studies		

Cost effectiveness

Does the cost-effectiveness of the intervention favor the intervention or the comparison?

JUDGEMENT	RESEARCH EVIDENCE	ADDITIONAL CONSIDERATIONS
O Favors the comparison<br>O Probably favors the comparison<br>O Does not favor either the intervention or the comparison<br>O Probably favors the intervention<br>O Favors the intervention<br>O Varies<br>● No included studies		

Equity

What would be the impact on health equity?

JUDGEMENT	RESEARCH EVIDENCE	ADDITIONAL CONSIDERATIONS
O Reduced<br>O Probably reduced<br>O Probably no impact<br>● Probably increased<br>O Increased<br>O Varies<br>O Don't know		The panel concluded that measuring this prognostic factor probably results in increased care equity as it could allow for early referral of patients with worse prognosis to expert care centers and enable parents to be informed more precisely.

Acceptability
Is the intervention acceptable to key stakeholders?

JUDGEMENT	RESEARCH EVIDENCE	ADDITIONAL CONSIDERATIONS
O No<br>O Probably no<br>O Probably yes<br>● Yes<br>O Varies<br>O Don't know		The panel agreed that a recommendation in favor would be acceptable. In most centers, ratios are already used in counselling and parents are informed about the current state of knowledge and the reliability of predictions at this point. However, it is of note that in some centers, clinicians may not currently use the omphalocele ratios and may therefore question how they should measure and evaluate omphalocele ratios.

Feasibility
Is the intervention feasible to implement?

JUDGEMENT	RESEARCH EVIDENCE	ADDITIONAL CONSIDERATIONS
O No<br>O Probably no<br>O Probably yes<br>● Yes<br>O Varies<br>O Don't know		The panel agreed that a recommendation in favor is feasible but maternal and fetal medicine specialists working with these measures do need focused training on how to perform these measurements. As in some member hospitals of ERNICA there are specialists with a lot of expertise on this, training could be organised within the network.

SUMMARY OF JUDGEMENTS

	JUDGEMENT						
**PROBLEM**	No	Probably no	Probably yes	Yes		Varies	Don't know
**DESIRABLE EFFECTS**	Trivial	Small	Moderate	**Large**		Varies	Don't know
**UNDESIRABLE EFFECTS**	Trivial	**Small**	Moderate	Large		Varies	Don't know

	JUDGEMENT						
CERTAINTY OF EVIDENCE	Very low	**Low**	Moderate	High			No included studies
VALUES	Important uncertainty or variability	Possibly important uncertainty or variability	**Probably no important uncertainty or variability**	No important uncertainty or variability			
BALANCE OF EFFECTS	Favors the comparison	Probably favors the comparison	Does not favor either the intervention or the comparison	Probably favors the intervention	**Favors the intervention**	Varies	Don't know
RESOURCES REQUIRED	Large costs	Moderate costs	**Negligible costs and savings**	Moderate savings	Large savings	Varies	Don't know
CERTAINTY OF EVIDENCE OF REQUIRED RESOURCES	Very low	Low	Moderate	High			**No included studies**
COST EFFECTIVENESS	Favors the comparison	Probably favors the comparison	Does not favor either the intervention or the comparison	Probably favors the intervention	Favors the intervention	Varies	**No included studies**
EQUITY	Reduced	Probably reduced	Probably no impact	**Probably increased**	Increased	Varies	Don't know
ACCEPTABILITY	No	Probably no	Probably yes	**Yes**		Varies	Don't know
FEASIBILITY	No	Probably no	Probably yes	**Yes**		Varies	Don't know

TYPE OF RECOMMENDATION

Strong recommendation against the intervention	Conditional recommendation against the intervention	Conditional recommendation for either the intervention or the comparison	**Conditional recommendation for the intervention**	Strong recommendation for the intervention

○	○	○	●	○

CONCLUSIONS

Recommendation

The panel suggests using the omphalocele circumference/abdominal circumference (OC/AC) ratio (measured from the 2nd trimester up until 32 weeks gestation) as a prognostic factor in combination with other prenatal measurements (e.g. extracorporeal (part/whole) liver herniation). This is preferably done in a research or registry setting as prospective data collection is warranted to further confirm the reliability of this measurement for predicting neonatal outcome.

Justification

Balance of effects

The panel members agree that the balance of effects is likely in favour of measuring one or more omphalocele ratio. As omphalocele is a birth defect with possibly severe consequences, collecting as much information as possible before counselling is likely beneficial to all stakeholders. The difference between OD/AC and OC/AC is discussed and as some panel members indicated that OD/AC probably has a smaller risk for measuring errors, the available evidence for OC/AC is stronger. In a previous study by Peters et al. (2014) both ratios were compared and OC/AC seemed more reliable as a prognostic factor.

Certainty of evidence

The current certainty about the reliability of OC/AC as a prognostic factor withheld the panel from issuing a strong recommendation.

Subgroup considerations

The panel did not formulate any specific subgroup considerations.

Implementation considerations

To gain insight into current practices pertaining to the management of omphalocele in Europe, a baseline survey has been conducted amongst centers involved in the European Reference Network for rare Inherited Congenital Anomalies (ERNICA) and/or connected to the European Pediatric Surgical Audit (EPSA). Center representatives are asked whether their center assesses the OC/AC ratio prenatally, as well as whether omphalocele size and/or ratios are

used as a prognostic factor (amongst other factors) for postnatal outcome, either for the purpose of counselling or care planning. Opportunities are provided for further explanation. If omphalocele size and/or ratios are used as a prognostic factor, the trimester (1st/2nd) in which omphalocele size and/or ratios are assessed is questioned.

ERNICA can play a role in promoting and facilitating opportunities for prospective data collection, through multicenter research initiatives and via the EPSA. ERNICA may also help facilitate educational activities on prenatal assessment. The panel noted that to measure the OC/AC ratio, maternal and fetal medicine specialists will require appropriate training. ERNICA may play a role in facilitating and supporting such training initiatives.

Monitoring and evaluation

We intend to employ the European Pediatric Surgical Audit (EPSA) as a continuous feedback mechanism to monitor and evaluate (recommendation-specific) implementation success. The EPSA is an international prospective clinical audit registry. (The need for) supplementary measures for validation purposes will also be explored.

Since prenatal data is often stored in the mother's file (which is often not accessible to pediatric surgeons), efforts to promote coordinated patient registration on a local level are required. The ERNICA/EPSA coordination teams will strive to facilitate initiatives to support local teams with such efforts.

Research priorities

Prospective data collection showing the relationship between OC/AC ratio and neonatal outcome is desirable. Strict instructions for timing of measurement, amount of repeat measurements and definitions for neonatal outcomes is imperative to establish higher certainty of evidence. Including this in the revision of the EPSA data set is a possibility, although there are barriers to the accurate registration of prenatal data as this is often included in the mother's medical file only. Additional research on the prognostic value of other omphalocele ratios is also warranted.

REFERENCES SUMMARY

Chock et al.(2019) (reference 15)

Montero et al.(2011) (reference 18)

Fawley et al. (2016) (reference 20)

Kiyohara et al.(2014) (reference 21)

Kleinrouweler et al. (2011) (reference 22)

Nitsche et. al. (2021) (reference 23)

Peters et al.(2019) (reference 24)

Peters et al. (2014) (reference 25)

QUESTION 1.3C

Should O/E lung volume measured with MRI be used for predicting neonatal outcomes?	
**POPULATION:**	Predicting neonatal outcomes
**INTERVENTION:**	O/E lung volume measured with MRI
**COMPARISON:**	
**MAIN OUTCOMES:**	Mortality and morbidity

ASSESSMENT

Desirable Effects
How substantial are the desirable anticipated effects?

JUDGEMENT	RESEARCH EVIDENCE	ADDITIONAL CONSIDERATIONS
O Trivial<br>O Small<br>O Moderate<br>● Large<br>O Varies<br>O Don't know	For the factor observed-to-expected total lung volume (O/E TLV) measured with prenatal MRI, three studies were included (Gallager et al. 2023, Dadoun et al. 2024 and Danzer et al. 2021). The literature was analyzed for outcomes: mortality, need for intubation, need for tracheostomy and length of hospital stay. Based on the analysis of these studies it seems likely there is a significant negative association between the O/E TLV <50% and survival and that O/E TLV predicts the need for intubation and ventilation as well as longer ventilation times. There are also indications that lower O/E TLV correlates with a higher chance of needing a tracheostomy. The results for length of stay were conflicting between studies so the panel concluded that it is unclear if O/E TLV could be predictive of length of stay.	Panel discussion<br><br>The panel concluded that measuring O/E TLV possibly has large benefits. The information that can be collected with a prenatal MRI cannot be obtained otherwise such as with ultrasound. Panel members agree that this information is helpful in the evaluation of prognosis, could increase the quality of counselling and could be helpful for neonatologists to optimally prepare care for a patient.

Undesirable Effects

How substantial are the undesirable anticipated effects?

JUDGEMENT	RESEARCH EVIDENCE	ADDITIONAL CONSIDERATIONS
O Trivial<br>● Small<br>O Moderate<br>O Large<br>O Varies<br>O Don't know		<u>Panel discussion</u><br><br>The measurement of O/E lung volume requires the patient to undergo another test in the hospital. However, the panel concluded that the possible undesirable effects of such a test are small. As data is still not 100% accurate, using this information in the counselling could lead to a more negative scenario but prenatal specialists estimate this chance to be small. Thereby, the optimal cut-off is not clear from the current research. Most studies used <50% as a cut-off related to worse outcomes. Danzer et al. 2021 grouped patients into three groups: <25%, 25-50% and >50%. As the number of patients in the <25% group was very small (n=5): no strong conclusions could be drawn. Panel members also emphasized that in the included studies, MRI scans were taken mainly in the third trimester. As we don't know the reliability of earlier measurements, O/E TLV may not be suitable as an early predictor of prognosis for parents that consider termination of pregnancy.

Certainty of evidence

What is the overall certainty of the evidence of effects?

JUDGEMENT	RESEARCH EVIDENCE	ADDITIONAL CONSIDERATIONS
O Very low<br>● Low<br>O Moderate<br>O High<br>O No included studies	The overall quality of evidence was rated as low. This is mainly due to the fact that all included studies are downrated for a risk of bias due to their retrospective set-up and the lack of correction for important confounders. It is unknown if there were significant baseline differences between survivors and non-survivors or high and low O/E TLV. There are also concerns of selection bias in all included studies as none of them included a clear definition of giant omphalocele. The defect size was reported as well as liver herniation in Dadoun 2024 and Gallager 2023 but no clear cut-offs were used. Danzer et al.	

	2021 refer for inclusion to their institutional definition which is unspecified.	

Values

Is there important uncertainty about or variability in how much people value the main outcomes?

JUDGEMENT	RESEARCH EVIDENCE	ADDITIONAL CONSIDERATIONS
O Important uncertainty or variability<br>O Possibly important uncertainty or variability<br>● Probably no important uncertainty or variability<br>O No important uncertainty or variability		Panel discussion<br><br>The panel concluded that there are likely no important differences in how stakeholders value the outcomes. Parents would probably want to know how likely it is that their child will fall into a certain category, for example how likely it is that their child will need ventilation. Lung volume may even be an outcome that is less abstract for parents compared to other consequences of the omphalocele. Clinicians will benefit from this information in their preparation for the perinatal care of the patient.

Balance of effects

Does the balance between desirable and undesirable effects favor the intervention or the comparison?

JUDGEMENT	RESEARCH EVIDENCE	ADDITIONAL CONSIDERATIONS
○ Favors the comparison<br>○ Probably favors the comparison<br>○ Does not favor either the intervention or the comparison<br>○ Probably favors the intervention<br>● Favors the intervention<br>○ Varies<br>○ Don't know		<u>Panel discussion</u><br><br>The panel concluded that the balance of effects probably favors including O/E lung volume as a prognostic factor. A prenatal MRI can obtain information on the lung condition that cannot be obtained via ultrasound. As the certainty of evidence for the conclusions is still low, panel members do advise caution in the interpretation and measurement as part of prospective data collection.

Resources required

JUDGEMENT	RESEARCH EVIDENCE	ADDITIONAL CONSIDERATIONS
○ Large costs<br>● Moderate costs<br>○ Negligible costs and savings<br>○ Moderate savings<br>○ Large savings<br>○ Varies<br>○ Don't know		<u>Panel discussion</u><br><br>The panel expects a moderate increase in costs per patient. This relates mainly to the use of the MRI.

Certainty of evidence of required resources

What is the certainty of the evidence of resource requirements (costs)?

JUDGEMENT	RESEARCH EVIDENCE	ADDITIONAL CONSIDERATIONS
O Very low<br>O Low<br>O Moderate<br>O High<br>● No included studies		

Cost effectiveness

Does the cost-effectiveness of the intervention favor the intervention or the comparison?

JUDGEMENT	RESEARCH EVIDENCE	ADDITIONAL CONSIDERATIONS
O Favors the comparison<br>O Probably favors the comparison<br>O Does not favor either the intervention or the comparison<br>O Probably favors the intervention<br>O Favors the intervention<br>O Varies<br>● No included		

studies		

Equity
What would be the impact on health equity?

JUDGEMENT	RESEARCH EVIDENCE	ADDITIONAL CONSIDERATIONS
O Reduced<br>O Probably reduced<br>O Probably no impact<br>● Probably increased<br>O Increased<br>O Varies<br>O Don't know		Panel discussion<br><br>The panel concluded that measuring this prognostic factor probably results in increased care equity as it could allow for early referral to expert care centers, for a prenatal MRI and counselling in a fetal medicine unit.

Acceptability
Is the intervention acceptable to key stakeholders?

JUDGEMENT	RESEARCH EVIDENCE	ADDITIONAL CONSIDERATIONS
O No<br>O Probably no<br>● Probably yes<br>O Yes<br>O Varies		Panel discussion<br><br>The panel does not see any important barriers to acceptability.

○ Don't know		

Feasibility
Is the intervention feasible to implement?

JUDGEMENT	RESEARCH EVIDENCE	ADDITIONAL CONSIDERATIONS
○ No<br>○ Probably no<br>○ Probably yes<br>● Yes<br>○ Varies<br>○ Don't know		<u>Panel discussion</u><br><br>The panel does not see any important barriers to feasibility. As the number of patients that will need a prenatal MRI for omphalocele is so small, no large increase in scans is expected with the recommendation. All ERNICA centers either have access to a prenatal MRI in their own center, or can refer to a maternal and fetal medicine unit within the network.

SUMMARY OF JUDGEMENTS

	JUDGEMENT						
**PROBLEM**	No	Probably no	Probably yes	Yes		Varies	Don't know
**DESIRABLE EFFECTS**	Trivial	Small	Moderate	**Large**		Varies	Don't know
**UNDESIRABLE EFFECTS**	Trivial	**Small**	Moderate	Large		Varies	Don't know
**CERTAINTY OF EVIDENCE**	Very low	**Low**	Moderate	High			No included studies
**VALUES**	Important uncertainty or variability	Possibly important uncertainty or variability	**Probably no important uncertainty or variability**	No important uncertainty or variability			
**BALANCE OF EFFECTS**	Favors the comparison	Probably favors the comparison	Does not favor either the	Probably favors the intervention	**Favors the intervention**	Varies	Don't know

	JUDGEMENT						
			intervention or the comparison				
RESOURCES REQUIRED	Large costs	**Moderate costs**	Negligible costs and savings	Moderate savings	Large savings	Varies	Don't know
CERTAINTY OF EVIDENCE OF REQUIRED RESOURCES	Very low	Low	Moderate	High			**No included studies**
COST EFFECTIVENESS	Favors the comparison	Probably favors the comparison	Does not favor either the intervention or the comparison	Probably favors the intervention	Favors the intervention	Varies	**No included studies**
EQUITY	Reduced	Probably reduced	Probably no impact	**Probably increased**	Increased	Varies	Don't know
ACCEPTABILITY	No	Probably no	**Probably yes**	Yes		Varies	Don't know
FEASIBILITY	No	Probably no	Probably yes	**Yes**		Varies	Don't know

TYPE OF RECOMMENDATION

Strong recommendation against the intervention	Conditional recommendation against the intervention	Conditional recommendation for either the intervention or the comparison	**Conditional recommendation for the intervention**	Strong recommendation for the intervention
○	○	○	●	○

CONCLUSIONS

Recommendation

The panel recommends a prenatal MRI for lung volume evaluation early in the 3rd trimester in case of suspected giant omphalocele.

The panel suggests using Observed-to-Expected (O/E) total lung volume (TLV) calculated with fetal MRI according to the Meyers nomogram in prenatal counselling (1). An O/E <50% seems to be associated with a higher risk of mortality and morbidity. Assessment of such prognostic factors is preferably done in a research or registry setting, as prospective data collection is warranted to confirm the reliability of these measurements for predicting neonatal outcome

Justification

Subgroup considerations

The recommendations in this chapter are specifically for fetuses with suspected giant omphalocele. The panel agrees that a prenatal MRI does not add valuable information for fetuses with a small omphalocele as lung volume is usually unaffected in this group.

Implementation considerations

To gain insight into current practices pertaining to the management of omphalocele in Europe, a baseline survey has been conducted amongst centers involved in the European Reference Network for rare Inherited Congenital Anomalies (ERNICA) and/or connected to the European Pediatric Surgical Audit (EPSA). Center representatives are asked whether their center assesses total lung volume on fetal MRI pre-natally. If they answer ‘nearly always’, ‘Sometimes/it depends’ or ‘rarely’, representatives are asked whether the decision to perform such an assessment depends on whether giant omphalocele is expected. Possibilities for further explanations are provided. Centers are also asked whether they use low total lung volume on fetal MRI as a prognostic outcome for the purpose of prenatal counselling/care planning.

ERNICA can play a role in promoting and facilitating opportunities for prospective data collection, through multicenter research initiatives and via the EPSA. ERNICA may also help facilitate educational activities on prenatal assessment and play a role in facilitating access to other maternal and fetal medicine units within the network (should a center not have access to prenatal MRI).

Monitoring and evaluation

We intend to employ the European Pediatric Surgical Audit (EPSA) as a continuous feedback mechanism to monitor and evaluate (recommendation-specific) implementation success. The EPSA is an international prospective clinical audit registry. (The need for) supplementary measures for validation purposes will also be explored.

Since prenatal data is often stored in the mother's file (which is often not accessible to pediatric surgeons), efforts to promote coordinated patient registration on a local level are required. The ERNICA/EPSA coordination teams will strive to facilitate initiatives to support local teams with such efforts.

Research priorities

To further increase the quality of counselling based on prenatal MRI data, more insights are imperative. The panel prioritizes prospective data collection in a research or registry setting, where the definition of giant omphalocele used for inclusion is uniform. Including this in the revision of the EPSA data set is a possibility, although there are barriers to the accurate registration of prenatal data as this is often included in the mother's medical file only. Additionally, the exploration of differences between severity classifications such as cut-off <25%, 25-50% and >50% and assessment of the prognostic value of second trimester MRI is desirable.

REFERENCES SUMMARY

Dadoun et al. 2024 (reference 28)

Danzer et al. 2021 (reference 29)

Gallager et al. 2023 (reference 30)

1. Meyers ML, Garcia JR, Blough KL, Zhang W, Cassady CI, Mehollin Ray AR. Fetal lung volumes by MRI: normal weekly values from 18 through 38 weeks' gestation. AJR Am J Roentgenol. 2018;211: 432-438.

QUESTION 2.1

Should pre-operative feeding vs. post-operative feeding be used for patients with omphalocele that receive staged reduction?	
**POPULATION:**	Patients with omphalocele that receive staged reduction
**INTERVENTION:**	Pre-operative feeding
**COMPARISON:**	Post-operative feeding
**MAIN OUTCOMES:**	Time to full feeding, length of stay, postoperative complications

ASSESSMENT

Problem

Is the problem a priority?

JUDGEMENT	RESEARCH EVIDENCE	ADDITIONAL CONSIDERATIONS
o No<br>o Probably no<br>o Probably yes<br>o Yes<br>o Varies<br>o Don't know		

Desirable Effects

How substantial are the desirable anticipated effects?

JUDGEMENT	RESEARCH EVIDENCE	ADDITIONAL CONSIDERATIONS
o Trivial<br>o Small<br>o Moderate<br>o Large<br>o Varies<br>● Don't know	No published evidence was found to answer this question.	EPSA summary:<br><br>Time to full feeds: Majority of panel members says **no effect** (10/19) or indicates missing information (9/19). The panel members that indicate missing information declare that they mainly miss the motivation behind when enteral feeding was started and that this would help them interpret the data better.

		Length of postoperative hospital stay: The majority of the panel indicated **small or moderate harm**. (12/19). Other answers were no effect (3/19) and don't know (4/19). The panel members agree that their observation of a longer length of postoperative hospital stay may be the effect of a confounder, as the 'early feeding group' has a higher rate of pulmonary complications. Their collective judgement would therefore be **no effect.**<br><br>Post operative complications: A part of the panel members says **no effect** (8/19), others indicate missing information (7/19). Other answers were small harm (4/19).<br><br><u>Panel discussion</u><br><br>Panel members agreed that in general, earlier feeding is preferable as it may be related to the prevention of PN /central line related complications, oral aversion or atrophy. However, the condition of the baby will not always allow start of enteral feeding early, such as in cases of bowel obstruction. As this information is not recorded, interpretation of the EPSA data for these outcomes is difficult. Panel members do find it remarkable that in the pre-operative feeding group, the average time to surgery was much longer than in the post-operative feeding group. Time to start of enteral feeding could not be interpreted due to missing data, but counted in days, this could be similar for both groups. All things considered, panel members agree that the fact that no clear negative effects of pre-operative feeding were observed, does indicate that pre-operative feeding is probably safe. Some evidence from other malformations such as gastroschisis, suggests that earlier feeding is probably related to some benefits (1-3).

Undesirable Effects

How substantial are the undesirable anticipated effects?

JUDGEMENT	RESEARCH EVIDENCE	ADDITIONAL CONSIDERATIONS

○ Trivial<br>● Small<br>○ Moderate<br>○ Large<br>○ Varies<br>○ Don't know		Panel discussion<br><br>Panel members agreed that delay of enteral feeding could be related to a higher chance of central line complications or other complications related to parenteral nutrition (PN). We did not find evidence for this relationship, but panel members agree that it is generally known that the risk for these complications rises simply with longer exposure. As a possible undesirable effect of pre-operative feeding, the risk of reflux due to abdominal compression is mentioned by some of the neonatologists. This could cause micro-aspiration and pulmonary hypertension, and both should be avoided at all times. With close monitoring and gradual, cautious introduction of enteral feeding, panel members estimate this risk as small.

Certainty of evidence

What is the overall certainty of the evidence of effects?

JUDGEMENT	RESEARCH EVIDENCE	ADDITIONAL CONSIDERATIONS
○ Very low<br>○ Low<br>○ Moderate<br>○ High<br>● No included studies		EPSA data<br><br>The lack of an intention to treat in the EPSA data makes the certainty panel members can attach to their conclusions lower.

Values

Is there important uncertainty about or variability in how much people value the main outcomes?

JUDGEMENT	RESEARCH EVIDENCE	ADDITIONAL CONSIDERATIONS
○ Important uncertainty or variability<br>○ Possibly important uncertainty or variability		Panel discussion<br><br>Panel members agreed that there is probably no difference in how outcomes are valued. Although the outcome of time to full feeding is important to clinicians (prevention of PN complications) parents might value this outcome even more strongly as feeding their child is associated with normality. Both our patient representatives strongly agreed with this. One of

● Probably no important uncertainty or variability<br>○ No important uncertainty or variability		our patient representatives indicated that being able to start tube feeding with breast milk gives the mother an important role in the recovery of her child, something that is desirable.

Balance of effects

Does the balance between desirable and undesirable effects favor the intervention or the comparison?

JUDGEMENT	RESEARCH EVIDENCE	ADDITIONAL CONSIDERATIONS
○ Favors the comparison<br>○ Probably favors the comparison<br>○ Does not favor either the intervention or the comparison<br>○ Probably favors the intervention<br>● Favors the intervention<br>○ Varies<br>○ Don't know		Panel discussion<br><br>Panel members agree that in most patients, starting enteral feeding as early as possible, even before closure of the abdominal wall, is beneficial, as prolonging parenteral nutrition (PN) may increase the risk of complications related to the PN or central line or oral aversion. Starting enteral feeding in patients with an open abdominal wall (during staged reduction) should be done gradually and carefully, with attendance to tolerance of the feeding by the patient to avoid or reduce reflux and gastric retention.

Resources required

JUDGEMENT	RESEARCH EVIDENCE	ADDITIONAL CONSIDERATIONS

○ Large costs<br>○ Moderate costs<br>○ Negligible costs and savings<br>● Moderate savings<br>○ Large savings<br>○ Varies<br>○ Don't know	Breastmilk or powder milk is much cheaper than PN so earlier feeding could lead to savings on a patient level.	Panel discussion<br>Breast milk or formula are much cheaper compared to PN.

Certainty of evidence of required resources

What is the certainty of the evidence of resource requirements (costs)?

JUDGEMENT	RESEARCH EVIDENCE	ADDITIONAL CONSIDERATIONS
○ Very low<br>○ Low<br>○ Moderate<br>○ High<br>●No included studies		

Cost effectiveness

Does the cost-effectiveness of the intervention favor the intervention or the comparison?

JUDGEMENT	RESEARCH EVIDENCE	ADDITIONAL CONSIDERATIONS

o Favors the comparison<br>o Probably favors the comparison<br>o Does not favor either the intervention or the comparison<br>o Probably favors the intervention<br>o Favors the intervention<br>o Varies<br>● No included studies		

Equity

What would be the impact on health equity?

JUDGEMENT	RESEARCH EVIDENCE	ADDITIONAL CONSIDERATIONS
o Reduced<br>o Probably reduced<br>● Probably no impact<br>o Probably increased<br>o Increased<br>o Varies<br>o Don't know		<u>Panel discussion</u><br><br>Panel members agreed that any recommendations made as to the timing of introducing enteral feeding will have little to no impact on care equity.

Acceptability

Is the intervention acceptable to key stakeholders?

JUDGEMENT	RESEARCH EVIDENCE	ADDITIONAL CONSIDERATIONS
o No<br>o Probably no<br>● Probably yes<br>o Yes		<u>Panel discussion</u><br><br>Panel members agreed that a recommendation for earlier feeding (before closure of the abdominal wall, during staged reduction) is probably acceptable to key stakeholders (clinicians and patients/parents). Despite understanding the potential benefits of trying early feeding, one

o Varies<br>o Don't know		barrier to acceptability may be that some surgeons prefer/find it easier to work on patients with an empty stomach. It is very likely that parents/caregivers will prefer a recommendation for earlier feeding as they often associate feeding their child with normality. Being able to provide breastmilk as a food source is often highly valued by mothers.

Feasibility
Is the intervention feasible to implement?

JUDGEMENT	RESEARCH EVIDENCE	ADDITIONAL CONSIDERATIONS
o No<br>o Probably no<br>o Probably yes<br>● Yes<br>o Varies<br>o Don't know		Panel discussion<br><br>Panel members consider the recommendation feasible. Changing the time that enteral feeding starts does not require any additional resources or training.

SUMMARY OF JUDGEMENTS

	JUDGEMENT						
PROBLEM	No	Probably no	Probably yes	Yes		Varies	Don't know
DESIRABLE EFFECTS	Trivial	Small	Moderate	Large		Varies	**Don't know**
UNDESIRABLE EFFECTS	Trivial	**Small**	Moderate	Large		Varies	Don't know
CERTAINTY OF EVIDENCE	Very low	Low	Moderate	High			**No included studies**
VALUES	Important uncertainty or variability	Possibly important uncertainty or variability	**Probably no important uncertainty or variability**	No important uncertainty or variability			
BALANCE OF EFFECTS	Favors the comparison	Probably favors the comparison	Does not favor either the intervention or the comparison	Probably favors the intervention	**Favors the intervention**	Varies	Don't know

	JUDGEMENT						
RESOURCES REQUIRED	Large costs	Moderate costs	Negligible costs and savings	**Moderate savings**	Large savings	Varies	Don't know
CERTAINTY OF EVIDENCE OF REQUIRED RESOURCES	Very low	Low	Moderate	High			**No included studies**
COST EFFECTIVENESS	Favors the comparison	Probably favors the comparison	Does not favor either the intervention or the comparison	Probably favors the intervention	Favors the intervention	Varies	**No included studies**
EQUITY	Reduced	Probably reduced	**Probably no impact**	Probably increased	Increased	Varies	Don't know
ACCEPTABILITY	No	Probably no	**Probably yes**	Yes		Varies	Don't know
FEASIBILITY	No	Probably no	Probably yes	**Yes**		Varies	Don't know

TYPE OF RECOMMENDATION

Strong recommendation against the intervention	Conditional recommendation against the intervention	Conditional recommendation for either the intervention or the comparison	Conditional recommendation for the intervention	**Strong recommendation for the intervention**
○	○	○	○	●

CONCLUSIONS

Recommendation

The panel recommends a pre-operative start of (at least trophic) enteral feeding in patients that undergo staged reduction.

Justification

Balance of effects

Panel members agree that in most patients, starting enteral feeding as early as possible, even before closure of the abdominal wall, is beneficial, as prolonging parenteral nutrition (PN) may increase the risk of complications related to the PN or central line or oral aversion.

Values

It is very likely that parents/caregivers will prefer a recommendation for earlier feeding as they often associate feeding their child with normality. Being able to provide breastmilk as a food source is often highly valued by mothers.

Subgroup considerations

Considerations to stop pre-operative enteral feeding or to exclude patients from the recommendation:

The panel recommends practising the recommendation as long as it is tolerated by the patient and stop or refrain from enteral feeding in case of reflux or gastric retention. It is of note that the correct evaluation of tolerance of enteral feeding is unknown and that current practices regarding this evaluation vary between care providers.

Implementation considerations

To gain insight into current practices pertaining to the management of omphalocele in Europe, a baseline survey has been conducted amongst centers involved in the European Reference Network for rare Inherited Congenital Anomalies (ERNICA) and/or connected to the European Pediatric Surgical Audit (EPSA). Center representatives are asked whether their center starts enteral feeding before or after closure of the abdominal wall, or whether this 'depends'. An opportunity is provided for further explanation. In the case of an 'It depends' response, respondents are asked to specify when they start and/or what parameters they use to determine optimal timing to start enteral feeding.

A qualitative study will also take place to further explore the factors foreseen to hinder/facilitate successful implementation of this recommendation in clinical practice. This will be done with a view to collaboratively selecting implementation strategies.

It may be that some centers use gastric retention as an indication for feeding – this could be a barrier to implementing the recommendation.

Monitoring and evaluation

We intend to employ the European Pediatric Surgical Audit (EPSA) as a continuous feedback mechanism to monitor and evaluate (recommendation-specific) implementation success. The EPSA is an international prospective clinical audit registry. (The need for) supplementary measures for validation purposes will also be explored.

Research priorities

Panel members agree that there is a lack of knowledge on several aspects related to the feeding practices in omphalocele patients. One of those is that it is unknown if there is a correlation between time to enteral feeding and growth of the patient. This could be explored by prospectively measuring time to enteral feeding and growth of patients. Comparing patients on parenteral nutrition and on full enteral nutrition at 28 days postoperatively in regards to their growth z-scores for example could provide useful information. Another important knowledge gap is the exact meaning of staring enteral feeding. Panel members think it would be beneficial to find out with what ratio of enteral feeding/nutrition via bowel possible benefits can be expected. Are some of these benefits also present for example with trophic feeds (minimal enteral feeding or non-nutritive feeding)? Last, panel members mention the lack of knowledge about the differences of enteral feeding via nasogastric tube feeds and oral feeds in these patients.

REFERENCES SUMMARY

1.Dama M, Rao U, Gollow I, Bulsara M, Rao S. Early commencement of enteral feeds in gastroschisis: a systematic review of literature. Eur J Pediatr Surg. 2017 Dec;27(6):503-515. doi: 10.1055/s-0037-1598086. epub 2017 Jan 23. PMID: 28114704.

2.Lemoine JB, Smith RR, White D. Got milk? Effects of early enteral feedings in patients with gastroschisis. Adv Neonatal Care. 2015 Jun;15(3):166-75. doi: 10.1097/anc.0000000000000171. PMID: 25938951.

3.Aljahdali A, Mohajerani N, Skarsgard ED; Canadian Pediatric Surgery Network (CAPSNet). Effect of timing of enteral feeding on outcome in gastroschisis. J Pediatr Surg. 2013 May;48(5):971-6. doi: 10.1016/j.jpedsurg.2013.02.014. PMID: 23701769.

QUESTION 2.2

**Should spontaneous breathing vs. intubation and mechanical ventilation be used for patients with omphalocele that receive staged reduction?**	
**POPULATION:**	Patients with omphalocele that receive staged reduction
**INTERVENTION:**	Spontaneous breathing
**COMPARISON:**	Intubation and mechanical ventilation
**MAIN OUTCOMES:**	Length of stay, time to full feeding, management of pain/patient comfort

ASSESSMENT

Problem<br>Is the problem a priority?		
JUDGEMENT	RESEARCH EVIDENCE	ADDITIONAL CONSIDERATIONS
O No<br>O Probably no<br>O Probably yes<br>O Yes<br>O Varies<br>O Don't know		
**Desirable Effects**<br>How substantial are the desirable anticipated effects?		
JUDGEMENT	RESEARCH EVIDENCE	ADDITIONAL CONSIDERATIONS
O Trivial<br>O Small	No published evidence was found to answer this question.	EPSA summary<br>Three outcomes could be evaluated from the EPSA data.

○ Moderate<br>○ Large<br>○ Varies<br>○ Don't know		**Days until surgery:** Most panel members indicated '**No effect'** (12) Don't know (6) Large benefit (1).<br><br>**Length of postoperative hospital stay:** Most panel members (14) indicated **small or moderate harm,** to their observation, the non-ventilated patients had longer post-operative hospital stays. Others observed no effect (1) or indicated Don't know (4)<br><br>**Time to full feeds:** Most panel members indicated **Large/small benefit** (14), to their observation, the awake patients reached full feeding earlier. Some panel members indicated Don't know (4) or No effect (1).<br><br><u>Panel discussion</u><br><br>Panel members discussed the value of these findings from the structured observations forms. Their collective opinion is that for this question, the EPSA data have limited added value due to numerous reasons:<br><br>- Outcomes do not point in the same direction, panel members observe some harms for unventilated patients as it comes to post-operative hospital stay, but some benefits as it comes to time to full feeds.<br>- We don't know whether patients were kept on ventilation due to respiratory needs or due to surgeons' preference/choice<br>- Whether or not it is feasible to do staged reduction with the patient awake also depends on the chosen intervention. With some interventions, like a silo or taping, it may be very well possible whilst with other interventions like suture traction or Fasciotens it is not. As we don't know the exact type of staged reduction, data is difficult to interpret.<br>- The groups are too small (9 awake versus 12 ventilated patients with available data on outcomes).<br><br>As the intention to treat is unclear from the data, the methodologist asked the panel members about their considerations as to intubation. Based on their expert opinion, panel members expressed that intubation and mechanical ventilation makes the patient completely relax their muscle tone and that this could ease the reductions of the content into the abdominal cavity. Some experts report giving muscle relaxants in awake

		babies, but we have no data to evaluate the effect of this practice. A second benefit is that intubation and mechanical ventilation allows for more options to control the patient's pain and keep them comfortable.

Undesirable Effects

How substantial are the undesirable anticipated effects?

JUDGEMENT	RESEARCH EVIDENCE	ADDITIONAL CONSIDERATIONS
O Trivial<br>O Small<br>O Moderate<br>O Large<br>O Varies<br>O Don't know		<u>Panel discussion</u><br><br>The panel members discussed that in general, there are considerable undesirable effects of (prolonged) intubation and mechanical ventilation. Even though no evidence specifically for omphalocele was found, data from other populations (premature patients, patients with congenital diaphragmatic hernia) suggests that mechanical ventilation leads to higher risk of complications such as lung damage, respiratory infections, tube obstruction or even pneumothorax(1-4). Thereby, patients need to be under general anaesthesia, and this can have negative side effects on its own in the developing child, especially for those under the age of one year old. Giving the patients sedation or anaesthesia during intubation could also negatively impact the chances for early enteral feeding.

Certainty of evidence<br>What is the overall certainty of the evidence of effects?		
JUDGEMENT	RESEARCH EVIDENCE	ADDITIONAL CONSIDERATIONS
○ Very low<br>○ Low<br>○ Moderate<br>○ High<br>● No included studies		EPSA data<br><br>The lack of an intention to treat, the lack of data about the exact intervention and the small samples that could be analysed from the EPSA data makes the certainty panel members can attach to their conclusions very low.

Values<br>Is there important uncertainty about or variability in how much people value the main outcomes?		
JUDGEMENT	RESEARCH EVIDENCE	ADDITIONAL CONSIDERATIONS
○ Important uncertainty or variability<br>○ Possibly important uncertainty or variability<br>● Probably no important uncertainty or variability<br>○ No important uncertainty or variability		Panel discussion<br><br>Panel members don't think there are large differences in how much stakeholders value the discussed outcomes. For both clinicians and parents, feeding outcomes are considered important as they can be a proxy for the wellbeing of the child and for parents, feeding their child is often associated with normality. Our patient representatives expressed that assuring their child is not in pain is a very important outcome to them.

Balance of effects

Does the balance between desirable and undesirable effects favor the intervention or the comparison?

JUDGEMENT	RESEARCH EVIDENCE	ADDITIONAL CONSIDERATIONS
O Favors the comparison<br>O Probably favors the comparison<br>O Does not favor either the intervention or the comparison<br>O Probably favors the comparison<br>O Probably favors the intervention<br>O Favors the intervention<br>● Varies<br>O Don't know		Panel discussion<br><br>Panel members agreed that the balance of effects will be different for the different staged interventions and that it is not desirable to give an overarching judgement on the balance of effects in this case.

Resources required

JUDGEMENT	RESEARCH EVIDENCE	ADDITIONAL CONSIDERATIONS
O Large costs<br>O Moderate costs<br>O Negligible costs and savings<br>O Moderate savings<br>O Large savings<br>● Varies<br>O Don't know		Panel discussion<br><br>Panel members agree that the required resources for ventilation are probably higher than for awake treatment, but because the balance of effects will be different for the different staged interventions, it is not desirable to give an overarching judgement on the required resources in this case.

Certainty of evidence of required resources<br>What is the certainty of the evidence of resource requirements (costs)?		
JUDGEMENT	RESEARCH EVIDENCE	ADDITIONAL CONSIDERATIONS
O Very low<br>O Low<br>O Moderate<br>O High<br>● No included studies		

Cost effectiveness<br>Does the cost-effectiveness of the intervention favor the intervention or the comparison?		
JUDGEMENT	RESEARCH EVIDENCE	ADDITIONAL CONSIDERATIONS
O Favors the comparison<br>O Probably favors the comparison<br>O Does not favor either the intervention or the comparison<br>O Probably favors the intervention<br>O Favors the intervention<br>O Varies<br>● No included studies		

Equity

What would be the impact on health equity?

JUDGEMENT	RESEARCH EVIDENCE	ADDITIONAL CONSIDERATIONS
○ Reduced<br>○ Probably reduced<br>○ Probably no impact<br>● Probably increased<br>○ Increased<br>○ Varies<br>○ Don't know		<u>Panel discussion</u><br><br>The panel agreed that if the option of awake reduction is at least considered by surgical teams and parents, this could probably increase equity for patients who receive the same intervention (for example silo) in different hospitals/countries.

Acceptability

Is the intervention acceptable to key stakeholders?

JUDGEMENT	RESEARCH EVIDENCE	ADDITIONAL CONSIDERATIONS
○ No<br>● Probably no<br>○ Probably yes<br>○ Yes<br>○ Varies<br>○ Don't know	Barriers: differences of opinion and current practices. For some panel members they would not be comfortable to reduce a silo with an awake baby.	<u>Panel discussion</u><br><br>The panel foresees some important barriers. First, even within the panel group, there are differences in opinion and there is known variability in local practices, even when it comes to using the same intervention for staged reduction.<br><br>Some surgical panel members indicated that they would keep patients with a silo awake, yet others would not feel comfortable with this practice. Surgeons and neonatologists also can have different opinions and preferences.<br><br>As there is no evidence (on the chosen outcomes) to support one option over the other, it is foreseen that it would be difficult to convince surgical teams to change their practices.<br><br>Panel members do agree that awake reduction could be considered, if the intervention allows for it and the patient appears comfortable. For parents of the patient, awake reduction would only be acceptable if they can be assured that their child is not in pain.

**Feasibility**<br>Is the intervention feasible to implement?		
JUDGEMENT	RESEARCH EVIDENCE	ADDITIONAL CONSIDERATIONS
○ No<br>● Probably no<br>○ Probably yes<br>○Yes<br>○ Varies<br>○ Don't know		<u>Panel discussion</u><br><br>Both options are widely available and possible. However, awake reduction would require substantial behavioral change in some teams. This may limit the feasibility.

SUMMARY OF JUDGEMENTS

	JUDGEMENT						
**PROBLEM**	No	Probably no	Probably yes	Yes		Varies	Don't know
**DESIRABLE EFFECTS**	Trivial	Small	Moderate	Large		Varies	Don't know
**UNDESIRABLE EFFECTS**	Trivial	Small	Moderate	Large		Varies	Don't know
**CERTAINTY OF EVIDENCE**	Very low	Low	Moderate	High			**No included studies**
**VALUES**	Important uncertainty or variability	Possibly important uncertainty or variability	Probably no important uncertainty or variability	No important uncertainty or variability			
**BALANCE OF EFFECTS**	Favors the comparison	Probably favors the comparison	Does not favor either the intervention or the comparison	Probably favors the intervention	Favors the intervention	**Varies**	Don't know

	JUDGEMENT						
RESOURCES REQUIRED	Large costs	Moderate costs	Negligible costs and savings	Moderate savings	Large savings	**Varies**	Don't know
CERTAINTY OF EVIDENCE OF REQUIRED RESOURCES	Very low	Low	Moderate	High			**No included studies**
COST EFFECTIVENESS	Favors the comparison	Probably favors the comparison	Does not favor either the intervention or the comparison	Probably favors the intervention	Favors the intervention	Varies	**No included studies**
EQUITY	Reduced	Probably reduced	Probably no impact	**Probably increased**	Increased	Varies	Don't know
ACCEPTABILITY	No	**Probably no**	Probably yes	Yes		Varies	Don't know
FEASIBILITY	No	Probably no	Probably yes	Yes		Varies	Don't know

TYPE OF RECOMMENDATION

Strong recommendation against the intervention	Conditional recommendation against the intervention	Conditional recommendation for either the intervention or the comparison	Conditional recommendation for the intervention	Strong recommendation for the intervention
○	○	●	○	○

CONCLUSIONS

Recommendation

The panel suggest choosing either intubation or spontaneous breathing during staged reduction, depending on the condition of the patient (incl. pain levels) and the method used for staged closure.

Justification

Undesirable effects

Downsides of ventilation are possibly large. Therefore, the panel members agree that if it is possible, awake reduction should at least be considered. In patients where it is possible, spontaneous breathing may avoid the possible consequences of ventilation and intubation. In these cases, it is probably acceptable to keep patients on spontaneous breathing (extubated) but at the moment there is too little evidence to prove superiority of this over ventilation for all patients. The undesirable effects may also depend on the chosen intervention for staged reduction.

Values

For parents of the patient, awake reduction would only be acceptable if they can be assured that their child is not in pain.

Subgroup considerations

If awake reduction is considered, patient representatives would like to introduce the following considerations:

- Explain to parents how pain and comfort levels are assessed (visual scales, measurement of respiratory rate/saturation).
- Explain the possibility of having to resort to ventilation at a later stage if the situation worsens
- Explain in advance the possibility of touching or cuddling, if the child is on a particular respiratory aid.

Implementation considerations

To gain insight into current practices pertaining to the management of omphalocele in Europe, a baseline survey has been conducted amongst centers involved in the European Reference Network for rare Inherited Congenital Anomalies (ERNICA) and/or connected to the European Pediatric Surgical Audit (EPSA). Center representatives are asked whether their center intubates giant omphalocele patients during staged reduction (Yes, always; nearly always; sometimes/it depends; rarely, never) and they have the option to provide an explanation for their answer. As an expertise network, ERNICA can play a role in faciliating the exchange of knowledge/experience on current practices.

Monitoring and evaluation

We intend to employ the European Pediatric Surgical Audit (EPSA) as a continuous feedback mechanism to monitor and evaluate center practices. The EPSA is an international prospective clinical audit registry.

Research priorities

To provide better answers in the future, prospective data collection including the specific type of intervention should be prioritized. As some panel members have the idea that reduction and closure of the abdominal wall can be done faster in ventilated patients, the correlation between ventilation and time to closure should be explored. Asides, prospective data collection could provide insight into the differences in complication rate and the number of complications related to intubation.

REFERENCES SUMMARY

1. Ing C, Vutskits L. Unanswered questions of anesthesia neurotoxicity in the developing brain. Curr Opin Anaesthesiol. 2023 Oct 1;36(5):510-515. doi: 10.1097/aco.0000000000001295. epub 2023 Jul 19. PMID: 37552011; PMCID: PMC10939468.

2.Keunen K, Sperna Weiland NH, de Bakker BS, de Vries LS, Stevens MF. Impact of surgery and anesthesia during early brain development: a perfect storm. Paediatr Anaesth. 2022 Jun;32(6):697-705. doi: 10.1111/pan.14433. epub 2022 Mar 16. PMID: 35266610; PMCID: PMC9311405.

3.Van Kaam AH. Optimal strategies of mechanical ventilation: can we avoid or reduce lung injury? Neonatology. 2024;121(5):570-575. doi: 10.1159/000539346. epub 2024 Jun 13. PMID: 38870922; PMCID: PMC11446299.

4.Kalikkot Thekkeveedu R, El-Saie A, Prakash V, Katakam L, Shivanna B. Ventilation-induced lung injury (VILI) in neonates: evidence-based concepts and lung-protective strategies. J Clin Med. 2022 Jan 22;11(3):557. doi: 10.3390/jcm11030557. PMID: 35160009; PMCID: PMC8836835

QUESTION 3.1

**Should conservative management (paint and/or wait) and delayed closure vs. staged surgical reduction be used for patients with giant omphalocele ?**	
**POPULATION:**	Patients with giant omphalocele
**INTERVENTION:**	Non-operative management (paint and/or wait) and delayed closure
**COMPARISON:**	Staged surgical reduction
**MAIN OUTCOMES:**	Mortality, Length of Stay, Time to full feeds, Post-operative complications, Neurodevelopment

ASSESSMENT

**Problem**<br>Is the problem a priority?		
JUDGEMENT	RESEARCH EVIDENCE	ADDITIONAL CONSIDERATIONS
O No<br>O Probably no<br>O Probably yes<br>O Yes<br>O Varies<br>O Don't know		
**Desirable Effects**<br>How substantial are the desirable anticipated effects?		
JUDGEMENT	RESEARCH EVIDENCE	ADDITIONAL CONSIDERATIONS
O Trivial<br>O Small	There are some indications that the risk for mortality is not significantly different after staged or delayed treatment for giant omphalocele. Three studies were included for this	EPSA summary

○ Moderate<br>○ Large<br>○ Varies<br>● Don't know	outcome. Bauman et al. 2016 systematically reviewed studies reporting on either staged surgical or delayed conservative management. 15/64 patients with staged reduction (23.4%) died versus 62/286 (21.8%) in the delayed group. The relative risk for mortality was 1.08 (95%CI 0.65-1.77) and was not significant (p=0.08). After this systematic review, two observational studies were published comparing staged and delayed reduction. Binet et al. 2020 reported a lower mortality rate for staged reduction, but the difference was not significant (RR 0.48, 95%CI 0.21-1.09, p=0.08). Menchaca et al. 2023 reported a lower mortality rate in the delayed group (0%) but also here, there was no significant difference with the staged group. (RR 6.0, 95%CI 0.26-136.53, p=0.26).<br><br>There are some indications that there is no significant difference in length of stay after staged or delayed treatment for giant omphalocele. Three studies were included for this outcome. Bauman et al. 2016 and Menchaca et al. 2023 both reported that there was no significant difference in length of stay between the staged and delayed patients (p=0.07 and p=0.221). However, Binet et al. reported a large difference in length of stay between staged and delayed closure (mean 95 days[range 49-174] vs. mean 11 days [range 1-60], p<0.01).<br><br>There are some indications that delayed closure (paint and wait) may result in shorter times to full enteral feeding compared to staged surgical closure. Two studies were included for this outcome. Bauman et al. 2016 reported a significant shorter time to full feeding for patients with giant omphalocele in the delayed group (mean 23.5 days vs. 14.6 days, p= 0.0018). Menchaca et al. 2023 reported shorter times to full feeding in the delayed group as well, but their difference wasn't significant (median 39 days vs. 28 days, p=0.6)	**Mortality:** The majority of panel members indicates some benefit; small (8), large (4). Others indicate 'don't know' as the cause of death is not reported.<br><br>**Ventilation:** The majority of panel members indicates no effect (16/19)<br><br>**Post-operative length of stay**: The majority of panel members indicates benefit; small (9), large (6). Others indicate 'don't know' as there are doubts if post-operative length of stay is a fair comparison. Due to missing data, the length of initial hospital stay could not be retrieved from the EPSA data.<br><br>**Time to full feeds:** The majority of the panel members indicate benefit; small (11), large (4). This would support findings in the literature (Bauman, Menchaca).<br><br>**Post-operative complications** ; The majority of the panel members indicate benefit; large (6), small (8)<br><br><u>Panel discussion</u><br>It was difficult for the panel to judge how substantial the desirable effects were. Literature as well as observations from the EPSA data indicate some benefits of non-operative management and delayed closure (mainly on the time to full feeds), however information on crucial outcomes such as neurodevelopmental outcome and length of initial hospital stay is missing. With that, it should be noted that panel members see the avoidance of repeated anaesthesia at neonatal age as one of the mere benefits of non-operative management and delayed closure. The observations from EPSA data indicate benefits in post-operative complications. While it seems logical on the one hand (no surgery equals no postoperative complications), panel members agree that it seems like there are less post-operative complications in a delayed closure procedure compared to surgeries as part of a staged reduction strategy. Another possible benefit of non-operative management is the avoidance of abdominal compartment syndrome. Panel members agree that based on the analysed data, literature and their personal experience that non-operative management and delayed closure is a proven and safe technique, that is suitable for

		high-risk patients. Panel members also agree that there are many unknown factors in predicting the success of staged closure, such as the role of the size of the abdomen.

Undesirable Effects

How substantial are the undesirable anticipated effects?

JUDGEMENT	RESEARCH EVIDENCE	ADDITIONAL CONSIDERATIONS
○ Trivial<br>○ Small<br>○ Moderate<br>○ Large<br>● Varies<br>○ Don't know		Panel discussion<br><br>While no significant harms of one intervention over the other are seen in the literature, panel members agree that the undesirable effects likely vary between patients and interventions. As there are many different techniques for staged closure, benefits and harms may vary between those techniques. Thereby, the expected benefits/harms ratio between non-surgical management and delayed closure and staged closure may also depend on the surgical skill to close the abdominal wall later on. Some surgeons may be very experienced with techniques like component separation, while others are not. On a patient level, panel members agree that active reduction has probably more harms than benefits for patients with pulmonary hypertension or lung hypoplasia. In these patients, one may prefer not to put additional compression on the abdomen.

Certainty of evidence

What is the overall certainty of the evidence of effects?

JUDGEMENT	RESEARCH EVIDENCE	ADDITIONAL CONSIDERATIONS
● Very low<br>O Low<br>O Moderate<br>O High<br>O No included studies	Outcomes were not corrected for confounders in any of the studies. The study of Binet et al., additionally may carry bias by study site, as all patients in the staged group were treated in a high resource hospital in France and all patients in the delayed group were treated in a lower resource hospital in the Ivory coast, without a neonatal unit and a lack of technical means for resuscitation and anaesthesia. This may have biased the results for lengths of stay as the more 'complicated' cases are alive and longer in hospital in France, while they already died in the Ivory coast and therefore don't have longer hospital stays. The study of Bauman et al. reports possible selection bias influencing length of stay outcomes as there were more patients with severe comorbidities in the delayed group. Specifically for the outcome 'mortality', the study by Menchaca et al. has very wide confidence intervals and low number of incidents-this raises concern for imprecision.	The observations based on EPSA data point in the same direction as literature conclusions. Both may have many confounders, so in fact, the EPSA data contributes to the conclusion that for many factors, we don’t know. However, part of the panel indicates that this data is helpful to confirm the few things we do know.

Values

Is there important uncertainty about or variability in how much people value the main outcomes?

JUDGEMENT	RESEARCH EVIDENCE	ADDITIONAL CONSIDERATIONS
O Important uncertainty or variability<br>● Possibly important uncertainty or variability<br>O Probably no important uncertainty or		Panel discussion<br><br>The panel agreed that there are possibly differences in how much the outcomes are valued by parents and clinicians. Clinicians likely value what they think is better for the patient and this may be influenced by scientific literature and/or their own experience. Patient representatives indicated that for parents, probably the possibility of early feeding and length of stay are highly valued outcomes. Panel members indicated that from their experience, the responses and

variability<br>O No important uncertainty or variability		value that is put on certain outcomes also depends on how parents are counselled and prepared for what they can expect.
**Balance of effects**<br>Does the balance between desirable and undesirable effects favor the intervention or the comparison?		
JUDGEMENT	RESEARCH EVIDENCE	ADDITIONAL CONSIDERATIONS
O Favors the comparison<br>O Probably favors the comparison<br>● Does not favor either the intervention or the comparison<br>O Probably favors the intervention<br>O Favors the intervention<br>O Varies<br>O Don't know		Panel discussion<br><br>Panel members agreed that based on the current data and their experiences, there are no indications for definite favour of one intervention over the other. Treatment choice likely depends on patient-related factors (lung condition, abdominal disproportion) and surgeons' experience.

Resources required

JUDGEMENT	RESEARCH EVIDENCE	ADDITIONAL CONSIDERATIONS
O Large costs<br>O Moderate costs<br>O Negligible costs and savings<br>O Moderate savings<br>O Large savings<br>O Varies<br>● Don't know		Panel discussion<br><br>The panel discussed the difference between longer (expected) initial hospital stay for non-operative management and the costs of more surgeries in case of staged reduction. If comparable cases are treated conservatively, there may still be the need for prolonged ventilatory support and NICU-treatment due to lung hypoplasia. In the case of paint and wait, surgery is still required – only at a delayed timepoint. However, fewer surgeries may mean lower costs overall. With the current data, nor with panel member experiences, it is not clear if one produces more costs compared to the other.

Certainty of evidence of required resources

What is the certainty of the evidence of resource requirements (costs)?

JUDGEMENT	RESEARCH EVIDENCE	ADDITIONAL CONSIDERATIONS
O Very low<br>O Low<br>O Moderate<br>O High<br>● No included studies		

Cost effectiveness

Does the cost-effectiveness of the intervention favor the intervention or the comparison?

JUDGEMENT	RESEARCH EVIDENCE	ADDITIONAL CONSIDERATIONS
O Favors the comparison<br>O Probably favors the comparison<br>O Does not favor either the intervention or the comparison<br>O Probably favors the intervention<br>O Favors the intervention<br>O Varies<br>● No included studies		

Equity

What would be the impact on health equity?

JUDGEMENT	RESEARCH EVIDENCE	ADDITIONAL CONSIDERATIONS
O Reduced<br>O Probably reduced<br>O Probably no impact<br>O Probably increased<br>● Increased<br>O Varies<br>O Don't know		Panel discussion<br><br>The recommendation will keep the practice variation alive. However, panel members agree that the recommendation probably leads to increased equity because there are no indications one intervention is to be favoured over the other. By letting everyone choose what they are most experienced in, chances for equal care quality increase, even if the offered interventions are different.

Acceptability
Is the intervention acceptable to key stakeholders?

JUDGEMENT	RESEARCH EVIDENCE	ADDITIONAL CONSIDERATIONS
O No<br>O Probably no<br>O Probably yes<br>● Yes<br>O Varies<br>O Don't know		Panel discussion<br><br>Panel members do not expect barriers to acceptability as the recommendation to choose either intervention lies close to the current situation. However, the panel formulated subgroup considerations for patients with pulmonary hypoplasia that are more based on expert consensus than on data. There may be surgeons that want to deviate from the recommendations for this subgroup based on their personal experience. The lack of evidence to enforce the opinion of this panel may lead to difficulties in accepting this subgroup recommendation. Parents may also anticipate a level of psychological distress stemming from their child having an unclosed fascia.

Feasibility
Is the intervention feasible to implement?

JUDGEMENT	RESEARCH EVIDENCE	ADDITIONAL CONSIDERATIONS
O No<br>O Probably no<br>O Probably yes<br>● Yes<br>O Varies<br>O Don't know		Panel discussion<br><br>Panel members do not expect barriers to feasibility as the recommendation to choose either intervention lies close to the current situation.

SUMMARY OF JUDGEMENTS

	JUDGEMENT						
PROBLEM	No	Probably no	Probably yes	Yes		Varies	Don't know

	JUDGEMENT						
**DESIRABLE EFFECTS**	Trivial	Small	Moderate	Large		Varies	**Don't know**
**UNDESIRABLE EFFECTS**	Trivial	Small	Moderate	Large		**Varies**	Don't know
**CERTAINTY OF EVIDENCE**	**Very low**	Low	Moderate	High			No included studies
**VALUES**	Important uncertainty or variability	**Possibly important uncertainty or variability**	Probably no important uncertainty or variability	No important uncertainty or variability			
**BALANCE OF EFFECTS**	Favors the comparison	Probably favors the comparison	**Does not favor either the intervention or the comparison**	Probably favors the intervention	Favors the intervention	Varies	Don't know
**RESOURCES REQUIRED**	Large costs	Moderate costs	Negligible costs and savings	Moderate savings	Large savings	Varies	Don't know
**CERTAINTY OF EVIDENCE OF REQUIRED RESOURCES**	Very low	Low	Moderate	High			**No included studies**
**COST EFFECTIVENESS**	Favors the comparison	Probably favors the comparison	Does not favor either the intervention or the comparison	Probably favors the intervention	Favors the intervention	Varies	**No included studies**
**EQUITY**	Reduced	Probably reduced	Probably no impact	Probably increased	**Increased**	Varies	Don't know
**ACCEPTABILITY**	No	Probably no	Probably yes	**Yes**		Varies	Don't know
**FEASIBILITY**	No	Probably no	Probably yes	**Yes**		Varies	Don't know

TYPE OF RECOMMENDATION

Strong recommendation against the intervention	Conditional recommendation against the intervention	**Conditional recommendation for either the intervention or the comparison**	Conditional recommendation for the intervention	Strong recommendation for the intervention
○	○	●	○	○

CONCLUSIONS

Recommendation

The panel suggests either non-operative treatment with delayed closure (NOM) or staged reduction and surgical repair (SRSR) for patients with giant omphalocele.

The panel suggests NOM for patients with respiratory distress, pulmonary hypertension or lung hypoplasia.

Justification

Balance of effects

Panel members agree that based on the current data and their experiences, there are no indications for the definite favour of one intervention over the other. Treatment choice likely depends on patient-related factors (lung condition, abdominal disproportion) and surgeons' experience.

Subgroup considerations

For patients with respiratory distress, pulmonary hypertension or lung hypoplasia, the panel judged that the balance between benefits and harms probably favours non-operative management and delayed closure. We considered only giant omphalocele in this chapter, however in the case of small omphalocele with significant lung hypoplasia, patients could also benefit from conservative management as no risks related to abdominal compression are added.

Implementation considerations

To gain insight into current practices pertaining to the management of omphalocele in Europe, a baseline survey has been conducted amongst centers involved in the European Reference Network for rare Inherited Congenital Anomalies (ERNICA) and/or connected to the European Pediatric Surgical Audit (EPSA). Center representatives are asked what their center's first choice of treatment is for giant omphalocele in the case that primary closure is not possible (Paint and wait [delayed closure] / staged reduction and surgical closure]). An opportunity is provided for further explanation.

A qualitative study will also take place to further explore the factors foreseen to hinder/facilitate successful implementation of the recommendation to perform NOM for patients with respiratory distress, pulmonary hypertension or lung hypoplasia. For example, the personal experience of the surgeons' and/or the lack of evidence may play a role. This exploration will be done with a view to collaboratively selecting implementation strategies.

Monitoring and evaluation

We intend to employ the European Pediatric Surgical Audit (EPSA) as a continuous feedback mechanism to monitor and evaluate current practices and (recommendation-specific) implementation success. The EPSA is an international prospective clinical audit registry. (The need for) supplementary measures for validation purposes will also be explored.

Research priorities

Panel members are interested in knowing how specific techniques for staged closure compare to non-operative management and delayed closure instead of the comparison of staged closure as a group. Future comparison with prospectively collected data may provide useful insights to guide the choice of treatment.

REFERENCES SUMMARY

Bauman et al. 2016 (reference5)

Binet et al. 2020 (reference 38)

Menchaca et al. 2023(reference 39)

QUESTION 3.2

Should a specific substance vs. other substances be used for conservative management of giant omphalocele ?	
**POPULATION:**	Conservative management of giant omphalocele
**INTERVENTION:**	A specific substance
**COMPARISON:**	Other substances
**MAIN OUTCOMES:**	Time to full epithelisation; Toxicity; Complications

ASSESSMENT

Desirable Effects<br>How substantial are the desirable anticipated effects?		
JUDGEMENT	RESEARCH EVIDENCE	ADDITIONAL CONSIDERATIONS
O Trivial<br>O Small<br>O Moderate<br>O Large	One systematic review could be included. Goneidy and Saxena systematically reviewed results for different kinds of substances. Substances reported on were Honey (53 patients), 2% aquous eosin (271 patients), Gentian violet (47 patients), silver dressings/solution (136 patients), povidone iodine (98 patients), mercurochrome (91	<u>EPSA summary</u><br><br>Dry cover could be compared to wound dressing or other substance.

○ Varies<br>○ Don't know	patients), Saline (18 patients), dry dressing only (75 patients) and mixed agents (42 patients). There is no evidence to indicate superiority of one agent over another for the outcomes time to epithelization and complication. There are some indications that less/non-toxic agents like honey and saline have similar performance to well performing toxic agents while decreasing toxicity risk.<br><br>Adjusted Evidence-to-Decision (ETD) questions were asked in the panel voice round before discussion. As this was a multi-intervention question, panel members were asked if, in their opinion, one or more agents had significantly more benefits compared to the others. Based on the presented evidence, most panel members think non-toxic agents like honey (11 votes) and saline (9 votes) have more benefits than other agents. A part of the panel indicated they could not decide based on the presented evidence.	**Days to surgery**: The majority of the panel members indicated harm from dry cover (7 small, 6 moderate) compared to wound dressing. Others indicated don't know (5) or no effect (2).<br>**Time to full feeds**: A large part of the panel indicated no effect (8), others indicated small harm (5) or don't know (6)<br><br>**Length of post-operative stay**: The majority of the panel members indicated no effect (10), others chose small benefit (3) or don't know (6). Many said that it was not perceived as a relevant outcome as it is postponed surgery.<br><br>Panel discussion.<br><br>It was difficult for the panel to make a judgement on beneficial effects as no substances were directly compared in the available literature, the evidence quality was poor and the EPSA data was very scattered with lots of missing values. Some panel members observed that there were more ventilated babies in the group wound dressing, with lower birth weight and more lung hypoplasia, this could have affected outcome. However, outcomes were almost similar compared to the group dry cover. This could be an indication that a wound dressing may act superior to a dry cover. Other panel members agreed with this hypothesis but were not convinced enough due to the small samples and poor data entry. Therefore, the panel decided no conclusions could be drawn on the magnitude of expected beneficial effects of one agent over another.

Undesirable Effects

How substantial are the undesirable anticipated effects?

JUDGEMENT	RESEARCH EVIDENCE	ADDITIONAL CONSIDERATIONS
O Trivial<br>O Small<br>O Moderate<br>O Large<br>O Varies<br>O Don't know	Adjusted ETD questions were asked in the panel voice round before discussion. As this was a multi-intervention question, panel members were asked if, in their opinion, one or more agents had significantly more undesirable effects compared to the others. Based on the presented evidence, most panel members think possibly toxic agents have more undesirable effects. The most indicated agents for having more undesirable effects than others were povidone iodine (17 votes), followed by mercurochrome (9 votes), and silver solutions (5 votes).	Panel discussion:<br><br>Overall, panel members agreed that mercurochrome, silver solutions and povidone iodine probably have more undesirable effects due to their toxicity compared to other agents.

Certainty of evidence

What is the overall certainty of the evidence of effects?

JUDGEMENT	RESEARCH EVIDENCE	ADDITIONAL CONSIDERATIONS
● Very low<br>O Low<br>O Moderate<br>O High<br>O No included studies	The presented evidence is based on a recent systematic review. Many studies included in the review are of poor quality. There are multiple confounding factors that are not accounted for, outcomes were poorly reported, and application and use of the agents was heterogenic between studies. There are many discrepancies between studies, one agent can be on the better side of performance in one study and on the worse end in another. The supplementary evidence based on observations from EPSA data also was considered of poor quality due to small sample sizes of the compared groups and many missing values.	

Values<br>Is there important uncertainty about or variability in how much people value the main outcomes?		
JUDGEMENT	RESEARCH EVIDENCE	ADDITIONAL CONSIDERATIONS
O Important uncertainty or variability<br>O Possibly important uncertainty or variability<br>● Probably no important uncertainty or variability<br>O No important uncertainty or variability		<u>Panel discussion:</u><br>Panel members cannot point out important differences in how the outcomes are valued. For parents as well as for clinicians, fast epithelization is prioritized. Panel members discussed that concerning efficiency, 'final epithelization' is difficult to measure.

Balance of effects<br>Does the balance between desirable and undesirable effects favor the intervention or the comparison?		
JUDGEMENT	RESEARCH EVIDENCE	ADDITIONAL CONSIDERATIONS
O Favors the comparison<br>O Probably favors the comparison<br>O Does not favor either the intervention or the comparison<br>O Probably favors the intervention		<u>Panel discussion:</u><br><br>Even with the low level of evidence, overall panel members agree that mercurochrome, silver solutions and povidone iodine probably have more undesirable effects compared to others. As there are no indications these substances with possible toxic side effects perform better compared to non-toxic substances like honey and saline, the panel agrees that they should be avoided. Larger prospective studies are desirable to confirm this.

O Favors the intervention<br>O Varies<br>O Don't know		

Resources required		
JUDGEMENT	RESEARCH EVIDENCE	ADDITIONAL CONSIDERATIONS
O Large costs<br>O Moderate costs<br>O Negligible costs and savings<br>● Moderate savings<br>O Large savings<br>O Varies<br>O Don't know		Panel discussion<br>Saline and honey are both cheaper than silver, this would further support a preference for these non-toxic agents.

Certainty of evidence of required resources

What is the certainty of the evidence of resource requirements (costs)?

JUDGEMENT	RESEARCH EVIDENCE	ADDITIONAL CONSIDERATIONS
O Very low<br>O Low<br>O Moderate<br>O High<br>● No included studies		

Cost effectiveness

Does the cost-effectiveness of the intervention favor the intervention or the comparison?

JUDGEMENT	RESEARCH EVIDENCE	ADDITIONAL CONSIDERATIONS
O Favors the comparison<br>O Probably favors the comparison<br>O Does not favor either the intervention or the comparison<br>O Probably favors the intervention<br>O Favors the intervention		

○ Varies<br>● No included studies		

Equity
What would be the impact on health equity?

JUDGEMENT	RESEARCH EVIDENCE	ADDITIONAL CONSIDERATIONS
○ Reduced<br>○ Probably reduced<br>○ Probably no impact<br>● Probably increased<br>○ Increased<br>○ Varies<br>○ Don't know		<u>Panel discussion</u><br>Panel members agreed that most of the non-toxic substances like honey and saline are easily accessible. Therefore, equity could be increased.

Acceptability
Is the intervention acceptable to key stakeholders?

JUDGEMENT	RESEARCH EVIDENCE	ADDITIONAL CONSIDERATIONS
○ No<br>○ Probably no<br>● Probably yes<br>○ Yes<br>○ Varies		<u>Panel discussion</u><br>Panel members agreed that a recommendation against possibly toxic substances is acceptable. As there are many alternatives, no one would prefer to purposely expose their patient/child to toxic substances. However, clinicians may be used to specific

O Don't know		substances and may not know how to use alternatives (e.g. honey). The poor evidence quality underpinning the benefits and harms of different substances, could be a barrier to acceptance around Europe.
**Feasibility**<br>Is the intervention feasible to implement?		
JUDGEMENT	RESEARCH EVIDENCE	ADDITIONAL CONSIDERATIONS
O No<br>O Probably no<br>● Probably yes<br>O Yes<br>O Varies<br>O Don't know		Panel discussion<br><br>Panel members agreed that this recommendation is feasible.

SUMMARY OF JUDGEMENTS

	JUDGEMENT						
**PROBLEM**	No	Probably no	Probably yes	Yes		Varies	Don't know
**DESIRABLE EFFECTS**	Trivial	Small	Moderate	Large		Varies	Don't know
**UNDESIRABLE EFFECTS**	Trivial	Small	Moderate	Large		Varies	Don't know
**CERTAINTY OF EVIDENCE**	**Very low**	Low	Moderate	High			No included studies
**VALUES**	Important uncertainty or variability	Possibly important uncertainty or variability	**Probably no important uncertainty or variability**	No important uncertainty or variability			
**BALANCE OF EFFECTS**	Favors the comparison	Probably favors the comparison	Does not favor either the	Probably favors the intervention	Favors the intervention	Varies	Don't know

	JUDGEMENT						
			intervention or the comparison				
**RESOURCES REQUIRED**	Large costs	Moderate costs	Negligible costs and savings	Moderate savings	Large savings	Varies	**Don't know**
**CERTAINTY OF EVIDENCE OF REQUIRED RESOURCES**	Very low	Low	Moderate	High			**No included studies**
**COST EFFECTIVENESS**	Favors the comparison	Probably favors the comparison	Does not favor either the intervention or the comparison	Probably favors the intervention	Favors the intervention	Varies	**No included studies**
**EQUITY**	Reduced	Probably reduced	Probably no impact	**Probably increased**	Increased	Varies	Don't know
**ACCEPTABILITY**	No	Probably no	**Probably yes**	Yes		Varies	Don't know
**FEASIBILITY**	No	Probably no	**Probably yes**	Yes		Varies	Don't know

TYPE OF RECOMMENDATION

Strong recommendation against the intervention	Conditional recommendation against the intervention	Conditional recommendation for either the intervention or the comparison	Conditional recommendation for the intervention	Strong recommendation for the intervention
●	○	○	○	○

CONCLUSIONS

Recommendation

The panel recommends refraining from using mercurochrome, silver solutions and povidone iodine for the non-operative management of giant omphalocele due to their possible toxic side effects. The panel suggests the use of non-toxic substances (with less side effects) such as honey or saline.

Justification

Undesirable effects
Overall, panel members agree that mercurochrome, silver solutions and povidone iodine probably have more undesirable effects compared to others.

Balance of effects
Even with the low level of evidence, overall panel members agree that mercurochrome, silver solutions and povidone iodine probably have more undesirable effects compared to others. As there are no indications these substances with possible toxic side effects perform better compared to non-toxic substances like honey and saline, the panel agrees that they should be avoided.

Subgroup considerations

Implementation considerations

To gain insight into current practices pertaining to the management of omphalocele in Europe, a baseline survey has been conducted amongst centers involved in the European Reference Network for rare Inherited Congenital Anomalies (ERNICA) and/or connected to the European Pediatric Surgical Audit (EPSA). Center representatives are asked what is/are their centre's preferred topical substance(s) to use for the management of giant omphalocele in the case of paint and wait. They are given the option to provide an explanation for their answer.

A qualitative study will also take place to further explore the factors foreseen to hinder/facilitate successful implementation of this recommendation in clinical practice. For example, it may be that clinicians do not have experience/know how to use alternative substances like honey. This exploration will be done with a view to collaboratively selecting implementation strategies. As an expertise network, ERNICA is well placed to facilitate the organisation of educational initiatives to support gaps in knowledge.

Monitoring and evaluation

We intend to employ the European Pediatric Surgical Audit (EPSA) as a continuous feedback mechanism to monitor and evaluate (recommendation-specific) implementation success. The EPSA is an international prospective clinical audit registry. (The need for) supplementary measures for validation purposes will also be explored.

Research priorities

Prospective studies comparing different agents should be conducted to get better insights into the difference in benefits between substances.

REFERENCES SUMMARY

Goneidy and Saxena, 2023 (reference 40)

QUESTION 3.3

Should a specific intervention vs. other interventions be used for staged closure of giant omphalocele ?	
**POPULATION:**	Staged closure of giant omphalocele
**INTERVENTION:**	A specific intervention
**COMPARISON:**	Other interventions
**MAIN OUTCOMES:**	Time on ventilator, Feeding outcomes, Time to full closure (days), Length of stay (days), Occurrence of infection or sepsis, Possibility for nursing

ASSESSMENT

Desirable Effects<br>How substantial are the desirable anticipated effects?		
JUDGEMENT	RESEARCH EVIDENCE	ADDITIONAL CONSIDERATIONS
○ Trivial<br>○ Small<br>○ Moderate<br>○ Large<br>○ Varies<br>○ Don't know	Research evidence was too heterogenous to pool any of the outcomes. Panel members were presented with a matrix of staged closure methods and outcomes that was filled in as much as possible according to published literature. Included interventions were Surgical silo, Non-surgical silo (Duoderm®), Barlow external silo, Patch sutured over the omphalocele, Fasciotens®, Traction, Taping and Suspension.<br><br>Based on this matrix, panel members answered adjusted evidence to decision questions via a digital survey.<br><br>Most panel members (12/26) indicated that based on the presented evidence, they could not judge if one intervention had more desirable effects than others. Other panel members indicated they think there are more benefits with surgical silo	Panel discussion<br><br>The panel members could not identify any clear benefits of one method over the other. To make better judgements, the terminology of 'staged reduction' needs to be defined better as for some included interventions, panel members are not in agreement whether they would call it a 'staged' procedure.

	(5), Non-surgical silo (5), GoreTex® patch sutured over the omphalocele (4) or Fasciotens® (1).	

Undesirable Effects

How substantial are the undesirable anticipated effects?

JUDGEMENT	RESEARCH EVIDENCE	ADDITIONAL CONSIDERATIONS
O Trivial<br>O Small<br>O Moderate<br>O Large<br>O Varies<br>O Don't know	The majority of panel members (14/26) answered that based on the presented data, they could not indicate if one technique had more undesirable effects over another, or didn't know which one.<br><br>Other panel members mentioned the following interventions with possibly undesirable effects:<br><br>- Non surgical silo with DuoDerm (4) for its seemingly high rate of sepsis<br><br>- Patch sutured over the omphalocele (3), as it seems to have no advantages over delayed closure so conservative management could replace the surgery for patch placement. Previous patch placement could increase surgical difficulties with the definitive closure of the abdominal wall.	<u>Panel discussion</u><br>The panel cannot agree on any clear undesirable effects of some interventions compared to others. While a patch sutured over the omphalocele was mentioned in the survey, other panel members explain that it really depends on how is it used. For example, when there are sutures for tension placed on the patch, it does really differ from a conservative treatment.

Certainty of evidence

What is the overall certainty of the evidence of effects?

JUDGEMENT	RESEARCH EVIDENCE	ADDITIONAL CONSIDERATIONS
● Very low<br>O Low<br>O Moderate<br>O High<br>O No included studies	All studies included in the matrix were case series at best. Overall, there was a lack of accurate correction for confounders.	

Values

Is there important uncertainty about or variability in how much people value the main outcomes?

JUDGEMENT	RESEARCH EVIDENCE	ADDITIONAL CONSIDERATIONS
O Important uncertainty or variability<br>● Possibly important uncertainty or variability<br>O Probably no important uncertainty or variability<br>O No important uncertainty or variability		Panel discussion<br><br>The panel agreed that the choice of an intervention is not so much based on outcomes, but more on experience. The staged procedure is a rare occasion for most involved surgeons so if they are experienced with / trained on one intervention, they may counsel parents based on previous experiences with this intervention. The panel members agreed that counselling in a multidisciplinary team is very important in these cases. Panel members noted that in their experience, parents value a thorough explanation of the treatment process.

Balance of effects

Does the balance between desirable and undesirable effects favor the intervention or the comparison?

JUDGEMENT	RESEARCH EVIDENCE	ADDITIONAL CONSIDERATIONS
O Favors the comparison<br>O Probably favors the comparison<br>O Does not favor either the intervention or the comparison<br>O Probably favors the intervention<br>O Favors the intervention<br>O Varies<br>● Don't know		Panel discussion<br>Panel members concluded that they have too little information to indicate the balance of effects in favor of one or more specific interventions.

Resources required

JUDGEMENT	RESEARCH EVIDENCE	ADDITIONAL CONSIDERATIONS
O Large costs<br>O Moderate costs<br>O Negligible costs and savings<br>O Moderate savings<br>O Large savings<br>● Varies<br>O Don't know		Panel discussion<br>Some interventions seem to carry higher costs per patient compared to others. Compared to other materials like Shuster plastic, DuoDerm® is more expensive and might not be available everywhere. Panel members also mentioned that the use of Fasciotens® and sutured patches may have cost and resource implications.

Certainty of evidence of required resources<br>What is the certainty of the evidence of resource requirements (costs)?		
JUDGEMENT	RESEARCH EVIDENCE	ADDITIONAL CONSIDERATIONS
O Very low<br>O Low<br>O Moderate<br>O High<br>● No included studies		

Cost effectiveness<br>Does the cost-effectiveness of the intervention favor the intervention or the comparison?		
JUDGEMENT	RESEARCH EVIDENCE	ADDITIONAL CONSIDERATIONS
O Favors the comparison<br>O Probably favors the comparison<br>O Does not favor either the intervention or the comparison<br>O Probably favors the intervention<br>O Favors the intervention<br>O Varies<br>● No included		

studies		

Equity
What would be the impact on health equity?

JUDGEMENT	RESEARCH EVIDENCE	ADDITIONAL CONSIDERATIONS
O Reduced<br>O Probably reduced<br>O Probably no impact<br>O Probably increased<br>O Increased<br>● Varies<br>O Don't know		Panel discussion<br>Equity may be decreased as practice variation is high. However, panel members agree that all of the studied interventions are complex and require specific expertise. Specific expertise in one intervention, and consistently practicing this intervention makes it more likely that a surgical team can provide good quality care, regardless of the method. A recommendation for either of the interventions may therefore increase equity.

Acceptability
Is the intervention acceptable to key stakeholders?

JUDGEMENT	RESEARCH EVIDENCE	ADDITIONAL CONSIDERATIONS
O No<br>O Probably no<br>O Probably yes<br>● Yes<br>O Varies		Panel discussion<br>The panel foresees no important barriers to the acceptability of a conditional recommendation for either of the interventions. No specific technique is recommended.

O Don't know		

Feasibility
Is the intervention feasible to implement?

JUDGEMENT	RESEARCH EVIDENCE	ADDITIONAL CONSIDERATIONS
O No<br>O Probably no<br>O Probably yes<br>● Yes<br>O Varies<br>O Don't know		Panel discussion<br>A recommendation for one or more specific interventions would prompt the need for training in some centers, as well as the purchasing of specific machines or materials. The panel foresees no important barriers to the feasibility of a conditional recommendation for either of the interventions and this is likely to be more feasible than a specific recommendation.

SUMMARY OF JUDGEMENTS

	JUDGEMENT						
PROBLEM	No	Probably no	Probably yes	Yes		Varies	Don't know
DESIRABLE EFFECTS	Trivial	Small	Moderate	Large		Varies	Don't know
UNDESIRABLE EFFECTS	Trivial	Small	Moderate	Large		Varies	Don't know
CERTAINTY OF EVIDENCE	**Very low**	Low	Moderate	High			No included studies
VALUES	Important uncertainty or variability	**Possibly important uncertainty or variability**	Probably no important uncertainty or variability	No important uncertainty or variability			
BALANCE OF EFFECTS	Favors the comparison	Probably favors the comparison	**Does not favor either the**	Probably favors the intervention	Favors the intervention	Varies	Don't know

	JUDGEMENT						
			**intervention or the comparison**				
**RESOURCES REQUIRED**	Large costs	Moderate costs	Negligible costs and savings	Moderate savings	Large savings	**Varies**	Don't know
**CERTAINTY OF EVIDENCE OF REQUIRED RESOURCES**	Very low	Low	Moderate	High			**No included studies**
**COST EFFECTIVENESS**	Favors the comparison	Probably favors the comparison	Does not favor either the intervention or the comparison	Probably favors the intervention	Favors the intervention	Varies	**No included studies**
**EQUITY**	Reduced	Probably reduced	Probably no impact	**Probably increased**	Increased	Varies	Don't know
**ACCEPTABILITY**	No	Probably no	Probably yes	**Yes**		Varies	Don't know
**FEASIBILITY**	No	Probably no	Probably yes	**Yes**		Varies	Don't know

TYPE OF RECOMMENDATION

Strong recommendation against the intervention	Conditional recommendation against the intervention	Conditional recommendation for either the intervention or the comparison	Conditional recommendation for the intervention	Strong recommendation for the intervention
○	○	●	○	○

CONCLUSIONS

Recommendation

The panel suggests to choose interventions for staged closure based on the center’s experience.

Justification

Balance of effects
Panel members conclude that they have too little information to indicate the balance of effects in favor of one or more specific interventions.

Equity
Equity may be decreased as practice variation is high. However, panel members agree that all of the studied interventions are complex and require specific expertise. Specific expertise in one intervention, and consistently practicing this intervention makes it more likely that a surgeon can provide good quality care, regardless of the method. A recommendation for either of the interventions may therefore increase equity.

Subgroup considerations

Implementation considerations

To gain insight into current practices pertaining to the management of omphalocele in Europe, a baseline survey has been conducted amongst centers involved in the European Reference Network for rare Inherited Congenital Anomalies (ERNICA) and/or connected to the European Pediatric Surgical Audit (EPSA). Center representatives are asked which methods their center would consider (and have available) for staged reduction and closure of the abdominal wall in giant omphalocele. Explanations may be provided. As an expertise network, ERNICA can play a role in faciliating the exchange of knowledge/experience on current practices.

Monitoring and evaluation

We intend to employ the European Pediatric Surgical Audit (EPSA) as a continuous feedback mechanism to monitor and evaluate center practices. The EPSA is an international prospective clinical audit registry.

Research priorities

The panel members expressed the desire for better definitions of the different interventions. If this is established, prospective comparisons between two well-defined interventions can be set up. Until then, exchange of expertise within the network remains an important factor in the achievement of high quality care. Discussion of cases of giant omphalocele via the Central Patient Management System (CPMS 2.0) may aid in this. To further support the sharing of knowledge and expertise, experts desire to make video lectures about the different techniques they each use. The panel also noted that it would be of interest to explore what parents would like to be included in multidisciplinary counselling.

REFERENCES SUMMARY

Kogut et al. 2018 (reference 7)

Binet et al. 2020 (reference 38)

Abello et al. 2021 (reference 41)

Barrios-Sanjuanelo et al. 2021 (reference 42)

Huang et al. 2021 (reference 43)

Mitanchez et al. 2010 (reference 44)

Pacilli et al. 2005 (reference 45)

Saxena et al. 2002 (reference 46)

Uecker et al. 2020 (reference 47)

Ziegler et al. 2024 (reference 48)

QUESTION 3.4

Should earlier (0-1-2 days) vs. later timing of surgery be used for primary closure of small omphalocele?	
**POPULATION:**	Primary closure of small omphalocele
**INTERVENTION:**	Earlier (0-1-2 days)
**COMPARISON:**	Later timing of surgery
**MAIN OUTCOMES:**	Mortality, Operative complications, Length of hospital stay

ASSESSMENT

Problem<br>Is the problem a priority?		
JUDGEMENT	RESEARCH EVIDENCE	ADDITIONAL CONSIDERATIONS
O No<br>O Probably no<br>O Probably yes<br>O Yes<br>O Varies<br>O Don't know		
**Desirable Effects**<br>How substantial are the desirable anticipated effects?		
JUDGEMENT	RESEARCH EVIDENCE	ADDITIONAL CONSIDERATIONS
● Trivial<br>O Small<br>O Moderate<br>O Large	No published evidence could be included.	<u>EPSA summary:</u><br><br>**Mortality**: Panel members have indicated mostly no effect (7), small harm (5) or don't know (5) (difficult to judge as we don't know the cause of death, could have been comorbidities and not the timing of surgery)

O Varies<br>O Don't know		**Post-operative complications**: The majority of panel members have indicated benefit in the early group: large benefit (2) moderate benefit (1) small benefit (10). Other answers were no effect (4) or don't know (2)<br><br>**Length of post-operative hospital stay**: Most panel members indicated no effect (13)<br><br>**Time to full feeds**: Most panel members indicated no effect (9) to small benefit (7). Two panel members indicated small harm with consideration that this could become no effect if data was corrected for more premature and low birthweight babies in the early group.<br><br><u>Panel discussion:</u><br><br>Discussing the outcomes of the structured observation forms, panel members started to question the intention to treat in both groups. Decisions on the timing of surgery could be based on patient characteristics, but also on organizational factors such as the availability of beds or the right team. Bearing in mind that none of the panel members observed differences in mortality between groups or in post-operative hospital stay and most panel members did not see differences in post-operative complications between groups, based on this data there seems to be little reason to do an early surgery or to consider the surgery as an emergency procedure.

Undesirable Effects

How substantial are the undesirable anticipated effects?

JUDGEMENT	RESEARCH EVIDENCE	ADDITIONAL CONSIDERATIONS
●Trivial<br>O Small<br>O Moderate<br>O Large<br>O Varies<br>O Don't know		<u>Panel discussion:</u><br><br>From the EPSA data, it seems that a later operation could be associated with more ventilation days. Panel members expect this has more to do with the condition of the patient and their comorbidities than with the choice for timing of surgery.

Certainty of evidence<br>What is the overall certainty of the evidence of effects?		
JUDGEMENT	RESEARCH EVIDENCE	ADDITIONAL CONSIDERATIONS
O Very low<br>O Low<br>O Moderate<br>O High<br>●No included studies		There are no studies included. The certainty of conclusions based on the EPSA data is impaired by a lack of a registered intention to treat.

Values<br>Is there important uncertainty about or variability in how much people value the main outcomes?		
JUDGEMENT	RESEARCH EVIDENCE	ADDITIONAL CONSIDERATIONS
O Important uncertainty or variability<br>O Possibly important uncertainty or variability<br>O Probably no important uncertainty or variability<br>● No important uncertainty or variability		The panel expects no important variability in how much different stakeholders (parents and clinicians) value the main outcomes.

Balance of effects<br>Does the balance between desirable and undesirable effects favor the intervention or the comparison?		
JUDGEMENT	RESEARCH EVIDENCE	ADDITIONAL CONSIDERATIONS
O Favors the comparison<br>●Probably favors the comparison<br>O Does not favor either the intervention or the comparison<br>O Probably favors the intervention<br>O Favors the intervention<br>O Varies<br>O Don't know		Panel discussion<br><br>Bearing in mind that none of the panel members observed differences in mortality between groups or in post-operative hospital stay and most panel members did not see differences in post-operative complications between groups, based on this data there seems to be little reason to do an early surgery or to consider the surgery as an emergency procedure. Panel members agree that next to patient characteristics such as respiratory status or comorbidities that warrant attention, local organization of care has a role in the timing of surgery and that waiting for the best team to be available could increase the quality of care.
Resources required		
JUDGEMENT	RESEARCH EVIDENCE	ADDITIONAL CONSIDERATIONS
O Large costs<br>O Moderate costs<br>● Negligible costs and savings<br>O Moderate savings<br>O Large savings<br>O Varies<br>O Don't know		Panel discussion<br><br>Panel members don’t think timing of surgery will have a substantial impact on costs and resource needs.

Certainty of evidence of required resources

What is the certainty of the evidence of resource requirements (costs)?

JUDGEMENT	RESEARCH EVIDENCE	ADDITIONAL CONSIDERATIONS
O Very low<br>O Low<br>O Moderate<br>O High<br>● No included studies		

Cost effectiveness

Does the cost-effectiveness of the intervention favor the intervention or the comparison?

JUDGEMENT	RESEARCH EVIDENCE	ADDITIONAL CONSIDERATIONS
O Favors the comparison<br>O Probably favors the comparison<br>O Does not favor either the intervention or the comparison<br>O Probably favors the intervention<br>O Favors the intervention<br>O Varies<br>● No included studies		

Equity<br>What would be the impact on health equity?		
JUDGEMENT	RESEARCH EVIDENCE	ADDITIONAL CONSIDERATIONS
○ Reduced<br>○ Probably reduced<br>○ Probably no impact<br>○ Probably increased<br>● Increased<br>○ Varies<br>○ Don't know		Panel discussion<br><br>The panel discussed that there are always additional risks to emergency procedures. Not considering primary closure of a non-giant omphalocele as an emergency procedure could increase care equity, as planned surgery with the right team decreases the risks that are attached to emergency procedures.

Acceptability<br>Is the intervention acceptable to key stakeholders?		
JUDGEMENT	RESEARCH EVIDENCE	ADDITIONAL CONSIDERATIONS
○ No<br>○ Probably no<br>○ Probably yes<br>● Yes<br>○ Varies<br>○ Don't know		Panel discussion<br><br>Panel members expect that many hospitals are not handling this as an emergency at present.<br><br>Additional panel views:<br><br>Waiting longer for the surgery to take place may cause some distress for the parents.

Feasibility<br>Is the intervention feasible to implement?		
JUDGEMENT	RESEARCH EVIDENCE	ADDITIONAL CONSIDERATIONS
○ No<br>○ Probably no<br>○ Probably yes<br>● Yes<br>○ Varies		Panel discussion<br><br>Panel members expect all surgical timings to be feasible.

○ Don't know		

SUMMARY OF JUDGEMENTS

	JUDGEMENT						
**PROBLEM**	No	Probably no	Probably yes	Yes		Varies	Don't know
**DESIRABLE EFFECTS**	**Trivial**	Small	Moderate	Large		Varies	Don't know
**UNDESIRABLE EFFECTS**	**Trivial**	Small	Moderate	Large		Varies	Don't know
**CERTAINTY OF EVIDENCE**	Very low	Low	Moderate	High			**No included studies**
**VALUES**	Important uncertainty or variability	Possibly important uncertainty or variability	Probably no important uncertainty or variability	**No important uncertainty or variability**			
**BALANCE OF EFFECTS**	Favors the comparison	**Probably favors the comparison**	Does not favor either the intervention or the comparison	Probably favors the intervention	Favors the intervention	Varies	Don't know
**RESOURCES REQUIRED**	Large costs	Moderate costs	**Negligible costs and savings**	Moderate savings	Large savings	Varies	Don't know
**CERTAINTY OF EVIDENCE OF REQUIRED RESOURCES**	Very low	Low	Moderate	High			**No included studies**
**COST EFFECTIVENESS**	Favors the comparison	Probably favors the comparison	Does not favor either the intervention or the comparison	Probably favors the intervention	Favors the intervention	Varies	**No included studies**

	JUDGEMENT						
EQUITY	Reduced	Probably reduced	Probably no impact	Probably increased	**Increased**	Varies	Don't know
ACCEPTABILITY	No	Probably no	Probably yes	**Yes**		Varies	Don't know
FEASIBILITY	No	Probably no	Probably yes	**Yes**		Varies	Don't know

TYPE OF RECOMMENDATION

Strong recommendation against the intervention	Conditional recommendation against the intervention	Conditional recommendation for either the intervention or the comparison	Conditional recommendation for the intervention	Strong recommendation for the intervention
●	○	○	○	○

CONCLUSIONS

Recommendation

The panel recommends to choose the timing of surgery (in non-giant, non-ruptured omphalocele) based on the condition of the patient and the availability of the right team; the procedure should not be considered an emergency.

Justification

Balance of effects
Bearing in mind that none of the panel members observed differences in mortality between groups or in post-operative hospital stay and most panel members did not see differences in post-operative complications between groups, based on this data there seems to be little reason to do an early surgery or to consider the surgery as an emergency procedure. Panel members agree that next to patient characteristics such as respiratory status or comorbidities that warrant attention, local organization of care has a role in the timing of surgery and that waiting for the best team to be available could increase the quality of care.

Subgroup considerations

-

Implementation considerations

We intend to explore current practices pertaining to the timing of surgery for small omphalocele (amongst centers part of ERNICA/connected to the EPSA). These insights can be used as a basis for ongoing implementation efforts.

Monitoring and evaluation

We intend to employ the European Pediatric Surgical Audit (EPSA) as a continuous feedback mechanism to monitor and evaluate center practices. The EPSA is an international prospective clinical audit registry. (The need for) supplementary measures for validation purposes will also be explored.

Research priorities

The panel did not identify a particular research priority for this topic.

QUESTION 3.5

Should surgery before 1 year vs. after 1 year be used for delayed closure after conservative management of giant omphalocele?	
**POPULATION:**	Delayed closure after conservative management of giant omphalocele
**INTERVENTION:**	Surgery before 1 year
**COMPARISON:**	After 1 year
**MAIN OUTCOMES:**	Length of stay, Complications

ASSESSMENT

Desirable Effects<br>How substantial are the desirable anticipated effects?		
JUDGEMENT	RESEARCH EVIDENCE	ADDITIONAL CONSIDERATIONS
○ Trivial<br>● Small<br>○ Moderate<br>○ Large<br>○ Varies<br>○ Don't know	No evidence could be included for this question	EPSA summary:<br><br>**Length of post-operative hospital stay:** About half of the panel members indicated that an earlier operation may be associated with harm in terms of post-operative hospital stay (small harm: 5, moderate harm: 5). The other half of the panel either indicated no effect (5) or don't know (5).<br><br>**Post-operative complications:**<br><br>Panel members indicated mostly no effect (8) or don't know (6). Panel members who voted for 'don't know' mainly explained that this was because of the small numbers and/or missing data. Four panel members indicated more complications with earlier surgery (small harm).<br><br>Panel discussion:<br><br>For this data, an intention to treat was also not available. Panel members discussed that comorbidity may have impacted outcome as well as choice for timing of surgery. Patients in the later group (>1 year of age) seem to have more cardiac anomalies and a higher rate of prematurity, but no important differences in length of postoperative hospital stay or complications are seen-some panel members even observed less

		complications and shorter length of stay in this group. The condition of the patients may have been a confounder for these outcomes. Panel members discussed that this may plea in favor of surgery after 1 year of age.

Undesirable Effects

How substantial are the undesirable anticipated effects?

JUDGEMENT	RESEARCH EVIDENCE	ADDITIONAL CONSIDERATIONS
O Trivial<br>O Small<br>● Moderate<br>O Large<br>O Varies<br>O Don't know		Panel discussion<br>Neonatologists on the panel emphasize that elective surgery and general anesthesia in the first year of life may have negative effects on neurological development and that this outcome is not taken into account in the evaluated data. While we don't have evidence for this in the omphalocele population, evidence is present for pediatric patients with other surgical conditions such as congenital diaphragmatic hernia and esophageal atresia.[1-6]

Certainty of evidence

What is the overall certainty of the evidence of effects?

JUDGEMENT	RESEARCH EVIDENCE	ADDITIONAL CONSIDERATIONS
O Very low<br>O Low<br>O Moderate<br>O High<br>●No included studies		There are no studies included. The certainty of conclusions based on the EPSA data is impaired by a lack of a registered intention to treat. Panel members agreed that the difference between groups (more cardiac defects and prematurity in the late group) could be a confounding factor and increases trust in the conclusion that delayed closure surgery after 1 year may be favorable.

Values<br>Is there important uncertainty about or variability in how much people value the main outcomes?		
JUDGEMENT	RESEARCH EVIDENCE	ADDITIONAL CONSIDERATIONS
O Important uncertainty or variability<br>● Possibly important uncertainty or variability<br>O Probably no important uncertainty or variability<br>ONo important uncertainty or variability		<u>Panel discussion</u><br><br>The panel detected some important variability in how different stakeholders (parents and clinicians) value the main outcomes. For some parents, it may be important to close the abdominal wall of their child as soon as possible, as a closed abdominal wall and reconstruction of the umbilicus could be associated with normalcy by parents (e.g. allowing them to crawl etc). For clinicians, there may not be so much urgency to close the abdominal wall. However, this will depend on the patient's condition.

Balance of effects<br>Does the balance between desirable and undesirable effects favor the intervention or the comparison?		
JUDGEMENT	RESEARCH EVIDENCE	ADDITIONAL CONSIDERATIONS
O Favors the comparison<br>● Probably favors the comparison<br>O Does not favor either the intervention or the comparison<br>O Probably favors the intervention<br>O Favors the intervention<br>O Varies<br>O Don't know		<u>Panel discussion</u><br><br>Based on the evaluated EPSA data and expert experiences, there seem to be little benefits of performing the delayed closure before 1 year of age and minimal to no harm to postponing to after 1 year of age, while surgery after one year may be associated with shorter length of postoperative hospital stay. Some parents may favor earlier surgery, as closure of the abdominal wall and reconstruction of the umbilicus is associated with the feeling of having a normal child. The personal preference of parents should be considered in the decision about surgical timing.

Resources required

JUDGEMENT	RESEARCH EVIDENCE	ADDITIONAL CONSIDERATIONS
O Large costs<br>O Moderate costs<br>● Negligible costs and savings<br>O Moderate savings<br>O Large savings<br>O Varies<br>O Don't know		Panel discussion<br><br>If the length of stay is indeed shorter when patients are operated on after one year of age, this could possibly decrease the costs per patient. Overall, giant omphaloceles that require delayed repair after initial non-operative management are still quite rare, meaning their impact on overall healthcare costs will be minimal.

Certainty of evidence of required resources

What is the certainty of the evidence of resource requirements (costs)?

JUDGEMENT	RESEARCH EVIDENCE	ADDITIONAL CONSIDERATIONS
O Very low<br>O Low<br>O Moderate<br>O High<br>● No included studies		

Cost effectiveness

Does the cost-effectiveness of the intervention favor the intervention or the comparison?

JUDGEMENT	RESEARCH EVIDENCE	ADDITIONAL CONSIDERATIONS
○ Favors the comparison<br>○ Probably favors the comparison<br>○ Does not favor either the intervention or the comparison<br>○ Probably favors the intervention<br>○ Favors the intervention<br>○ Varies<br>● No included studies		

Equity

What would be the impact on health equity?

JUDGEMENT	RESEARCH EVIDENCE	ADDITIONAL CONSIDERATIONS
○ Reduced<br>○ Probably reduced<br>● Probably no impact<br>○ Probably increased<br>○ Increased<br>○ Varies<br>○ Don't know		Panel discussion<br><br>Panel members expect little to no impact of timing of surgery on health equity.

Acceptability<br>Is the intervention acceptable to key stakeholders?		
JUDGEMENT	RESEARCH EVIDENCE	ADDITIONAL CONSIDERATIONS
O No<br>O Probably no<br>● Probably yes<br>O Yes<br>O Varies<br>O Don't know		Panel discussion<br><br>Panel members agreed that a recommendation against the intervention (surgery before 1 year of age) is probably acceptable. However, some parents may wish the surgeon to perform the closure earlier as they associate a closed abdomen with a normal child.

Feasibility<br>Is the intervention feasible to implement?		
JUDGEMENT	RESEARCH EVIDENCE	ADDITIONAL CONSIDERATIONS
O No<br>O Probably no<br>O Probably yes<br>● Yes<br>O Varies<br>O Don't know		Panel discussion<br><br>Panel members expect all surgical timings to be feasible.

SUMMARY OF JUDGEMENTS

	JUDGEMENT						
**PROBLEM**	No	Probably no	Probably yes	Yes		Varies	Don't know
**DESIRABLE EFFECTS**	Trivial	**Small**	Moderate	Large		Varies	Don't know
**UNDESIRABLE EFFECTS**	Trivial	**Small**	Moderate	Large		Varies	Don't know
**CERTAINTY OF EVIDENCE**	Very low	Low	Moderate	High			**No included studies**

	JUDGEMENT						
VALUES	Important uncertainty or variability	**Possibly important uncertainty or variability**	Probably no important uncertainty or variability	No important uncertainty or variability			
BALANCE OF EFFECTS	Favors the comparison	**Probably favors the comparison**	Does not favor either the intervention or the comparison	Probably favors the intervention	Favors the intervention	Varies	Don't know
RESOURCES REQUIRED	Large costs	Moderate costs	**Negligible costs and savings**	Moderate savings	Large savings	Varies	Don't know
CERTAINTY OF EVIDENCE OF REQUIRED RESOURCES	Very low	Low	Moderate	High			**No included studies**
COST EFFECTIVENESS	Favors the comparison	Probably favors the comparison	Does not favor either the intervention or the comparison	Probably favors the intervention	Favors the intervention	Varies	**No included studies**
EQUITY	Reduced	Probably reduced	**Probably no impact**	Probably increased	Increased	Varies	Don't know
ACCEPTABILITY	No	Probably no	**Probably yes**	Yes		Varies	Don't know
FEASIBILITY	No	Probably no	Probably yes	**Yes**		Varies	Don't know

TYPE OF RECOMMENDATION

Strong recommendation against the intervention	**Conditional recommendation against the intervention**	Conditional recommendation for either the intervention or the comparison	Conditional recommendation for the intervention	Strong recommendation for the intervention
○	●	○	○	○

CONCLUSIONS

Recommendation

For patients with giant omphalocele, the panel suggests to plan delayed closure of the abdominal wall after initial non-operative treatment not before the patient reaches the (corrected) age of 1 year old.

Justification

Balance of effects
Based on the evaluated EPSA data and expert experiences, there seem to be little benefits of performing the delayed closure before 1 year of age and minimal to no harms to postponing to after 1 year of age, while surgery after one year may be associated with shorter length of post-operative hospital stay. However, some parents may favor earlier surgery, in which case this should be considered. Therefore, the panel decided on a conditional recommendation.

Subgroup considerations

Implementation considerations

To gain insight into current practices pertaining to the management of omphalocele in Europe, a baseline survey has been conducted amongst centers involved in the European Reference Network for rare Inherited Congenital Anomalies (ERNICA) and/or connected to the European Pediatric Surgical Audit (EPSA). Center representatives are asked at what time point their center performs surgical closure of the abdominal wall in patients with giant omphalocele after paint and wait (Before 1 year of age/after 1 year of age/it depends). Respondents are given the opportunity to provide explanations.

A qualitative study will also take place to further explore the factors foreseen to hinder/facilitate successful implementation of this recommendation in clinical practice. This exploration will be done with a view to collaboratively selecting implementation strategies.

Monitoring and evaluation

We intend to employ the European Pediatric Surgical Audit (EPSA) as a continuous feedback mechanism to monitor and evaluate (recommendation-specific) implementation success. The EPSA is an international prospective clinical audit registry. (The need for) supplementary measures for validation purposes will also be explored.

Research priorities

Panel members identified several research priorities pertaining to this topic. As we currently have little data on the perspective of most parents, qualitative research on parental experiences with earlier and later operations could give important insights. This research can be initiated in collaboration with the ERNICA quality of life working group. Secondly, the pediatric surgeons on the panel emphasize that they don't know if the size of the abdomen plays a role in determining the optimal timing of surgery for each individual. Prospective data collection of abdominal and omphalocele circumference (for example every 3 months after birth) could contribute to new insights on the role of the size of the omphalocele and abdomen in relation to optimal timing. Furthermore, the panel would like to have the hypotheses about neurodevelopmental impact of surgeries in the first year of life tested in the omphalocele population. Insights from these research plans will likely contribute to the ability to revise the recommendation based on stronger conclusions in five years.

REFERENCES SUMMARY

1.Schneuer FJ, Bentley JP, Davidson AJ, Holland AJ, Badawi N, Martin AJ, Skowno J, Lain SJ, Nassar N. The impact of general anesthesia on child development and school performance: a population-based study. Paediatr Anaesth. 2018 Jun;28(6):528-536. doi: 10.1111/pan.13390. epub 2018 Apr 27. PMID: 29701278.

2.McCann ME, de Graaff JC, Dorris L, Disma N, Withington D, Bell G, Grobler A, Stargatt R, Hunt RW, Sheppard SJ, Marmor J, Giribaldi G, Bellinger DC, Hartmann PL, Hardy P, Frawley G, Izzo F, von Ungern Sternberg BS, Lynn A, Wilton N, Mueller M, Polaner DM, Absalom AR, Szmuk P, Morton N, Berde C, Soriano S, Davidson AJ; GAS Consortium. Neurodevelopmental outcome at 5 years of age after general anaesthesia or awake-regional anaesthesia in infancy (GAS): an international, multicentre, randomised, controlled equivalence trial. Lancet. 2019 Feb 16;393(10172):664-677. doi: 10.1016/s0140-6736(18)32485-1. epub 2019 Feb 14. Erratum in: Lancet. 2019 Aug 24;394(10199):638. doi: 10.1016/s0140-6736(19)31958-0. PMID: 30782342; PMCID: PMC6500739.

3.Kassa AM, Lilja HE. Neurodevelopmental outcomes in individuals with VACTERL association. A population-based cohort study. PLoS One. 2023 Jun 29;18(6):e0288061. doi: 10.1371/journal.pone.0288061. PMID: 37384789; PMCID: PMC10310046.

4.Kutasy B, Skoglund C, Löf-Granström A, Öst E, Frenckner B, Mesas Burgos C. Increased risk of clinically relevant neurodevelopmental disorders in survivors of congenital diaphragmatic hernia: a population-based study. Pediatr Surg Int. 2024 Nov 11;40(1):304. doi: 10.1007/s00383-024-05871-1. PMID: 39528855.

5.Raj M, Chattopadhyay A, Gupta SK, Jain S, Sastry UMK, Sudevan R, Sharma M, Pragya P, Shivashankar R, Sudhakar A, Radhakrishnan A, Parveen S, Patil S, Naik S, Das S, Kumar RK. Neurodevelopmental outcomes after infant heart surgery for congenital heart disease: a hospital-based multicentre prospective cohort study from India. BMJ Paediatr Open. 2025 Jan 21;9(1):e002943. doi: 10.1136/bmjpo-2024-002943. PMID: 39842864.

6.Jiang Y, Zhou Y, Tan S, Xu C, Ma J. Front Pharmacol. 2023 Mar 13;14:1113345. doi: 10.3389/fphar.2023.1113345. eCollection 2023. PMID: 36992831.
