# Supplementary material for: ERNICA evidence based guideline on omphalocele
Source: Orphanet J Rare Dis. 2026 Mar 7;21:193. doi: 10.1186/s13023-026-04293-7 (PMC13162432; doi:10.1186/s13023-026-04293-7)
Supplement: Supplementary file 4 — Supplementary Material 4 [file 13023_2026_4293_MOESM4_ESM.pdf]

Supplement 4. Search Strategy

Database searched	Platform	Years of coverage	Records	Records after duplicates removed
Medline ALL	Ovid	1946 - Present	501	500
Embase	Embase.com	1971 - Present	480	134
Web of Science Core Collection*	Web of Knowledge	1975 - Present	417	48
Cochrane Central Register of Controlled Trials	Wiley	1992 - Present	5	1
CINAHL Plus	EBSCO	1982 - Present	126	19
**Total**			**1529**	**702**

*Science Citation Index Expanded (1975-present) ; Social Sciences Citation Index (1975-present) ; Arts & Humanities Citation Index (1975-present) ; Conference Proceedings Citation Index- Science (1990-present) ; Conference Proceedings Citation Index- Social Science & Humanities (1990-present) ; Emerging Sources Citation Index (2005-present)

No other database limits were used than those specified in the search strategies

medline 501

((omphalocel* OR exomphalo*).ti.) AND (exp Cohort Studies/ OR Case Reports / OR exp Clinical Trial / OR Observational Study / OR Systematic Review/ OR Meta-Analysis / OR exp Clinical Study/ OR Case-Control Studies/ OR Multicenter Study/ OR * Outcome Assessment, Health Care/ OR * Patient Outcome Assessment/ OR exp * Treatment Outcome/ OR exp * Prenatal Diagnosis/ OR * Genetic Testing/ OR * Genetic Association Studies/ OR exp * Survival/ OR exp * Mortality/ OR * Karyotyping/ OR exp * Sequence Analysis/ OR exp * Lung Volume Measurements/ OR ((systematic* ADJ3 review*) OR meta-analy* OR metaanaly* OR cohort* OR longitudinal* OR prospectiv* OR retrospectiv* OR case-series* OR case-stud* OR case-control* OR trial* OR random* OR ((observation* OR clinical*) ADJ3 stud*) OR multicenter* OR multi-center* OR (case* ADJ3 review*) OR ((prenatal* OR pre-natal* OR perinatal* OR peri-natal*) ADJ3 (diagnos* OR screen*)) OR (genetic* ADJ3 (screen* OR diagnos* OR test* OR associat*)) OR karyotyp* OR (sequenc* ADJ3 (analys* OR exome*)) OR ((lung OR pulmonar*) ADJ3 (volume* OR hypoplasia*))).ab,ti,kw. OR (study OR cases OR review* OR outcome* OR management* OR prenatal* OR pre-natal* OR perinatal* OR peri-natal* OR genetic* OR associat* OR surviv* OR mortalit*).ti.) AND 2000:2024.(sa_year). AND english.la.

embase 480

(omphalocele/mj OR (omphalocel* OR exomphalo*):ti) AND ('cohort analysis'/de OR 'longitudinal study'/de OR 'prospective study'/de OR 'retrospective study'/de OR 'case study'/de OR 'clinical trial'/exp OR 'observational study'/de OR 'systematic review'/de OR 'meta analysis'/de OR 'clinical study'/de OR 'case control study'/exp OR 'intervention study'/de OR 'major clinical study'/de OR 'multicenter study'/de OR 'outcome assessment'/mj OR 'treatment outcome'/exp/mj OR 'prenatal diagnosis'/mj OR 'prenatal screening'/mj OR 'genetic screening'/mj OR 'genetic diagnosis'/mj OR 'genetic association'/mj OR survival/exp/mj OR mortality/exp/mj OR karyotyping/mj OR 'sequence analysis'/mj OR 'lung volume'/mj OR 'lung hypoplasia'/mj OR ((systematic* NEAR/3 review*) OR meta-analy* OR metaanaly* OR cohort* OR longitudinal* OR prospectiv* OR retrospectiv* OR case-series* OR case-stud* OR case-control* OR trial* OR random* OR ((observation* OR clinical*) NEAR/3 stud*) OR multicenter* OR multi-center* OR (case* NEAR/3 review*) OR ((prenatal* OR pre-natal* OR perinatal* OR peri-natal*) NEAR/3 (diagnos* OR screen*)) OR (genetic* NEAR/3 (screen* OR diagnos* OR test* OR associat*)) OR karyotyp* OR (sequenc* NEAR/3 (analys* OR exome*)) OR ((lung OR pulmonar*) NEAR/3 (volume* OR hypoplasia*))):ab,ti,kw OR (study OR cases OR review* OR outcome* OR management* OR prenatal* OR pre-natal* OR perinatal* OR peri-natal* OR genetic* OR associat* OR surviv* OR mortalit*):ti) AND [2000-2024]/py NOT [conference abstract]/lim AND [english]/lim

Web of science 417

(TI=(omphalocel* OR exomphalo*)) AND (TS=((systematic* NEAR/2 review*) OR meta-analy* OR metaanaly* OR cohort* OR longitudinal* OR prospectiv* OR retrospectiv* OR case-series* OR case-stud* OR case-control* OR trial* OR random* OR ((observation* OR clinical*) NEAR/2 stud*) OR multicenter* OR multi-center* OR (case* NEAR/2 review*) OR ((prenatal* OR pre-natal* OR perinatal* OR peri-natal*) NEAR/2 (diagnos* OR screen*)) OR (genetic* NEAR/2 (screen* OR diagnos* OR test* OR associat*)) OR karyotyp* OR (sequenc* NEAR/2 (analys* OR exome*)) OR ((lung OR pulmonar*) NEAR/2 (volume* OR hypoplasia*))) OR TI=(study OR cases OR review* OR outcome* OR management* OR prenatal* OR pre-natal* OR perinatal* OR peri-natal* OR genetic* OR associat* OR surviv* OR mortalit*)) NOT DT=(Meeting Abstract OR Meeting Summary) AND PY=(2000-2024)

Cochrane 5

((omphalocel* OR exomphalo*):ti) NOT ("conference abstract":kw OR Trial registry record:pt)

CINAHL 126

TI((omphalocel* OR exomphalo*)) AND (MH Prospective Studies + OR MH Case Studies + OR MH Clinical Trials + OR MH Nonexperimental Studies OR MH Systematic Review OR MH Meta Analysis OR MH Case Control Studies+ OR MH Multicenter Studies OR MM "Outcomes (Health Care)" OR MM Outcome Assessment+ OR MM Treatment Outcomes+ OR MM Prenatal Diagnosis+ OR MM Genetic Screening + OR MM Genome Wide Association Study + OR MM Survival+ OR MM Mortality+ OR MM Karyotyping+ OR MM Sequence Analysis+ OR MM Lung Volume Measurements+ OR TI((systematic* N2 review*) OR meta-analy* OR metaanaly* OR cohort* OR longitudinal* OR prospectiv* OR retrospectiv* OR case-series* OR case-stud* OR case-control* OR trial* OR random* OR ((observation* OR clinical*) N2 stud*) OR multicenter* OR multi-center* OR (case* N2 review*) OR ((prenatal* OR pre-natal* OR perinatal* OR peri-natal*) N2 (diagnos* OR screen*)) OR (genetic* N2 (screen* OR diagnos* OR test* OR associat*)) OR karyotyp* OR (sequenc* N2 (analys* OR exome*)) OR ((lung OR pulmonar*) N2 (volume* OR hypoplasia*))) OR AB((systematic* N2 review*) OR meta-analy* OR metaanaly* OR cohort* OR longitudinal* OR prospectiv* OR retrospectiv* OR case-series* OR case-stud* OR case-control* OR trial* OR random* OR ((observation* OR clinical*) N2 stud*) OR multicenter* OR multi-center* OR (case* N2 review*) OR ((prenatal* OR pre-natal* OR perinatal* OR peri-natal*) N2 (diagnos* OR screen*)) OR (genetic* N2 (screen* OR diagnos* OR test* OR associat*)) OR karyotyp* OR (sequenc* N2 (analys* OR exome*)) OR ((lung OR pulmonar*) N2 (volume* OR hypoplasia*))) OR TI(study OR cases OR review* OR outcome* OR management* OR prenatal* OR pre-natal* OR perinatal* OR peri-natal* OR genetic* OR associat* OR surviv* OR mortalit*)) AND PY 2000-2024 AND LA(english)

Specified search for mode of delivery

medline	76	76
embase	77	30
**Total**	**153**	**106**

medline 76

(Hernia, Umbilical / OR Hernia, Abdominal / OR (omphalocele* OR (congenital* ADJ3 (amniocele* OR eventrat*)) OR ((abdom* OR ventral*) ADJ3 wall* ADJ3 (defect* OR lesion* OR aplasia* OR rupture*)) OR ((umbilic* OR abdom*) ADJ3 hernia*)).ti.) AND (exp * Delivery, Obstetric / OR exp * Parturition/ OR * Vaginal Birth after Cesarean/ OR * Pregnancy Outcome/ OR Delivery Rooms / OR exp * Obstetrics/ OR (deliver* OR childbirth* OR obstetric* OR ((birth*) ADJ3 (mode OR vagina* OR operative* OR obstetric* OR

outcome*)) OR caesarea* OR cesarea* OR c-section* OR live-birth* OR livebirth* OR still-birth* OR stillbirth* OR still-born* OR stillborn* OR parturition*).ti.)

embase 77

(omphalocele/mj OR 'umbilical hernia'/mj OR 'abdominal wall defect'/mj OR 'abdominal wall hernia'/mj OR (omphalocele* OR (congenital* NEAR/3 (amniocele* OR eventrat*)) OR ((abdom* OR ventral*) NEAR/3 wall* NEAR/3 (defect* OR lesion* OR aplasia* OR rupture*)) OR ((umbilic* OR abdom*) NEAR/3 hernia*)):ti) AND ('obstetric delivery'/mj/exp OR 'pregnancy outcome'/mj OR 'delivery room'/mj OR obstetrics/exp/mj OR (deliver* OR childbirth* OR obstetric* OR ((birth*) NEAR/3 (mode OR vagina* OR operative* OR obstetric* OR outcome*)) OR caesarea* OR cesarea* OR c-section* OR live-birth* OR livebirth* OR still-birth* OR stillbirth* OR still-born* OR stillborn* OR parturition*):ti) NOT [conference abstract]/lim AND [english]/lim
