# Supplementary material for: ERNICA evidence based guideline on omphalocele
Source: Orphanet J Rare Dis. 2026 Mar 7;21:193. doi: 10.1186/s13023-026-04293-7 (PMC13162432; doi:10.1186/s13023-026-04293-7)
Supplement: Supplementary file 5 — Supplementary Material 5 [file 13023_2026_4293_MOESM5_ESM.pdf]

Supplement 5

Omphalocele definitions

There is no consensus about the definitions used to describe omphalocele giant and non-giant omphalocele subtypes. Without the intention to standardize the use of these terms, the definitions used for the guideline are listed below.

Giant Omphalocele

Giant omphalocele in this guideline refers to defects that cannot be closed primarily and have a diameter of ≥5cm where the liver is (partially) herniated. As 'ability for primary closure' could not be found as a patient characteristic in the omphalocele literature, studies could be included for the analysis of questions on giant omphalocele if the population was described as omphalocele ≥5cm and herniated liver. As the available literature was scarce, the panel opted to also include studies that described their population as Omphalocele ≥5cm and/or herniated liver. Any studies that referred to Giant Omphalocele with no further specification of the characteristics were excluded. In the analysis of EPSA data, patients were included if they were registered by the physician as giant omphalocele and had liver herniation.

Non-Giant Omphalocele

Non-Giant Omphalocele in this guideline refers to defects that are <5cm and typically do not contain liver. As the available literature was scarce and the size of the non-giant omphalocele seemed to be missing in a lot of studies, the panel opted to include studies that described their population as small omphalocele or non-giant omphalocele without herniated liver. Any studies that referred to non-giant or small omphalocele without any further description or included patients with herniated liver as non-giant omphalocele, were excluded. In the analysis of EPSA data, patients were included if they were registered by the physician as non-giant omphalocele and had no liver herniation.

Treatment definitions

In this guideline, we distinguish between various forms of closure of the abdominal wall. There are many treatments available for omphalocele, and their terminology is not uniform. Without the intention to standardize the use of these terms, the definitions used for the guideline are listed below.

Primary Surgical Closure (PSC)

PSC is a procedure in which the herniated contents are reduced back into the abdominal cavity, and the abdominal wall defect is closed directly. Usually the herniated viscera is repositioned and the cele wall are resected, while abdominal layers including the fascia are repaired [1, 2] . Excessive tension on the abdominal wall should be avoided as this can impair respiratory and circulatory function. This treatment is most suitable for small to moderate-sized omphaloceles without significant associated anomalies.

Staged Reduction and Surgical Repair (SRSR):

SRSR involves a gradual reduction of the herniated contents using a silo or other method over several days to weeks, followed by definitive closure of the abdominal wall. This method allows the abdominal cavity to gradually accommodate the displaced organs. Staged reduction, by definition, requires the use of multiple procedures prior to final abdominal wall fascial closure. SRSR can include measures to enhance the volume of the abdominal cavity before closing the abdominal wall as well as closing the abdominal wall in several steps [2]. In this guideline, literature describing the following methods was considered to answer questions about SRSR: (non) surgical silo, tissue expander placement (Fasciotens), traction, taping and the use of mesh [1]. All of these closure methods, as a staged surgical closure method, must be followed up by a subsequent operation to close the abdominal wall fascia later. SRSR is indicated for omphaloceles where primary closure is not feasible due to the risk of abdominal compartment syndrome.

Non-Operative management and Delayed closure (NOM)

This method is defined as abdominal tissue closure by epithelialization of the sac, with or without topical medication applied directly onto the omphalocele membrane to promote the formation of eschar, followed by granulation and neo-epithelialization[1]. Non-operative management and delayed closure, is also referred to as 'paint and wait' or 'stain and wait[3].

Closure methods for the ventral hernia are primary, staged, skin-only or synthetic patch closure and this is done at various ages of the patient, typically ranging from the neonatal period to around 12 months of age or even later [4] . NOM is used if immediate surgery is not possible due to the infant's condition or the size of the defect, especially for high-risk giant omphaloceles or in patients with lung hypoplasia[5]. It may facilitate early enteral feeding, and is said to minimize respiratory compromise, and reduce morbidity and mortality[2, 4].

[1] Bauman B, Stephens D, Gershone H, Bongiorno C, Osterholm E, Acton R, et al. Management of giant omphaloceles: A systematic review of methods of staged surgical vs. nonoperative delayed closure. J Pediatr Surg 2016;51(10):1725-30.
[2] Mack AJ, Rogdo B. Giant omphalocele: current perspectives. Research and Reports in Neonatology 2016:33-9.
[3] Kogut KA, Fiore NF. Nonoperative management of giant omphalocele leading to early fascial closure. J Pediatr Surg 2018;53(12):2404-8.
[4] Lee SL, Beyer TD, Kim SS, Waldhausen JH, Healey PJ, Sawin RS, Ledbetter DJ. Initial nonoperative management and delayed closure for treatment of giant omphaloceles. J Pediatr Surg 2006;41(11):1846-9.
[5] Wagner JP, Cusick RA. Paint and wait management of giant omphaloceles. Semin Pediatr Surg 2019;28(2):95-100.
